# Hollow Mo/MoS_Vn_ Nanoreactors with Tunable Built‐in Electric Fields for Sustainable Hydrogen Production

**DOI:** 10.1002/adma.202415269

**Published:** 2024-12-08

**Authors:** Feilong Gong, Zhilin Chen, Chaoqun Chang, Min Song, Yang Zhao, Haitao Li, Lihua Gong, Yali Zhang, Jie Zhang, Yonghui Zhang, Shizhong Wei, Jian Liu

**Affiliations:** ^1^ Key Laboratory of Surface and Interface Science and Technology of Henan Province College of Material and Chemical Engineering Zhengzhou University of Light Industry Zhengzhou Henan 450000 P. R. China; ^2^ State Key Laboratory of Catalysis Dalian Institute of Chemical Physics Chinese Academy of Sciences Dalian Liaoning 116023 P. R. China; ^3^ College of Chemistry and Chemical Engineering Inner Mongolia University Hohhot Inner Mongolia 010021 P. R. China; ^4^ School of Economics and Management Inner Mongolia University Hohhot Inner Mongolia 010021 P. R. China; ^5^ DICP‐Surrey Joint Centre for Future Materials Department of Chemical and Process Engineering and Advanced Technology Institute of University of Surrey Guildford Surrey GU2 7XH UK

**Keywords:** built‐in electric field, hollow nanoreactors, hydrogen evolution reaction, urea oxidation reaction

## Abstract

Constructing built‐in electric field (BIEF) in heterojunction catalyst is an effective way to optimize adsorption/desorption of reaction intermediates, while its precise tailor to achieve efficient bifunctional electrocatalysis remains great challenge. Herein, the hollow Mo/MoS_Vn_ nanoreactors with tunable BIEFs are elaborately prepared to simultaneously promote hydrogen evolution reaction (HER) and urea oxidation reaction (UOR) for sustainable hydrogen production. The BIEF induced by sulfur vacancies can be modulated from 0.79 to 0.57 to 0.42 mV nm^−1^, and exhibits a parabola‐shaped relationship with HER and UOR activities, the Mo/MoS_V1_ nanoreactor with moderate BIEF presents the best bifunctional activity. Theoretical calculations reveal that the moderate BIEF can evidently facilitate the hydrogen adsorption/desorption in the HER and the breakage of N─H bond in the UOR. The electrolyzer assembled with Mo/MoS_V1_ delivers a cell voltage of 1.49 V at 100 mA cm^−2^, which is 437 mV lower than that of traditional water electrolysis, and also presents excellent durability at 200 mA cm^−2^ for 200 h. Life cycle assessment indicates the HER||UOR system possesses notable superiority across various environment impact and energy consumption. This work can provide theoretical and experimental direction on the rational design of advanced materials for energy‐saving and eco‐friendly hydrogen production.

## Graphical abstract

The built‐in electric fields (BIEFs) in the hollow Mo/MoS_Vn_ nanoreactors can be gradiently modulated via tailoring the sulfur vacancies, and exhibit parabola‐shaped relationship with HER and UOR activities. Moderate BIEF evidently facilitates the hydrogen desorption in the HER and the breakage of N─H bond in the UOR, endowing the Mo/MoS_V1_ electrolyzer with superior activity and durability.

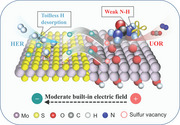

## 1 Introduction

The development of high‐efficiency water electrolysis device is of great value to promote sustainable energy conversion for realizing carbon neutrality.^[^ ^1^ ^]^ However, the sluggish kinetics and high theoretical potential of oxygen evolution reaction (OER) on anode usually lead to excess energy consumption.^[^ ^2^ ^]^ Replacing OER with effortless thermodynamic reaction like urea,^[^ ^3^ ^]^ methanol,^[^ ^4^ ^]^ glycerol,^[^ ^5^ ^]^ hydrazine electrooxidations,^[^ ^6^ ^]^ etc. is considered as an efficient strategy for green hydrogen production at low voltages. Specifically, urea oxidation reaction (UOR) is a competitive option because of (i) 70% energy saving theoretically than traditional water electrolysis,^[^ ^7^ ^]^ (ii) abundant resources in industrial sewage, agricultural wastewater, and human urine (approximately 0.33 M),^[^ ^8^ ^]^ and (iii) purification of urea‐rich water to alleviate environment problems such as water eutrophication, soil and air pollution.^[^ ^5^, ^9^ ^]^ Therefore, coupling hydrogen evolution reaction (HER) with UOR has great potential to realize energy‐saving hydrogen production and urea‐containing wastewater treatment simultaneously. Whereas, the urea electrooxidation shows slow dynamic process intrinsically subjecting to the high energy barriers of consecutive hydrogen desorption for N‐H,^[^ ^10^ ^]^ thus it is essential but challenging to design efficient and stable bifunctional electrocatalysts serving for HER||UOR system.

Noble metal‐based catalysts are well known for excellent catalytic activity, while high price and scarce reserves restrict their large‐scale application.^[^ ^11^ ^]^ A variety of non‐precious metal‐based catalysts have been developed for HER, OER, and UOR, such as oxides,^[^ ^12^ ^]^ sulfides,^[^ ^13^ ^]^ carbides,^[^ ^14^ ^]^ high‐entropy materials,^[^ ^15^ ^]^ etc. Nevertheless, in most cases, the active sites are single or insufficient, thus making the catalysts lack of satisfactory performances for coupling HER and OER or UOR, which increases the cost of electrolysis and reduces the production efficiency. Up to now, the strategies, including heteroatom doping,^[^ ^16^ ^]^ defect engineering,^[^ ^17^ ^]^ configuration modulation,^[^ ^18^ ^]^ heterostructure construction,^[^ ^19^ ^]^ etc, have been tried to improve above issues. For example, the urea‐assisted water electrolyzer assembled by heteroatoms‐doped hybrids could output 10 mA cm^−2^ at a relatively low cell voltage not exceeding 1.40 V.^[^ ^20^ ^]^ The heterojunction catalyst with more active sites exhibited a markedly reduced driving voltage to reach 100 mA cm^−2^ compared with the single‐phase catalysts in urea‐assisted hydrogen evolution system.^[^ ^21^ ^]^ In addition, the multi‐level nanostructures could run stably at 500 mA cm^−2^ for ca. 20 h in the HER||UOR system.^[^ ^22^ ^]^ Although a lot of research has been conducted, a gap for the practical application requirements is still existed. The high cell voltage and poor durability of the HER||UOR system at large current densities are still the key problems.^[^ ^23^ ^]^

Among the valuable methods, constructing heterostructures can induce the formation of built‐in electric field (BIEF) due to distinguishing work functions (WFs) of different components, which can promote electron transfer and regulate species adsorption to facilitate reaction kinetics.^[^ ^24^ ^]^ For instance, the biphasic metal nitrides with highly lattice‐matched structures were built to create an enhanced BIEF between Ni_3_N and Co_3_N, and the resultant bifunctional catalyst saved over 53.3% energy consumption.^[^ ^25^ ^]^ Through construction of a BIEF by combining Co‐metal organic framework with CuO arrays, the OH^−^ and glycerol adsorption were enhanced, resulting in superior glycerol‐assisted hydrogen production performances with a high glycerol conversion and Faradaic efficiency.^[^ ^26^ ^]^ Benefitting from the strong electron interactions at the heterointerface that derived by BIEF, the electrocatalytic coupling system assembled with bifunctional catalyst presented exceptional sulfur degradation‐assisted water splitting properties, including a low electricity consumption and excellent stability.^[^ ^27^ ^]^ For the HER||UOR system, the BIEF in the heterocatalysts can not only facilitate OH^−^ adsorption in the electron‐poor region resulting in the formation of oxide/hydroxide active species in the UOR,^[^ ^3^ ^]^ but also regulate the electronic structure and optimize the hydrogen adsorption in the HER,^[^ ^28^ ^]^ which well meets the design requirements for efficient bifunctional catalysts. Besides of the above Co‐ and Ni‐based heterocatalysts, Mo‐based compounds are also widely utilized as HER and UOR electrocatalysts due to the merits of the abundance, the coexistence of empty and occupied d orbitals in the electronic structures.^[^ ^8^, ^29^ ^]^ Recently, it is reported that hollow/core‐shell/porous structured nanoreactors as electrocatalysts can accelerate accumulation of reactants, regulate mass transfer, and confine the conversion of the reactants and intermediates in the void space, which is significant to improve the catalytic reaction microenvironment.^[^ ^30^ ^]^ Up to now, researches mainly focus on developing enhanced electrocatalysts via the construction of BIEF, while the gradient modulation of BIEF in the hollow Mo‐based nanoreactor to better balance adsorption and desorption of reaction intermediates in both HER and UOR process is of significant challenge, and the corresponding enhancement mechanism and environment impact evaluation are also ambiguous.

Herein, the BIEFs intensity were gradiently modulated via regulating the concentrations of sulfur vacancies in the hollow nanoreactors (Mo/MoS_2_, Mo/MoS_V1_, and Mo/MoS_V2_) to achieve a better balance between the adsorption and desorption of reaction intermediates for efficient and sustainable hydrogen production in simulated urea wastewater. The electrochemical tests revealed that the activities of the nanoreactors toward HER and UOR exhibited parabola‐shaped relationship with the BIEF intensity, with Mo/MoS_V1_ presenting the lowest potentials. Density functional theory (DFT) calculations indicated that the moderate BIEF steered the lowest Mo─H bond energy in the HER and the weakest N─H bonding strength at the rate‐determining step in the UOR, endowing Mo/MoS_V1_ nanoreactor with superior bifunctional activity. Impressively, the urea‐assisted water electrolyzer fabricated with the Mo/MoS_V1_ delivered much lower cell voltage than that of traditional water electrolysis, surpassing most reported electrocatalysts, and also presented excellent durability at a high current density. Life cycle assessment (LCA) confirmed the HER||UOR system exhibited obviously lower environmental impact and energy consumption comparing to the HER||OER system and seawater electrolysis.

## 2 Results and Discussion

### 2.1 Synthesis and Characterizations of Mo/MoS_Vn_ Nanoreactors

**Figure 1:**
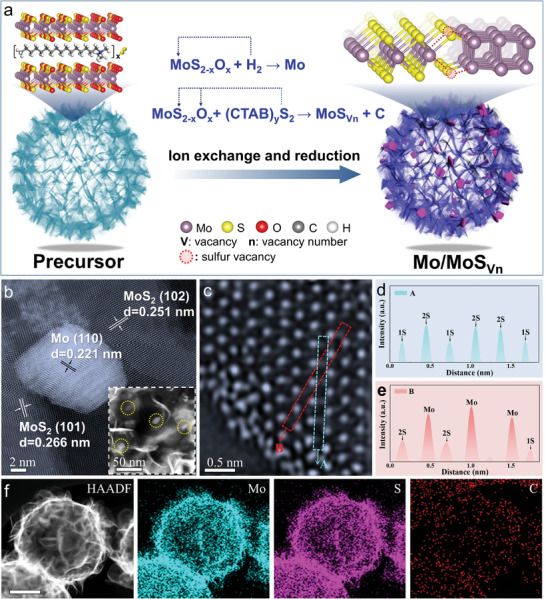
Synthesis scheme and morphology characterizations for Mo/MoS_Vn_ nanoreactors. a) Schematic diagram of preparation process. b,c) AC‐TEM images, d,e) the atom intensity profiles along the cyan (A) and red (B) frame of graph (c), and f) HAADF and corresponding EDS mapping images of the Mo/MoS_V1_. The scale bar in (f) is 100 nm.

The precursors (Precursor‐10, Precursor‐20, and Precursor‐40), consisting of MoS_2‐x_O_x_ and different amounts of pre‐encapsulated (CTAB)_y_S_2_, were firstly prepared via regulating the addition of thiourea, and subsequently converted to the Mo/MoS_Vn_ by one‐step ion exchange and reduction technology (**Figure** **1**a; Note S1, Supporting Information). For comparison, hollow MoS₂ and Mo microspheres were also prepared (Notes S2, S3, Supporting Information). The field emission scanning electron microscopy (FESEM) images indicated uniform wrinkled and smooth microspheres configurations of the precursors with and without sulfur source, respectively (Figure S1, Supporting Information). The X‐ray diffraction (XRD) profiles showed typical peaks of carbon and 2H‐MoS_2_ (Figure S2, Supporting Information). The peak at 7.3° assigned to expanded layer, which exhibited a gradually positive shift with the increase of sulfur source. The precursor without sulfur source was identified as MoO_2_ (JCPDS No.50‐0739) and carbon (Figure S3, Supporting Information). Moreover, the existent of Mo, S, C, and O were identified by the X‐ray photoelectron spectroscopy (XPS) survey and high‐resolution spectra (Figures S4–S8, Supporting Information). Specially, the percentages of S_2_^2−^ in the (CTAB)_y_S_2_ were calculated to be 31.9%, 36.9%, and 39.6% for the Precursor‐10, Precursor‐20, and Precursor‐40, respectively, which could be utilized to regulate the concentration of sulfur vacancy in the Mo/MoS_Vn_ nanoreactors. As shown in Figure S9 (Supporting Information) all XRD peaks were well assigned to Mo (JCPDS No.42‐1120) and MoS_2_ (JCPDS No.37‐1492) for the Mo/MoS_Vn_. In the Raman spectra (Figure S10, Supporting Information), the vibration modes of E^1^_2__g_ and A_1__g_ of 2H‐MoS_2_ were detected in the Mo/MoS_Vn_. The FESEM and transmission electron microscopy (TEM) images showed hollow structure. The lattice spacings of 0.221, 0.266, and 0.251 nm were corresponding to the (110) facet of Mo, and (101) and (102) facets of MoS_2_, respectively, in the high‐resolution TEM (HRTEM) images (Figures S11–S14, S16, Supporting Information). The N_2_ absorption and desorption curves indicated the mesoporous structure of the Mo/MoS_V1_ nanoreactor (Figure S17, Supporting Information). According to the low magnification spherical aberration corrected transmission electron microscopy (AC‐TEM) image in Figure 1b, Mo nanoparticles dispersed on the hollow MoS_2_ support. The high magnification AC‐TEM image was also conducted to identify atomic states (Figure 1c; Figure S15, Supporting Information). Correspondingly, the atom intensity profiles along the cyan (A) and red (B) frame in Figure 1c were presented in Figure 1d,e demonstrating the existence of sulfur vacancies in the Mo/MoS_V1_. The high‐angle annular dark field (HAADF) and corresponding energy‐dispersive X‐ray spectroscopy (EDS) mapping images revealed the uniform distribution of Mo, S, and C elements in the Mo/MoS_V1_ (Figure 1f).

### 2.2 Chemical State and Electronic Structure Analysis

**Figure 2:**
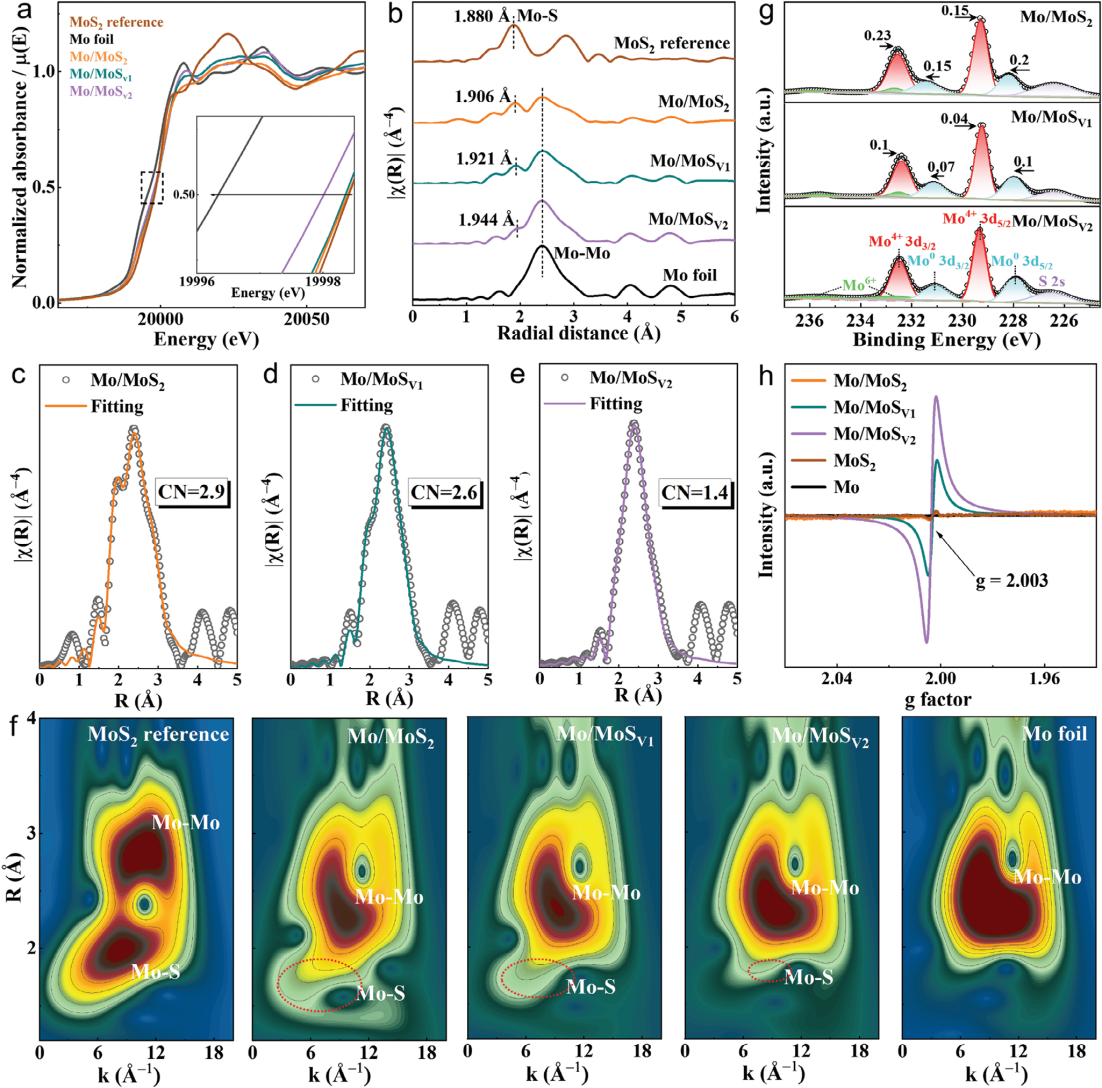
The chemical state and electronic structure information of Mo/MoS_Vn_ nanoreactors. a) Normalized XANES spectra at Mo K‐edge. The inset of a) is the magnified pre‐edge XANES region. b) FT‐EXAFS spectra. c–e) Fitting curves of EXAFS spectra for Mo at R space. f) The corresponding WT of the k^3^‐weighted Mo K‐edge EXAFS signal of MoS_2_ reference, Mo/MoS_Vn_, and Mo foil. g) Comparisons of high‐resolution Mo 3d XPS spectra. h) Comparisons of EPR spectra.

The X‐ray absorption spectroscopy (XAS) at Mo K‐edge was collected to further investigate the difference of sulfur vacancy in the Mo/MoS_Vn_. As shown in **Figure** **2**a, the Mo K‐edge X‐ray absorption near edge structure (XANES) spectra of the Mo/MoS_2_, Mo/MoS_V1_, and Mo/MoS_V2_ were all located between Mo foil and MoS_2_ reference. Based on the curve of linear relation obtained using the area integration method, the average valence states of Mo were +3.92, +3.84, and +3.34 in the Mo/MoS_2_, Mo/MoS_V1_, and Mo/MoS_V2_, respectively (Figure S18, Supporting Information), indicating the different atomic coordination around Mo atom. Fourier‐transformation extended X‐ray absorption fine structure (FT‐EXAFS) spectra displayed that the Mo‐Mo peak locations in the Mo/MoS_Vn_ were basically the same as that in the Mo foil (Figure 2b). By contrast, the Mo‐S peak locations showed a gradually positive shift to 1.906, 1.921, and 1.944 Å in the Mo/MoS_2_, Mo/MoS_V1_, and Mo/MoS_V2_ compared with that in the MoS_2_ reference (1.880 Å), which could attribute to the formation of heterostructures and the different coordination environment of S around Mo. The related fitting curves of EXAFS spectra at R space and K space were plotted to check the chemical coordination structures of Mo in the Mo/MoS_Vn_ (Figure 2c–e; Figures S19–S21, Supporting Information), and detailed parameters extracted from the EXAFS fitting were presented in Table S1 (Supporting Information). As a result, 1 Mo atom coordinated with 2.9, 2.6, and 1.4 S atoms in the Mo/MoS_2_, Mo/MoS_V1_, and Mo/MoS_V2_, respectively, signifying the different amounts of sulfur vacancies in the Mo/MoS_Vn_. Furthermore, the wavelet transform (WT) of EXAFS results revealed that the strength of Mo─Mo bond in the Mo/MoS_Vn_ increased gradually with the reduction of Mo‐S coordination number, and the strength of Mo─S bond became weak gradually (Figure 2f).

According to the high‐resolution Mo 3d XPS spectra shown in Figure 2g, two couple peaks could be attributed to Mo^0^ and Mo^4+^ in the Mo/MoS_V2_, respectively. Notably, the Mo^0^ 3d peaks exhibited a gradual upshift, and Mo^4+^ 3d and S 2p peaks exhibited a gradual downshift in the Mo/MoS_V2_, Mo/MoS_V1_, and Mo/MoS_2_ compared with those in the Mo and MoS_2_ (Figures S22–S24, Supporting Information), indicating the electron transfer from Mo to MoS_Vn_. As for S 2p (Figure S24, Supporting Information), two characteristic peaks of S 2p_1/2_ and S 2p_3/2_ could be classified, and the S 2p_1/2_ peak corresponding to low coordination sulfur was related to sulfur vacancy. The concentration of sulfur vacancy was calculated based on the ration between S 2p_1/2_ and S 2p, and the Mo/MoS_V2_ nanoreactor exhibited higher sulfur vacancy concentration of 2.0% compared to the Mo/MoS_V1_ nanoreactor (1.7%). The XPS results revealed that the regulation of sulfur vacancy can induce the change of bonding environment in the MoS_Vn_. Meanwhile, the electron paramagnetic resonance (EPR) was employed to further validate the difference of sulfur vacancy in the nanoreactors. As shown in Figure 2h and Figure S26 (Supporting Information) the EPR signal at g‐factor of 2.003 in the Mo/MoS_V2_ was much stronger than that in the Mo/MoS_V1_ and no evident peak was detected in the Mo, MoS_2_, and Mo/MoS_2_, illustrating the tunable sulfur vacancies in the Mo/MoS_Vn_. According to the ultraviolet‐visible (UV–vis) diffuse reflection spectroscopy data (Figure S27, Supporting Information), the Mo/MoS_Vn_ showed the moderate band gaps compared with the Mo (0 eV) and MoS_2_ (1.26 eV), indicating that the heterointerfaces were benefit to supply optimized active sites with favorable adsorptions of reaction intermediates.^[^ ^31^ ^]^ The band gap increased from 0.36 to 0.86 to 1.08 eV for the Mo/MoS_V2_, Mo/MoS_V1_, and Mo/MoS_2_, confirming that the photoelectron mobility could be precisely tailored because of the different sulfur vacancies in the Mo/MoS_Vn_. Combining with above spectrum characterizations, the Mo atom bonded with S atom forming a Mo‐S unsaturated coordination structure in the Mo/MoS_Vn_, and the coordination number and sulfur vacancy content could be precisely tailored.

### 2.3 BIEFs Analysis for Mo/MoS_Vn_

**Figure 3:**
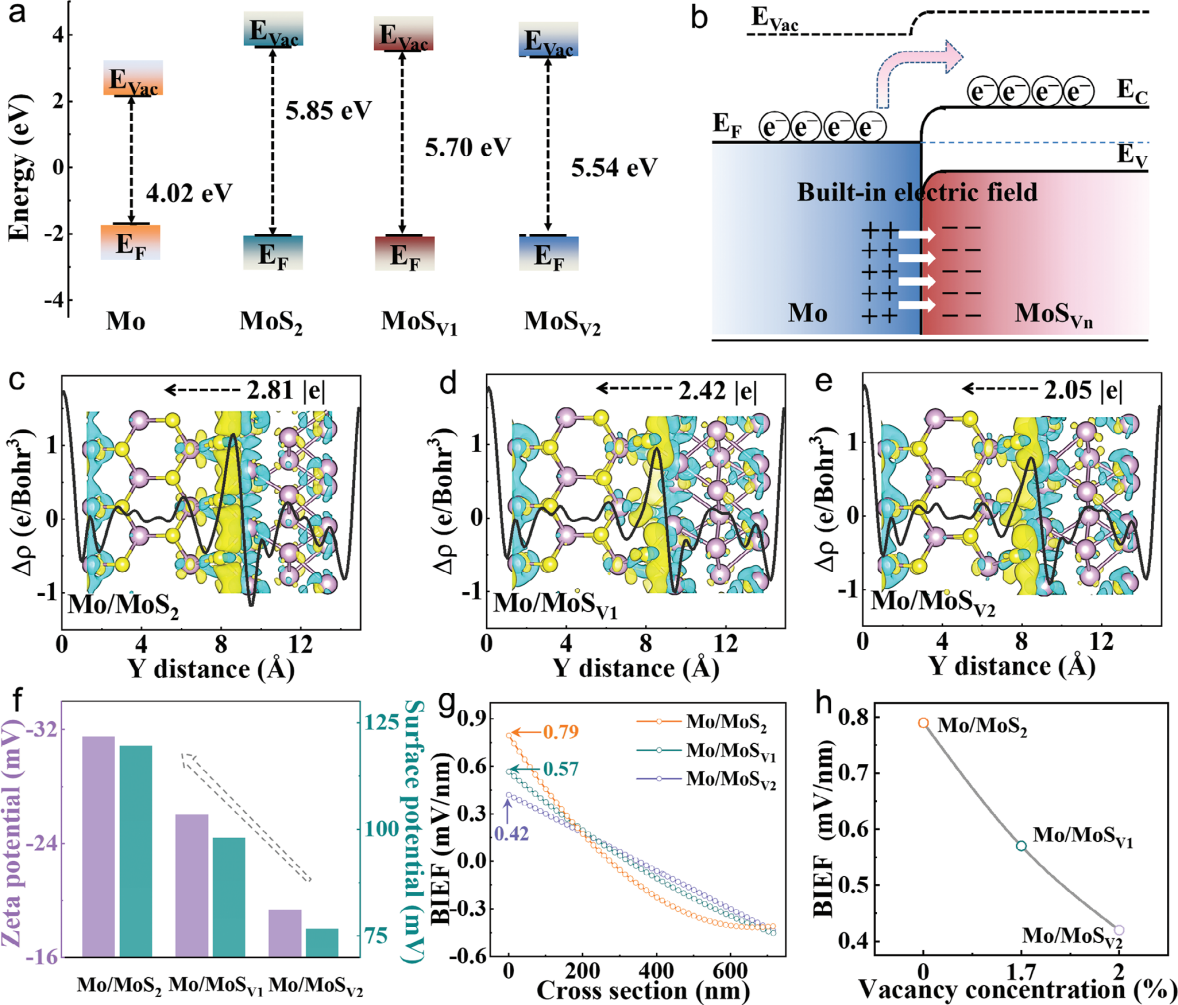
BIEFs analysis for Mo/MoS_Vn_. a) The WFs of Mo and Mo/MoS_Vn_ calculated by DFT. b) Schematic illustration of BIEF formation at the interface. c–e) The planar averaged charge density differences. Inset of c–e) are corresponding EDDs. The yellow and cyan regions indicate charge accumulation and depletion, respectively. f) The comparisons of Zeta potentials and surface potentials. g) The interfacial electric field intensity distributions. h) The relationship between sulfur vacancy concentration and BIEF intensity.

The models for Mo and MoS_Vn_ was built, and the corresponding value of WF was calculated to be 4.02 eV for Mo according to the DFT results, which was lower than that of MoS_2_ (5.85 eV), MoS_V1_ (5.70 eV) and MoS_V2_ (5.54 eV), implying the possible charge transfer from Mo to MoS_Vn_ (**Figure** **3**a). The different sulfur vacancies induced the differences of WF, leading to the formation of adjustable BIEFs at the interfaces between Mo and MoS_Vn_ (Figure 3b), which precisely modulated the interfacial microenvironment. The electron density differences (EDDs) and corresponding planar‐averaged charge density differences were investigated to reveal the charge transfer between Mo and MoS_Vn_ (Figure 3c–e). Bader charge analysis was further conducted to analysis the charge transfer. As shown in Figures S28–S30 (Supporting Information) and Tables S2–S4 (Supporting Information), the amounts of charge transfer were 2.81, 2.42, and 2.05|e| for the Mo/MoS_2_, Mo/MoS_V1_, and Mo/MoS_V2_, respectively, which indicated that the difference‐value of WFs for the Mo and MoS_Vn_ could regulate the charge transfer, potentially triggering distinct BIEF in the Mo/MoS_Vn_. The morphology of the Mo/MoS_Vn_ lateral heterostructures was investigated by the atomic force microscopy (AFM), indicating a particle‐rich rough surface over the entire regions (Figure S31, Supporting Information). According to the Zeta potentials measurements (Figure 3f; Figure S33, Supporting Information), the average potentials were −31.5, −26.0, and −19.3 mV for the Mo/MoS_2_, Mo/MoS_V1_, and Mo/MoS_V2_. Since the BIEF intensities could be assessed by surface potential,^[^ ^32^ ^]^ the surface potentials of the Mo/MoS_Vn_ nanoreactors were measured. Based on the Kelvin probe force microscopy (KPFM) results (Figure S32, Supporting Information), the contact potential difference‐value for the Mo/MoS_2_ was 119.5 mV, which was much higher than those of the Mo/MoS_V1_ (97.9 mV) and Mo/MoS_V2_ (76.5 mV). The fitting surface potential profiles were further collected in Figure S34 (Supporting Information), and the intensity of interfacial electric field was evaluated by differentiating the fitting surface potential profiles. As shown in Figure 3g, the Mo/MoS_2_ presented much stronger interfacial electric field of 0.79 mV nm^−1^ compared with the Mo/MoS_V1_ (0.57 mV nm^−1^) and Mo/MoS_V2_ (0.42 mV nm^−1^). Based on the above results, the BIEF intensity could be gradiently modulated via regulating the sulfur vacancy concentration. Specifically, the BIEF intensities gradually decreased from 0.79 to 0.42 mV nm^−1^ as the sulfur vacancy concentration increased from 0 to 2.0% (Figure 3h).

### 2.4 Electrocatalytic Performances and In Situ Characterizations

**Figure 4:**
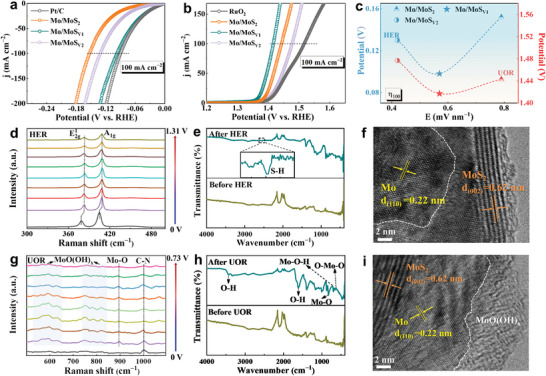
Electrocatalytic performances and in situ characterizations of Mo/MoS_Vn_. LSV curves of a) HER and b) UOR with 90% IR compensation. c) The comparisons of potentials at 100 mA cm^−2^ in different BIEFs. d) The in situ Raman spectra in the HER, e) the IR spectra before and after HER, and f) the HRTEM image after HER. g) The in situ Raman spectra in the UOR, h) the IR spectra before and after UOR, and i) the HRTEM image after UOR.

The HER properties of the as‐prepared nanoreactors were first investigated in alkaline media by a three‐electrode system. The linear sweep voltammetry (LSV) curves (**Figure** **4**a; Figure S35a, Supporting Information) showed that the Mo/MoS_V1_ required a lower overpotential of 98 mV to yield 100 mA cm^−2^ compared with the Mo/MoS_V2_ (130 mV) and Mo/MoS_2_ (152 mV), which was close to that of commercial Pt/C. Accordingly, the Tafel slope of Mo/MoS_V1_ (46.3 mV dec^−1^) was also smaller than those of the Mo/MoS_V2_, Mo/MoS_2_, MoS_2_, and Mo, and comparable to 42.6 mV dec^−1^ of Pt/C, indicating the fast catalytic kinetics (Figure S35b, Supporting Information). Notably, the LSV curves collected for the Mo/MoS_V1_ electrode with and without urea were almost overlapped (Figure S35c, Supporting Information), indicating that the urea molecules had no effect on HER activity. The electrochemical impedance spectra (EIS) fitting curves of the Mo/MoS_Vn_ (Figure S35d, Supporting Information) displayed the semicircular characteristics, and the Mo/MoS_V1_ presented smaller R_ct_ of 5.4 Ω compared with the Mo/MoS_V2_ (9.2 Ω), Mo/MoS_2_ (12.7 Ω), and MoS_2_ (17.3 Ω), meaning faster electron transport capability. As displayed in Figures S37, S38 (Supporting Information), the Mo/MoS_V1_ delivered the largest double‐layer capacitance (C_dl_) value of 40.1 mF cm^−2^ and the highest electrochemical surface area (ECSA) value of 1002.5 contrasting to those of the Mo/MoS_V2_, Mo/MoS_2_, MoS_2_, and Mo, demonstrating more exposed HER active sites. The comparison of HER overpotentials at 100 mA cm^−2^, Tafel slopes, EIS, and C_dl_ values for the Mo/MoS_Vn_ was concluded with a radar map (Figure S35e, Supporting Information), demonstrating the best electrocatalytic HER activity of Mo/MoS_V1_. In addition, the Mo/MoS_V1_ delivered an ultra‐low attenuation of 0.162 mA cm^−2^ h^−1^ at 350 mA cm^−2^ for 100 h, showing excellent stability (Figure S35f, Supporting Information). The nearly overlapped LSV curves of the initial and 1000th cycle further demonstrated the durability of the Mo/MoS_V1_ nanoreactor (Figure S36, Supporting Information).

The UOR performances were assessed in the electrolyte containing 1.0 M KOH and 0.33 M urea. The Mo/MoS_V1_ needed a much lower potential of 1.42 V to achieve 100 mA cm^−2^ than the Mo/MoS_2_ (1.44 V), Mo/MoS_V2_ (1.48 V), RuO_2_ (1.52 V), Mo (1.57 V), and MoS_2_ (1.68 V) (Figure 4b; Figure S39a,b, Supporting Information), confirming its outstanding catalytic activity. Moreover, the Tafel slope of the Mo/MoS_V1_ was much smaller than those of the Mo/MoS_2_, Mo/MoS_V2_, Mo, and MoS_2_, disclosing the rapider UOR kinetics (Figure S39c, Supporting Information). LSV curves of the Mo/MoS_V1_ in Figure S39d (Supporting Information) showed that the potential at 100 mA cm^−2^ was remarkably reduced by 134 mV after introducing urea. The Rct value of the Mo/MoS_V1_ was close to that of the Mo, and much smaller versus those of the Mo/MoS_2_, Mo/MoS_V2_, and MoS_2_ (Figure S39e, Supporting Information). The largest C_dl_ value and ECSA value of the Mo/MoS_V1_ endowed it with best intrinsic UOR activity (Figures S40, S41, Supporting Information). Chronoamperometry test was carried out for the Mo/MoS_V1_ at 200 mA cm^−2^ for 100 h with the periodic replenishment of fresh urea, and the immediate current restoration was observed for each period (Figure S39f, Supporting Information), proving the conspicuous UOR stability. The relationship between the HER and UOR potentials at 100 mA cm^−2^ with the BIEF intensity was presented in Figure 4c, which showed the parabola‐shaped feature with upward opening. Specially, the Mo/MoS_V1_ with a moderate BIEF intensity presented the lowest HER and UOR potentials, signifying the best bifunctional catalytic activity in the Mo/MoS_Vn_.

In situ Raman spectrum was employed to investigate the structure evolution of the Mo/MoS_V1_ in the HER and UOR processes. As shown in Figure 4d and Figure S42 (Supporting Information), with the increase of applied potential from 0 to 1.31 V in the HER process, the positions of the E^1^_2__g_ (378.3 cm^−1^) and A_1__g_ vibration (404.8 cm^−1^) belonging to 2H‐MoS_2_ exhibited a notable positive shift and the intensities gradually reduced, indicating that the HER active sites could be the MoS_V1_. Further, the Fourier Transform Infrared Spectroscopy (FT‐IR) results in Figure 4e showed the emerging of S─H bond at 2557 cm^−1^ after HER, demonstrating the S sites could interact with water molecules. The HRTEM images of the Mo/MoS_V1_ after HER indicated that there was no new phase appearing and the lattice fringes marked to the (110) facet of Mo and (002) facet of MoS_2_ were not destroyed, validating its structural stability (Figure 4f; Figure S44a, Supporting Information). Similarly, Figure 4g and Figure S43 (Supporting Information) displayed the in situ Raman results for UOR with the potentials from 0 to 0.73 V. The peak at 1001 cm^−1^ was assigned to the symmetric stretching of C‐N in urea, which showed a gradual decreased intensity along with the increase of applied voltage, suggesting continuous consumption of urea. Significantly, there was almost no shift in positions of the E^1^_2__g_ and A_1__g_ vibration assigned to 2H‐MoS_2_. By contrast, the Mo‐O symmetric stretching at 895 cm^−1^ emerged when applying voltages. The results revealed the oxidation reaction could happen on the Mo. Meanwhile, two broad peaks representing the MoO(OH)_x_ vibration modes in shaded areas were identified, revealing the structural reconstruction on the Mo in UOR process. Moreover, the FT‐IR peaks of O‐Mo‐O (633.0 cm^−1^), Mo‐O‐H (719.8 cm^−1^), Mo‐O (828.4 cm^−1^), and O‐H (1622.6 and 3428.4 cm^−1^) could be detected after UOR (Figure 4h), further demonstrating the formation of the MoO(OH)_x_ species in the Mo/MoS_V1_. The amorphous structures on the surface of Mo could be directly observed in the HRTEM images after UOR (Figure 4i; Figure S44b, Supporting Information), reconfirming the Mo could be the UOR active sites.

### 2.5 Theoretical Calculations and Enhancement Mechanisms

**Figure 5:**
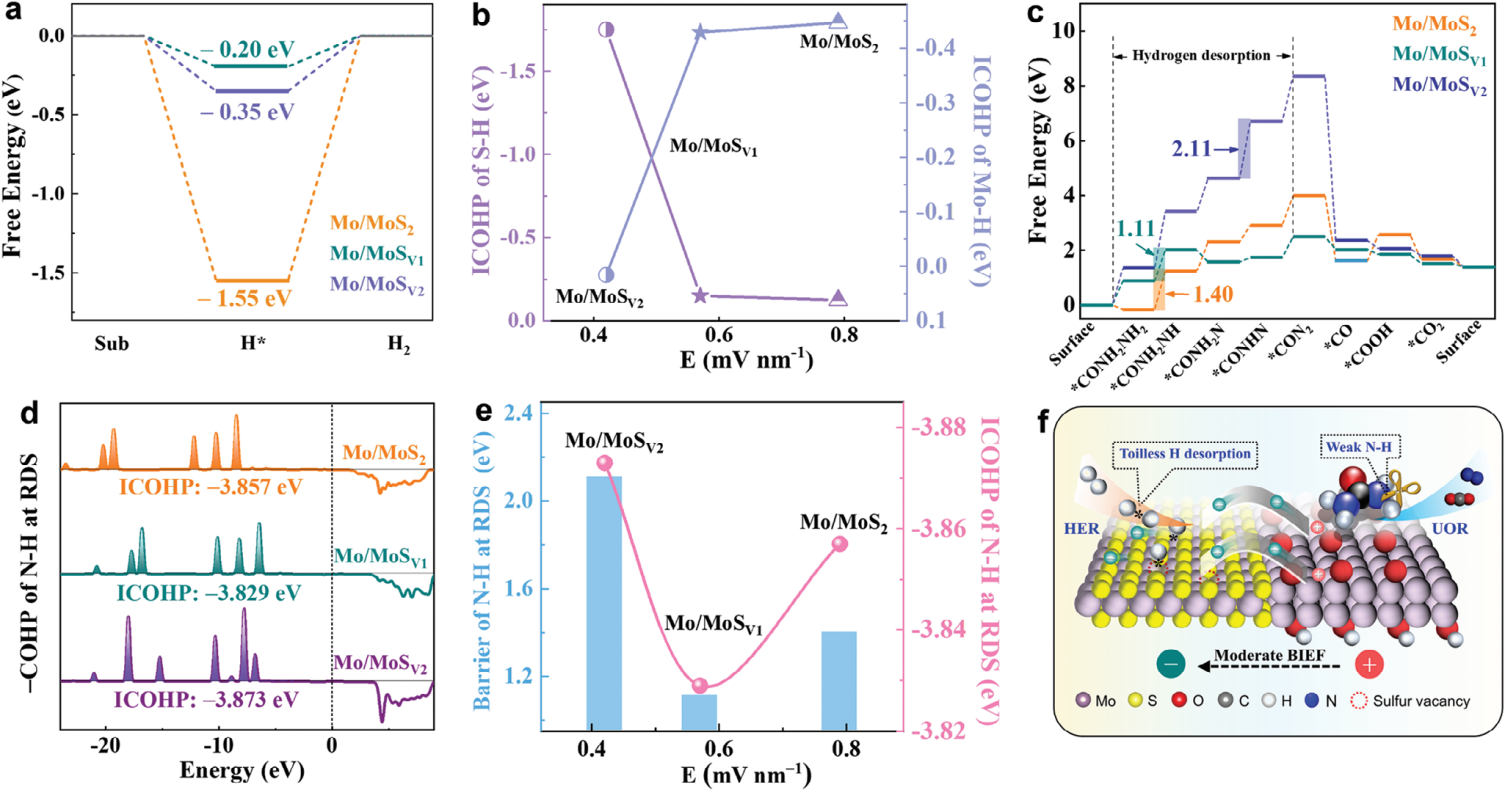
DFT calculations for Mo/MoS_Vn_. a) Gibbs free energy for the HER process. b) The calculated ICOHP of S‐H and Mo‐H after hydrogen adsorption. c) Gibbs free energies for the UOR process. d) Comparisons for the ICOHP of N─H bond at RDS. e) Relationships between energy barrier at RDS and the highest ICOHP of N‐H with BIEF intensity. f) Schematic illustration of enhancement mechanism.

DFT calculations were performed to dig out catalytic mechanisms of the Mo/MoS_Vn_ with different BIEFs. Different adsorption sites of H_2_O and urea molecules were first discussed (Figure S45, Supporting Information). The H_2_O adsorption energy at MoS_2_ surface was calculated to be −0.073 eV, which was ca. 4.3 times higher than that at Mo surface, indicating that HER could tend to happen at MoS_2_. By contrast, the urea adsorption energy at Mo surface was −1.65 eV, showing an improvement of ca.12‐folds versus that at MoS_2_ surface, suggesting that urea preferred to adsorb at Mo surface (Figure S46, Supporting Information). Considering the surface reconstruction (Figure 4d‐i), we built the catalytic models for the Mo/MoS_Vn_ and calculated corresponding Gibbs adsorption free energies for H^*^ (ΔG_H*_) (Figure S47, Supporting Information). Compared with the −1.55 eV of Mo/MoS_2_ and −0.35 eV of the Mo/MoS_V2_, the lowest ΔG_H*_ (−0.20 eV) of the Mo/MoS_V1_ implied the easiest hydrogen evolution (**Figure** **5**a). The partial densities of states (PDOS) were investigated to elucidate the different ΔG_H*_ of Mo/MoS_Vn_. As shown in Figures S48–S50 (Supporting Information), with the peak disappearance in the intrinsic H 1s, the PDOS of Mo 4d and S 2p after hydrogen adsorption delivered new and overlapped peaks with H 1s in the Mo/MoS_Vn_ (the dotted box in Figure S51 (Supporting Information), demonstrating the formation of S─H or Mo─H bonds. The PDOS results suggested that hydrogen adsorption in all the Mo/MoS_Vn_ were spontaneous, agreed with the negative values of all ΔG_H*_ values. In addition, with the decrease of ΔG_H*_, the overpotential and Tafel slope of the Mo/MoS_Vn_ nanoreactors gradually decreased (Figure S52, Supporting Information), and the Mo/MoS_V1_ with the lowest ΔG_H*_ exhibited best HER activity. The integrated crystal orbital Hamiltonian partition (ICOHP) values of S‐H and Mo‐H in the Mo/MoS_2_, Mo/MoS_V1_, and Mo/MoS_V2_ were calculated to be −1.75 and 0.016 eV, −0.151 and −0.429 eV, and −0.125 and −0.447 eV, respectively (Figures S53, S54, Supporting Information). Seen from the summary in Figure 5b, the bond energy of S‐H was gradually decreased accompanied by the increase of Mo‐H. Specifically, the S─H bond was the strongest in the Mo/MoS_2_, which was 10 times higher than those in the Mo/MoS_V1_ and Mo/MoS_V2_, consistent with the most difficult hydrogen desorption in the former. The Mo─H bond were dominant in the Mo/MoS_V1_ and Mo/MoS_V2_, and the lower ICOHP value of Mo‐H in the Mo/MoS_V1_ signified much easier hydrogen desorption, which matched with the less negative ΔG_H*_. Therefore, engineering of sulfur vacancy can optimize BIEF and combination of Mo/S‐H, and Mo/MoS_V1_ with moderate sulfur vacancy could better balance hydrogen adsorption and desorption process.

Figures S55–S57 (Supporting Information) show the energy barriers and adsorption–desorption structure models on the Mo surface of Mo/MoS_Vn_ in the UOR process, and the rate‐determining step (RDS) was the deprotonation of N‐H for all models (Figure 5c). Specifically, the RDS was the first hydrogen desorption step (^*^CON_2_H_4_ to ^*^CON_2_H_3_) with the energy barriers of 1.11 and 1.40 eV for the Mo/MoS_V1_ and Mo/MoS_2_, respectively. In contrast, the energy barrier was 2.11 eV for the Mo/MoS_V2_, which was determined by the third hydrogen desorption step (^*^CON_2_H_2_ to ^*^CON_2_H). The Mo/MoS_V1_ delivered the lowest barrier energy of deprotonation for the N‐H, implying the most facile UOR in the Mo/MoS_V1_. The ICOHP values of N─H bond in each hydrogen desorption step of the UOR process driven by the Mo/MoS_Vn_ are displayed in Figures S58–S61 (Supporting Information), which directly reflected the differences in each deprotonation step. According to the comparisons for ICOHP of N─H bond at RDS (Figure 5d), the highest ICOHP value of N─H bond in deprotonation steps for the Mo/MoS_V1_ was −3.829 eV (RDS: the first deprotonation step), which was lower than −3.857 eV (RDS: the first deprotonation step) for the Mo/MoS_2_ and −3.873 eV (RDS: the third deprotonation step) for the Mo/MoS_V2_. As the comparison in Figure S62 (Supporting Information), the Mo/MoS_V1_ with the lowest ICOHP value of N─H bond eexhibited most toilless urea oxidation. Combining with above analysis, the UOR with the lowest energy barrier and weakest N─H bonding strength at RDS could be only driven by the Mo/MoS_V1_ with moderate BIEF (Figure 5e).

The schematic illustration of enhancement mechanism for the HER||UOR system was presented in Figure 5f. Sulfur vacancy triggered the gradient modulation of BIEF, endowing the Mo/MoS_Vn_ with varying capacities for hydrogen adsorption on the MoS_Vn_ surface and urea adsorption on the Mo surface, which optimizes combination of Mo/S‐H in the HER and tailors the ICOHP of N‐H in the UOR. Accordingly, the Mo/MoS_V1_ with moderate BIEF exhibits the weakest Mo‐H and N─H bonding strength, evidently facilitating hydrogen desorption from Mo site on the MoS_V1_ and hydrogen desorption from N‐H at RDS on the Mo. As a result, the hydrogen evolution and urea electrooxidation process are highly promoted. Just as the electrocatalytic results displayed in Figure 4c, the HER and UOR activities of the Mo/MoS_V1_ were much higher than those of Mo/MoS_V2_ with weak BIEF and Mo/MoS_2_ with strong BIEF.

### 2.6 HER||UOR System and LCA for Mo/MoS_V1_

**Figure 6:**
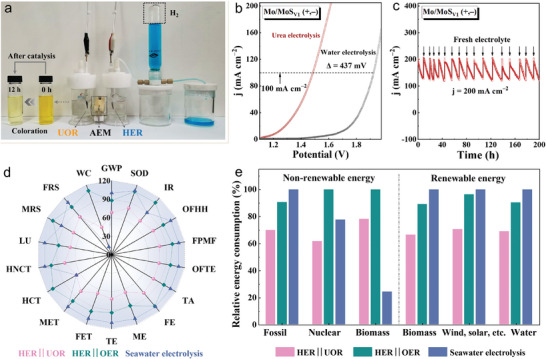
Hydrogen production electrolyzer and LCA for the Mo/MoS_V1_. a) Photograph of the HER||UOR. b) Comparisons of LSV curves in the HER||OER and HER||UOR with 90% IR compensation. c) The stability test of HER||UOR at 200 mA cm^−2^ for 200 h. d) LCAs of different hydrogen production systems. e) Relative energy consumptions in different hydrogen production systems.

We fabricated HER||UOR system using the Mo/MoS_V1_ nanoreactor as the anode and cathode simultaneously to evaluate two‐electrode performances in simulated urea wastewater (1 M KOH + 0.33 M urea). **Figure** **6**a shows the photograph of H‐type electrolyzer with anion exchange membrane (AEM). The hydrogen evolution on the cathode was collected and measured by drainage method, and the urea oxidation on the anode was investigated by coloration reaction. The entire process of hydrogen collection was recorded in Supplementary Movie. Moreover, HER||OER system without urea was also constructed for comparison. As depicted in Figure 6b, the voltage required for urea electrolysis was 1.49 V to deliver 100 mA cm^−2^, which was 437 mV lower than that of traditional water electrolysis, validating the UOR could replace OER to reduce cell voltage and achieve energy‐saving hydrogen evolution. Seen from the LSV curves (Figures S63, S64, Supporting Information), the urea‐assisted water splitting voltage at 100 mA cm^−2^ for the Mo/MoS_V1_ (+,−) cell was also lower than those of the commercial Pt/C (−)||RuO_2_ (+), Mo (+,−), and MoS_2_ (+,−) cells. Furthermore, the Mo/MoS_V1_ also displayed outstanding catalytic activities at both low and high current densities compared with the most recently reported bifunctional catalysts in urea‐assisted electrolysis (Figure S65, and Table S5, Supporting Information). Seen from the comparisons of chronoamperometry tests (Figure S66, Supporting Information), the Mo/MoS_V1_ (+,–) cell demonstrated a negligible activity attenuation comparing to the Mo (+,–) and MoS_2_ (+,–) cells at 10 mA cm^−2^. Meanwhile, the ideal catalytic durability for the Mo/MoS_V1_ (+,–) electrolyzer at 200 mA cm^−2^ was further confirmed via adding fresh electrolyte every 12 h and the property's instant return to the initial level (Figure 6c). According to the FE and rate of hydrogen production for the Mo/MoS_V1_ (+,–) cell (Figure S67, Supporting Information), the FE was stable over 90% at different voltages and even reached 99.52% at 1.48 V. Figure S68 (Supporting Information) displayed the ultraviolet spectroscopy of the electrolyte with a strong absorbance peak at 460 nm assigned to the urea. The intensity of this peak gradually weakened from 0 to 12 h. The digital photos of solutions after different degradation times were presented in Figure S69 (Supporting Information), where we could find that the urea‐rich solution notably changed in color from deep yellow to nearly colorless after 12 h. We further conducted three replicate experiments on the coloration reaction (Figure S70, Supporting Information), and the average degradation rate was calculated to be 90.06%. The results verified the great potential of the Mo/MoS_V1_ as a bifunctional catalyst for stable hydrogen evolution and effective purification of urea‐rich wastewater.

We performed a comprehensive environmental impact and sustainable ability evaluation via using the methodology of LCA from “gate to gate” for three main hydrogen production technologies including the HER||UOR system, HER||OER system, and seawater electrolysis systems considering the material, energy consumption, and waste emissions when producing 1 kg hydrogen. The HER||UOR and HER||OER systems were constructed utilizing the Mo/MoS_V1_ as bifunctional catalyst, and the data of the seawater electrolysis were extracted from the literature.^[^ ^33^ ^]^ Eighteen midpoint environmental impact categories were quantitatively analyzed and shown in Table S6 (Supporting Information), including global warming potential (GWP), stratospheric ozone depletion (SOD), ionizing radiation (IR), ozone formation human health (OFHH), fine particulate matter formation (FPMF), ozone formation terrestrial ecosystems (OFTE), terrestrial acidification (TA), freshwater eutrophication (FE), marine eutrophication (ME), terrestrial ecotoxicity (TE), freshwater ecotoxicity (FET), marine ecotoxicity (MET), human carcinogenic toxicity (HCT), human non‐carcinogenic toxicity (HNCT), land use (LU), mineral resource scarcity (MRS), fossil resource scarcity (FRS), and water consumption (WC). By contrast, the HER||UOR system's performance across various environmental categories was notably superior, demonstrating a reduced impact on multiple fronts including acidification, eutrophication, ecotoxicity, etc. (Figure 6d). Specifically, the GWP closely related to CO_2_ emission of the HER||UOR system lowered by 19.43% and 31.70%, respectively, compared with those of HER||OER system and seawater electrolysis (Tables S7–S9, Supporting Information). Both the SOD and FE of the HER||UOR system were reduced by ca. 30% compared to those of the other two systems. Taking the seawater electrolysis as 100%, the HER||UOR system only contributed to 67.98% and 68.66% in TA and FPMF, respectively, which were also much lower than the HER||OER system (87.35%). In addition, the HCT, MRS, and WC of the HER||UOR system accounted for ca. 1/2, 3/5, and 2/5 the amounts of HER||OER system, respectively. Furthermore, the detailed energy consumption values validated the energy efficiency and sustainability of the three systems for hydrogen production (Figure 6e; Table S10, Supporting Information). For the non‐renewable resources, our analysis revealed that the HER||UOR system reduced the reliance on fossil fuels, resulting in a lower overall carbon footprint, which is crucial for mitigating climate change. Besides, the consumptions of nuclear energy and biomass were also comparatively low, further highlighting the advantages in improving energy efficiency for the HER||UOR system. For the renewable resources, the HER||UOR system also exhibited the lowest consumption and highest utilization efficiency of renewable energies like biomass, wind, water, solar energy, etc, significantly reducing the environmental footprint associated with energy extraction and use. The environmental impact contributions of raw materials and energies used in the three kinds of hydrogen production system to yield 1 kg hydrogen were further analyzed based on the data in Tables S7–S9 (Supporting Information) and concluded in Figures S71–S73 (Supporting Information). Obviously, the electricity consumption was the main part of environmental impact for the three systems, and the HER||UOR system presented the lowest electricity consumption. Additionally, the HER||UOR system's optimized energy use resulted in a more stable and predictable performance, which is advantageous for scaling up and integration into existing infrastructure. Notably, although a high CO_2_ emission was needed, the value could be lowered to 4.4 kg CO_2_ eq kg H_2_^−1^ when replacing electricity source with renewable wind or solar photovoltaic electricity, which was close to median production emission.^[^ ^1^ ^]^ Therefore, the simulated urea wastewater system assembled with the Mo/MoS_V1_ nanoreactor could not only provide a more sustainable method for hydrogen production but also offer significant environmental benefits by utilizing waste materials and renewable energy sources. The robustness of these results was further confirmed by additional sensitivity analyses using Monte Carlo simulation for 1000 times (Tables S11–S13, Supporting Information), which demonstrated consistent performance across various scenarios and assumptions, reinforcing the reliability of the HER||UOR system's environmental and economic advantages.

## 3 Conclusion 

In summary, we report the hollow Mo/MoS_Vn_ nanoreactors with tunable BIEFs as efficient bifunctional electrocatalysts for energy‐saving and eco‐friendly hydrogen production. Specifically, the Mo/MoS_2_, Mo/MoS_V1_, and Mo/MoS_V2_ nanoreactors with various sulfur vacancies are precisely developed through treating MoS_2‐x_O_x_ precursors with pre‐encapsulated (CTAB)_y_S_2_ via one‐step anion exchange and reduction reaction. The BIEF intensities in the hollow nanoreactors are gradiently modulated because of the charge transfer differences triggered by different concentration of sulfur vacancies. Interestingly, the performances of the Mo/MoS_Vn_ nanoreactors toward HER and UOR exhibit parabola‐shaped relationship with the BIEF intensity. As a result, the Mo/MoS_V1_ nanoreactor with moderate BIEF intensity presents much better bifunctional activity and stability than the Mo/MoS_2_ and Mo/MoS_V2_ analogues. Moreover, the Mo/MoS_V1_ (+,–) urea‐assisted water electrolyzer delivers much lower cell voltage than that of traditional water electrolysis, and also demonstrates negligible activity attenuation at a high current density. According to DFT calculations, the moderate BIEF in the Mo/MoS_V1_ not only accelerates the hydrogen adsorption and desorption on the MoS_V1_ surface leading to more facile hydrogen evolution, but also weakens N─H bond strength on the Mo surface promoting urea electrooxidation process. LCA verifies that the HER||UOR system assembled with Mo/MoS_V1_ nanoreactor is notably superior across eighteen environmental categories compared with those of HER||OER system and seawater electrolysis. In addition, the HER||UOR system shows lowered overall carbon footprint and reduced environmental footprint associated with energy extraction and use. Our finding involves the precise modulation of BIEF via vacancy engineering in the nanoreactors, and may shed light on the design of efficient bifunctional materials for sustainable water electrosynthesis.

## 4 Experimental Section

Experimental details were provided in the Supporting Information.

## Conflict of Interest

The authors declare no conflict of interest.

## Supporting information



Supporting Information

Supplemental Movie

## Data Availability

Research data are not shared.
