# Supplementary material for: Hollow Mo/MoSVn Nanoreactors with Tunable Built‐in Electric Fields for Sustainable Hydrogen Production
Source: Adv Mater. 2024 Dec 8;37(5):2415269. doi: 10.1002/adma.202415269 (PMC11795732; doi:10.1002/adma.202415269)
Supplement: Supplementary file 1 — Supporting Information [file ADMA-37-2415269-s001.docx]

Supporting Information

**Hollow Mo/MoS_Vn_ Nanoreactors with Tunable Built-in Electric Fields for Sustainable Hydrogen Production**

*Feilong Gong,^*^ Zhilin Chen, Chaoqun Chang, Min Song, Yang Zhao, Haitao Li, Lihua Gong, Yali Zhang,* *Jie Zhang, Yonghui Zhang, Shizhong Wei,^*^ and Jian Liu**^*^*

**Contents:**

Supplementary Notes 1-8

Supplementary Figures 1-73

Supplementary Tables 1-13

Supplementary References

**Supplementary Notes**

**Note S1. Synthesis of the precursors and** **hollow Mo/MoS_Vn_ nanoreactors**

The preparation process involved a modified mimicked embryo formation technology developed in our lab. Typically, Na_2_MoO_4_ (S1: 1 mmol Na_2_MoO_4_·2H_2_O and 0.6 g glucose in 300 mL H_2_O) aqueous solution was added into the oil solution (S2: 15 mmol CTAB in 100 mL n-butanol) under stirring. Ethylene glycol (50 mL) and hydrochloric acid (0.4 mL) were then introduced and stirred for 0.5 h. Finally, thiourea (20 mmol) was added into above solution and further stirred for 1 h at room temperature. The milky white mixture was subsequently transferred into a high-pressure hydrothermal autoclave (500 mL) with PTFE liner and kept at 220 °C for 8 h. The precipitate was separated by filtration and washed with distilled water and absolute ethanol, and dried at 80 °C for 2 h to obtain black powder, named as Precursor-20. The Precursor-20 was treated at 900 °C in Ar/H_2_ for 4 h with a raise rate of 5 °C min^−1^ to obtain the Mo/MoS_V1._

Keeping the basic reaction parameters constant, the Precursor-10 and Precursor-40 were subsequently synthesized via changing the amount of thiourea as 10 and 40 mmol. Correspondingly, the above precursors were annealed at 900 °C in Ar/H_2_ for 4 h with a raise rate of 5 °C min^−1^ to obtain the Mo/MoS_V2_ and Mo/MoS_2_.

**Note S2. Synthesis of hollow MoS_2_ microspheres**

The Precursor-40 was annealed at 900 °C in Ar for 4 h with a raise rate of 5 °C min^−1^ to produce the MoS_2_.

**Note S3. Synthesis of MoO_2_ and hollow Mo** **microspheres**

The synthesis process of MoO_2_ was similar with the Precursor-20 except for removing the thiourea. The obtained MoO_2_ was annealed at 900 °C in Ar/H_2_ for 4 h with a raise rate of 5 °C min^−1^ to prepare the hollow Mo microspheres.

**Note S4. Characterizations**

X-ray diffraction (XRD) was performed on a D/max 2550 V X-ray diffractometer (Rigaku, Tokyo, Japan) from 5 to 80 ° with mono-chromatized Cu K*_α_* (λ = 0.15406 nm, scanning rate of 0.01° s^-1^). The field emission scanning electron microscopy (FESEM) was completed on a JSM-7001F. The transmission electron microscopy (TEM) was carried out on a JEM-2100 operated at 200 kV. High-angle annular dark-field (HAADF) and corresponding EDS mapping analyses were performed on a FTEM/STEM (JEM-ARM200F, Japan) at an acceleration voltage of 200 kV with a spherical aberration corrector. Raman spectra analysis was performed on an Acton Spectra Pro500i Raman spectrometer with a 532 nm excitation wavelength and a power of 5 mW (2 % of the maximum power). X-ray photoelectron spectra (XPS) were acquired on a Thermo Scientific Escalab 250Xi (ESCALAB 250Xi) with monochromatized Al-Kα X-ray as the excitation source. In situ Raman system was built through the combination of the electrochemical workstation (CHI600E, Shanghai), the ESCALAB 250Xi, and the three-electrode pool (C031-1, Gaoss Union). X-ray absorption fine structure (XAFS) data of the Mo K-edge were performed at ANSTO, New South Wales, and Australia. Electron Paramagnetic Resonance (EPR) data was collected using a JES-FA200 (JEOL, Japan). Nitrogen absorption-desorption tests were performed on a Micromeritics ASAP 2460 adsorption apparatus. The Langmuir and Brunauer-Emmett-Teller (BET) surface areas, pore volume and pore size were obtained by analyzing the N_2_ adsorption-desorption isotherms with Micromeritics ASAP 2020 built-in software. In FT-IR spectra were taken on a Bruker tensor II spectrometer (4000-400 cm^-1^) to monitor the evolution of functional groups during HER and UOR. The optical absorption of samples prepared are characterized by UV–vis spectrometer (TU-1901). The surface potential of the catalyst was measured by using Zeta potential analyzer (DLS) and Atomic Force Microscope.

**Note S5. Fabrication and measurement of the electrodes**

All electrochemical measurements were carried out at CHI660E electrochemical workstation (Shanghai Chenhua Company) at room temperature. A working electrode was made by casting 250 μL (2 mg mL^–1^) as-prepared catalysts to a Ni foam electrode (1×1 cm^2^). The reference electrode is the Hg/HgO electrode and the counter electrode is the graphite rod. All polarization curves for HER and HER||OER were measured in 1 M KOH (pH = 14) solution at a scan rate of 5 mV s^–1^. All polarization curves for UOR were measured in 1 M KOH + 0.33 M urea solution at a scan rate of 5 mV s^–1^. HER and UOR data are converted to reversible hydrogen electrode (RHE) according to the equation (1):

$E_{\mathrm{VS}.RHE}=E_{\mathrm{VS}.\mathrm{Hg}/\mathrm{HgO}}＋ 0.591 + 0.098$ (1)

The H-type electrolyzer was assembled with an anode (1.0 cm^2^), cathode (1.0 cm^2^), and anion exchange membrane (AEM, FAA-3-PK-130, Suzhou Sinero Technology Co., Ltd). Mo/MoS_V1_ catalysts coated on NF were used as the cathode and anode, and the loading mass of every catalyst was approximately 2 mg cm^–2^. For comparison, commercial Pt/C (20 wt.%) and RuO_2_ electrocatalysts coated on the pressed NF (1×1 cm^2^) were used as cathode and anode, respectively, to construct the Pt/C (–)||RuO_2_ (+) electrolyzer. We used 1 M KOH + 0.33 M urea as the electrolyte to simulate the urea wastewater because the urea content in the industrial wastewater was *ca.* 0.33 mol/L. The cathode was the HER process for producing hydrogen, and the anode was the UOR process for urea degradation. All polarization curves for HER||UOR were measured at a scan rate of 5 mV s^–1^. The hydrogen generated by the experiment was collected by the drainage method. The theoretical values were calculated according to the equation (2):

$n_{H_{2}}=\frac{\mathrm{It}}{\mathrm{nF}}$ (2)

where n(H_2_) is the amount of H_2_ gas (mol), I is the measured current (A), t is the water splitting time (s), n represents the number of electron transferred in HER process (n = 2), and F is the Faraday constant (96485.4 C mol^−1^). The FE was concluded by the ratio of experimental gas amount and theoretical value, as exemplified by equation (3):

$\mathrm{FE}=\frac{n(experimental H_{2})}{n(theoretical H_{2})}$ (3)

Double layer capacitance (*C*_dl_) is measured by linear fitting of cyclic voltammetry (CV) curves at different scanning rates, and the polarization current is half of the sum of the oxidation current and the reduction current.

The electrochemical active surface area (ECSA) can be estimated using the C_dl_. The specific capacitance for a flat surface (C_s_) is supposed to be 40 μF cm^–2^, and the ECSA is estimated by the following formula:

$ECSA=\frac{C_{dl}}{C_{s}}$ (4)

**Note S6. Measurement of urea elimination**

The urea degradation rate in alkaline UOR was measured by a modified diacetyl monoxime Antipyrine method (GB/T 18204.2-2014), and the reaction mechanism is as follows: the urea reacted with diacetyl monoxime and antipyrine under the condition of strong acid as well as heating, which shows yellow color and has the maximum absorption peak at 460 nm.

Then, an overall urea electrolyzer using 1 cm^2^ area of Mo/MoS_V1_ as both of the cathode and anode was constructed and employed in 40 mL alkaline solution (1 M KOH + 0.33 M urea) for 12 hours. About 0.5 mL reacted urea rich alkaline solution was collected after every test for 1 hour. The relationship between urea concentration and absorbance in this range conforms to Lambert-Beer law, by which the degradation rate can be calculated as follows:

$\eta=(1-\frac{A_{T}}{A_{0}}) \times100\%$ (5)

where η is urea degradation rate, A_T_ is the absorbance of urea concentration at time T (0 ≤ T ≤ 12), A_0_ is the absorbance of urea concentration at 0 hour.

**Note S7. Density functional theory (DFT) calculations**

First principles calculations were performed based on DFT by Vienna Ab initio Simulation Package (VASP). The projector augmented wave (PAW) method with Perdew-Burke-Ernzerhof (PBE) pseudopotentials was utilized to treat the electron-ion interaction. The plan wave basis set as 400 eV. The total energy convergence criterion of electronic self-consistent field (SCF) was carried out 10^-5^ eV while the structure optimizations were relaxed until the force was less than 0.05 eV/Å. Then the spin polarization was adopted in. The Brillouin zone in the calculation was sampled by a 3×3×1 k points mesh. The catalyst models of Mo (110) and MoS_2_ (001) were constructed by the 2×2×1 and 3×3×1 supercell. The Mo/MoS_vn_ models were constructed by the Mo (110) and MoS_2_ (001) with different sulfur vacancy using left-right pattern to simulate the interfacial interaction. The Mo/MoS_V1_ represented one sulfur vacancy and the Mo/MoS_V2_ signified two sulfur vacancies in the MoS_2_. The vacuum space along the Z direction was 15 Å to prevent the effect of the adjacent atomic slabs.

In order to compare the activity of hydrogen evolution, the structures of Mo/MoS_2_, Mo/MoS_V1_, Mo/MoS_V2_, and their hydrogen adsorption models (Figure S48, Supporting Information) were built to calculate the Gibbs free energy. HER process in the alkaline electrolyte involves the following steps:

$*+H_{2}O+e^{-}\to H^{*}+OH^{-}$ (Volmer step) (6)

$2H^{*}\to H_{2}\uparrow$ (Tafel step) (7)

$H^{*}+H_{2}O+e^{-}\to OH^{-}+H_{2}\uparrow$ (Heyrovsky step) (8)

where * represents the adsorption site. The reaction free energy is calculated by the following equation:

$\boldsymbol{\Delta}G\boldsymbol{=\Delta}E\boldsymbol{+\Delta}\mathrm{ZPE}\mathbf{-}T\boldsymbol{\Delta}S$ (9)

where ∆E is the change of the adsorption energy on the substrates, ∆ZPE and ∆S are the changes of zero-point energy and entropy correction (T was set to be 298.15 K). ZPE could be derived after frequency calculation by equation as follows:

$ZPE=\frac{1}{2}\sum hv_{i}$ (10)

where $h$ is the Planck constant, $v_{i}$ are the computed vibration frequencies. And the TS values of adsorbed species are calculated with the vibration frequencies, as shown in equation:

$TS_{v}=k_{B}T[\sum_{K} \ln\left( \frac{1}{1-e^{-\frac{hv}{k_{B}T}}} \right)+\sum_{K} \frac{hv}{k_{B}T}\frac{1}{\left( e^{\frac{hv}{k_{B}T}}-1 \right)}+1]$ (11)

The value of each $\boldsymbol{\Delta}\mathrm{ZPE}\mathbf{-}T\boldsymbol{\Delta}S$ was set as the thermal correction to $\boldsymbol{\Delta}G$ which was calculated by the VASPKIT [VASPKIT: A user-friendly interface facilitating high-throughput computing and analysis using VASP code]. The urea oxidation reaction produces nitrogen and carbon dioxide by following the reactions below:

$*+CON_{2}H_{4}\to*CON_{2}H_{4}$ (12)

$*CON_{2}H_{4}+OH^{-}\to*CON_{2}H_{3}+H_{2}O+e^{-}$ (13)

$*CON_{2}H_{3}+OH^{-}\to*CON_{2}H_{2}+H_{2}O+e^{-}$ (14)

$*CON_{2}H_{2}+OH^{-}\to*CON_{2}H+H_{2}O+e^{-}$ (15)

$*CON_{2}H+OH^{-}\to*CON_{2}+H_{2}O+e^{-}$ (16)

$*CON_{2}\to*CO+N_{2}$ (17)

$*CO+OH^{-}\to*COOH+e^{-}$ (18)

$*COOH+OH^{-}\to*CO_{2}+H_{2}O+e^{-}$ (19)

$*CO_{2}\to*+CO_{2}$ (20)

**Note S8. Life cycle assessment (LCA)**

Life cycle environment impacts were modeled using SimaPro 9.4 with Ecoinvent 3, ELCD, and USLCI inventories. As one of the most applied methods worldwide, ReCiPe 2016 Midpoint (H) V1.07 / World (2010) H was selected to evaluate the environmental impact of hydrogen production for the HER||UOR system, HER||OER system, and seawater electrolysis to produce 1 kg hydrogen. Eighteen environmental impact categories were quantitatively analyzed, containing global warming potential (GWP, in kg CO_2_ eq), stratospheric ozone depletion (SOD, in kg CFC11 eq), ionizing radiation (IR, in kBq Co-60 eq), ozone formation human health (OFHH, in kg NO_x_ eq), fine particulate matter formation (FPMF, in kg PM_2.5_ eq), ozone formation terrestrial ecosystems (OFTE, in kg NO_x_ eq), terrestrial acidification (TA, in kg SO_2_ eq), freshwater eutrophication (FE, in kg P eq), marine eutrophication (ME, in kg N eq), terrestrial ecotoxicity (TE, in kg 1,4-DCB eq), freshwater ecotoxicity (FET, in kg 1,4-DCB eq), marine ecotoxicity (MET, in kg 1, 4-DCB eq), human carcinogenic toxicity (HCT, in kg 1,4-DB eq), human non-carcinogenic toxicity (HNCT, in kg 1, 4-DCB eq), land use (LU, in m^2^a crop eq), mineral resource scarcity (MRS, in kg Cu eq), fossil resource scarcity (FRS, in kg oil eq), and water consumption (WC, in m^3^).

Further, Cumulative Energy Demand V1.11 / Cumulative energy demand (CED) was applied to evaluate six impact categories of energy consumption including both fossil and renewable sources, such as non-renewable fossil, non-renewable nuclear energy, non-renewable biomass, renewable biomass, renewable wind energy, solar energy, geothermal energy, and renewable water energy. In order to calculate the total value of cumulative energy demand, every impact category was assigned with the weight factor of 1.

In order to evaluate the results accuracy of three hydrogen production processes, the method of Monte Carlo simulation was used to investigate the uncertainty analysis of the characterization results for each process. The uncertainty analysis results were obtained via conducting the Monte Carlo simulation for 1000 times, and the variable coefficient (VC) represented the index which could reflect the difference degree or dispersion degree of each flag value in the whole population.

**Supplementary Figures**

**
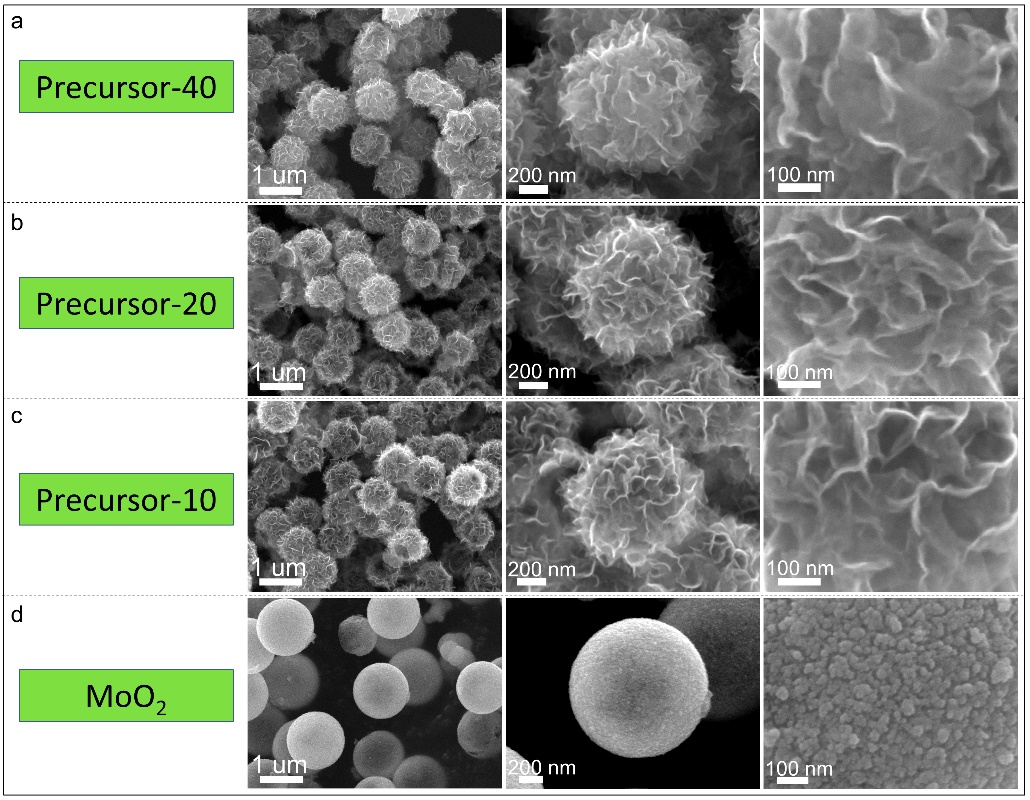
**

**Figure S1.** The FESEM images of the MoO_2_, Precursor-10, Precursor-20, and Precursor-40 at different magnifications.


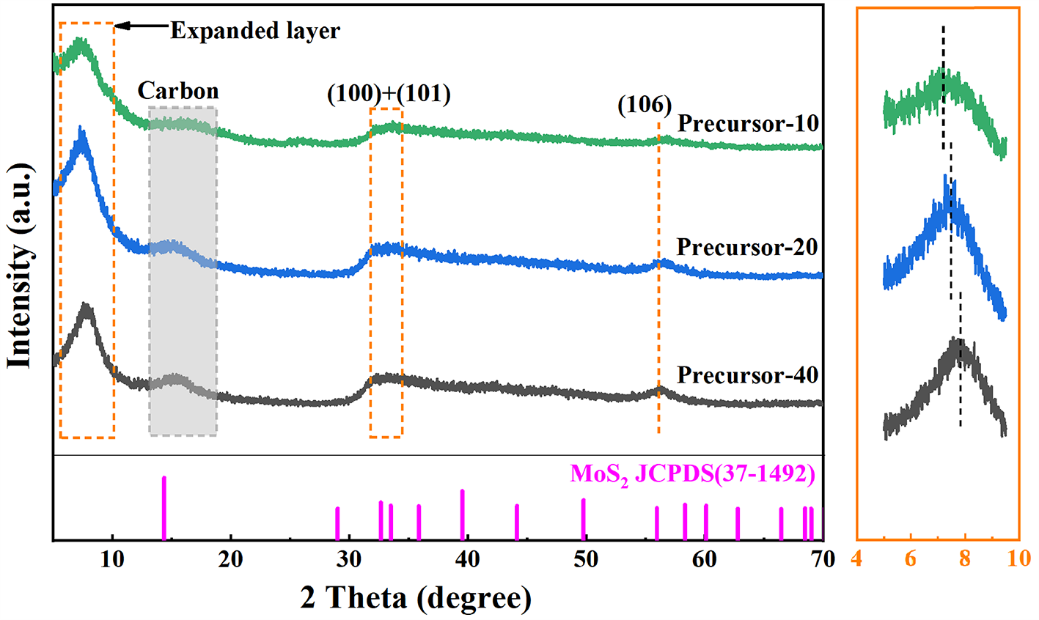


**Figure S2.** The XRD patterns of the Precursor-10, Precursor-20, and Precursor-40.


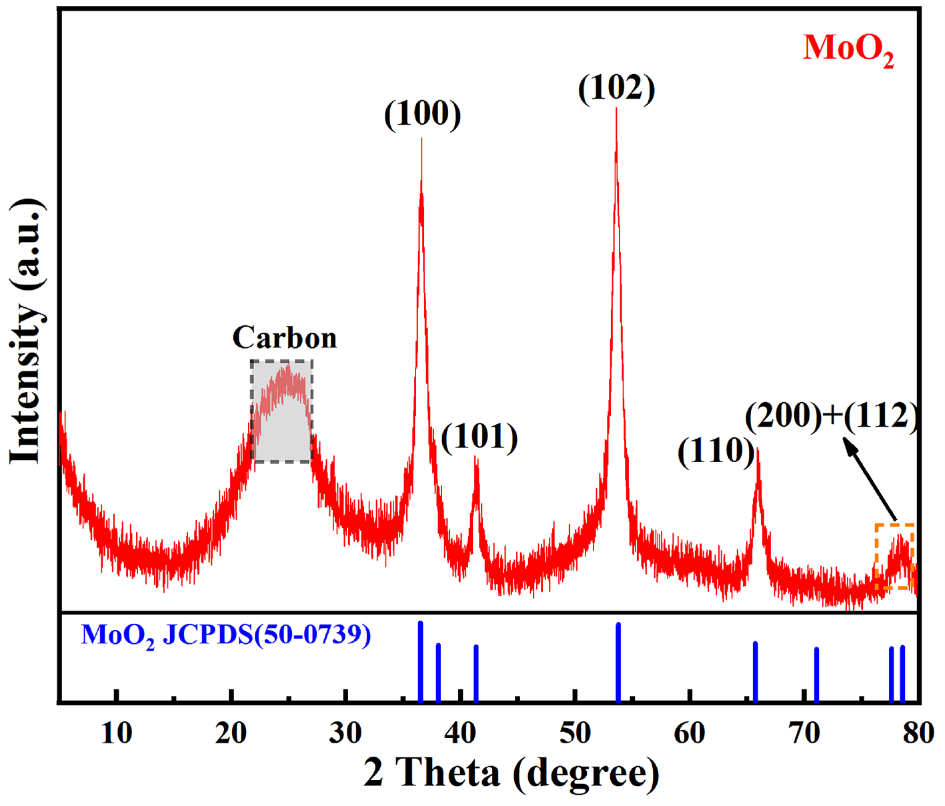


**Figure S3.** The XRD profile of the MoO_2_.


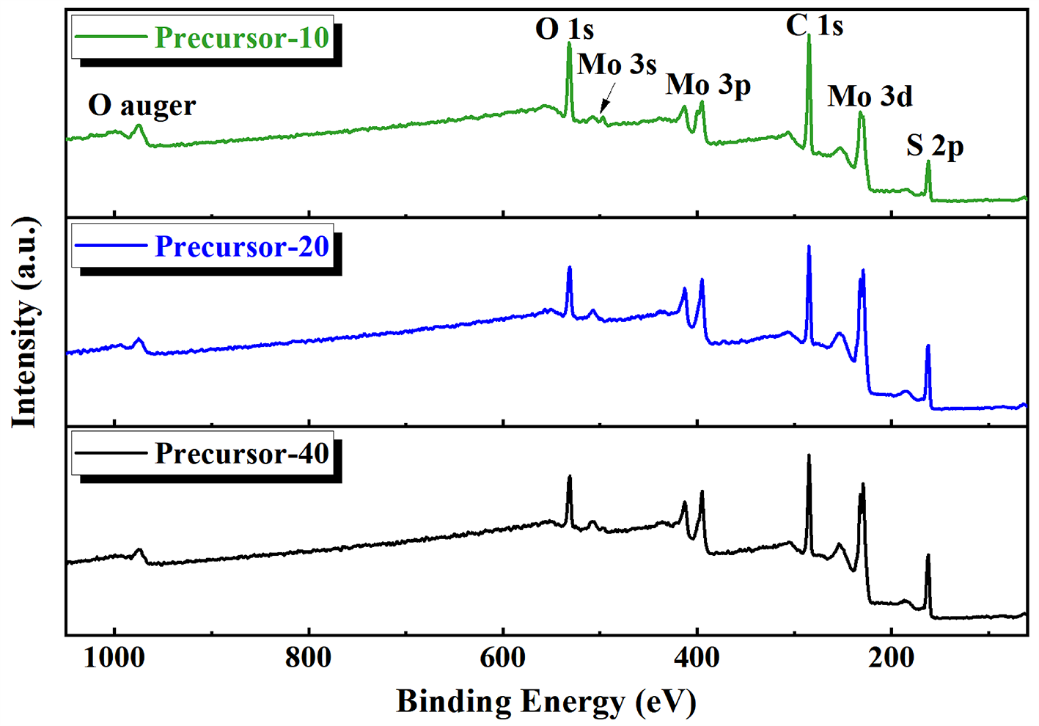


**Figure S4.** The comparisons of survey XPS spectra for the Precursor-10, Precursor-20, and Precursor-40.


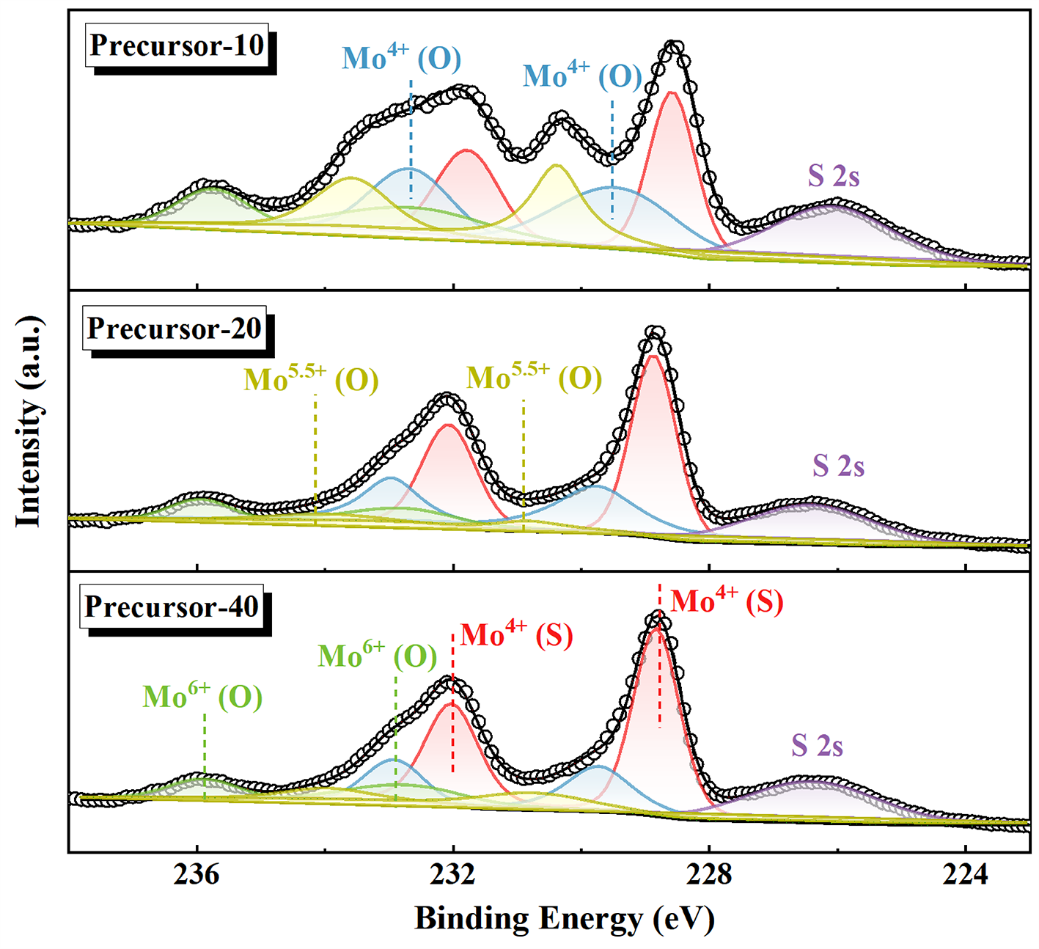


**Figure S5.** The comparisons of high-resolution XPS spectra of Mo 3d for the Precursor-10, Precursor-20, and Precursor-40.


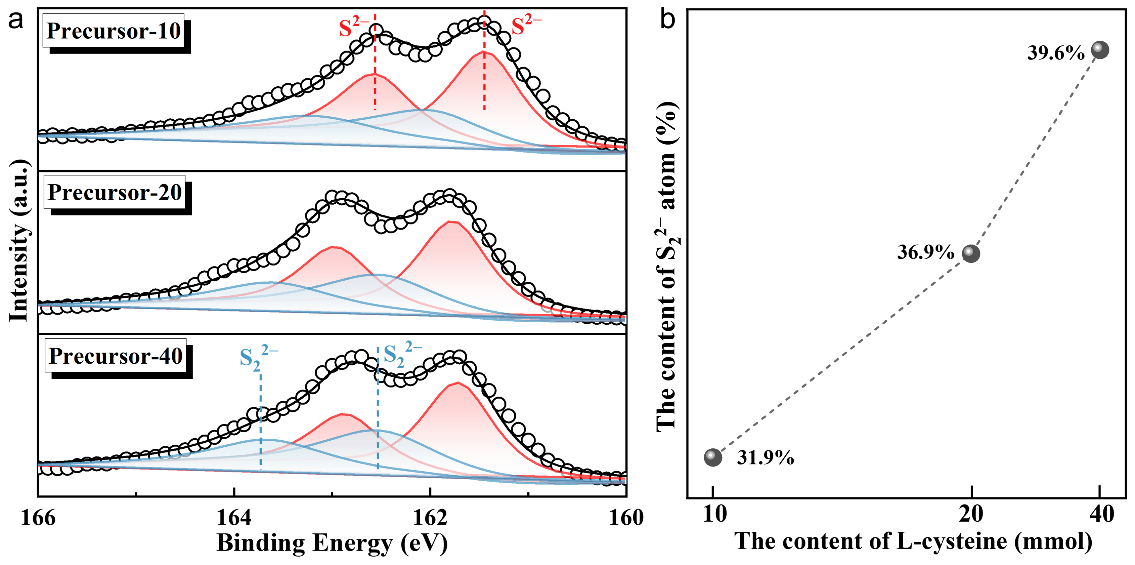


**Figure S6.** a) The comparisons of high-resolution XPS spectra of S 2p for the Precursor-10, Precursor-20, and Precursor-40. b) The percentages of S_2_^2−^ in the Precursor-10, Precursor-20, and Precursor-40.


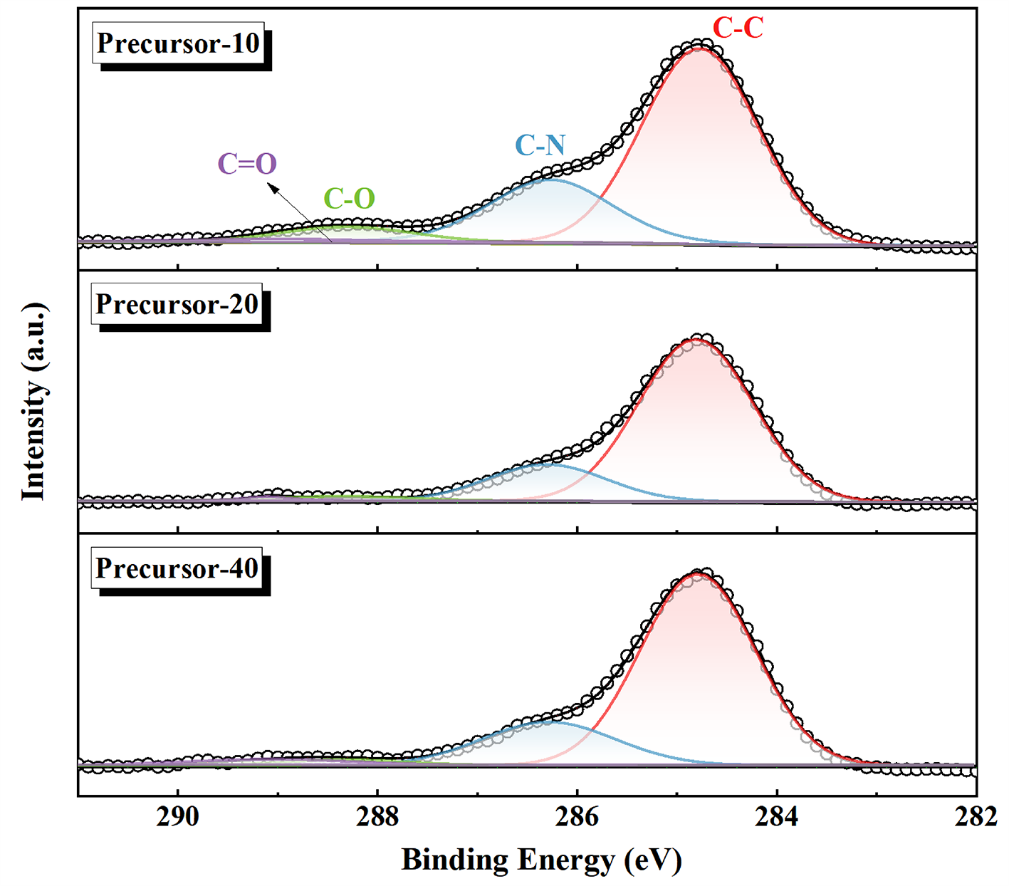


**Figure S7.** The comparisons of high-resolution XPS spectra of C 1s for the Precursor-10, Precursor-20, and Precursor-40.


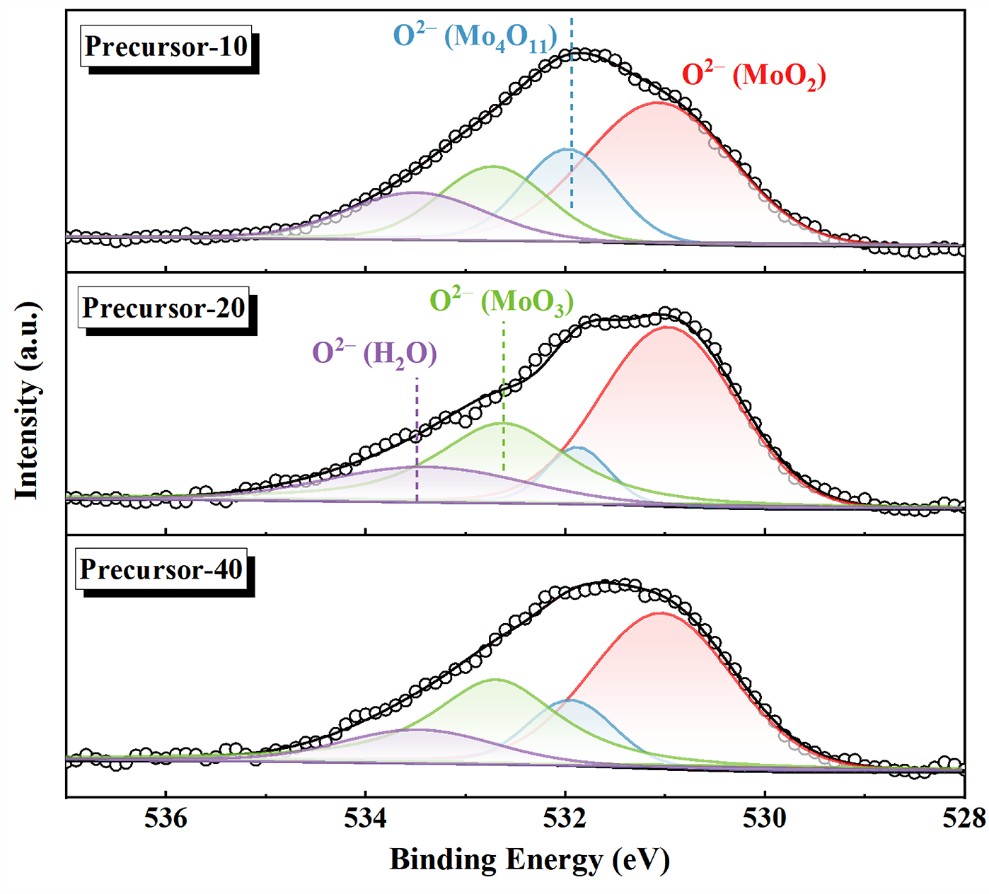


**Figure S8.** The comparisons of high-resolution XPS spectra of O 2p for the Precursor-10, Precursor-20, and Precursor-40.


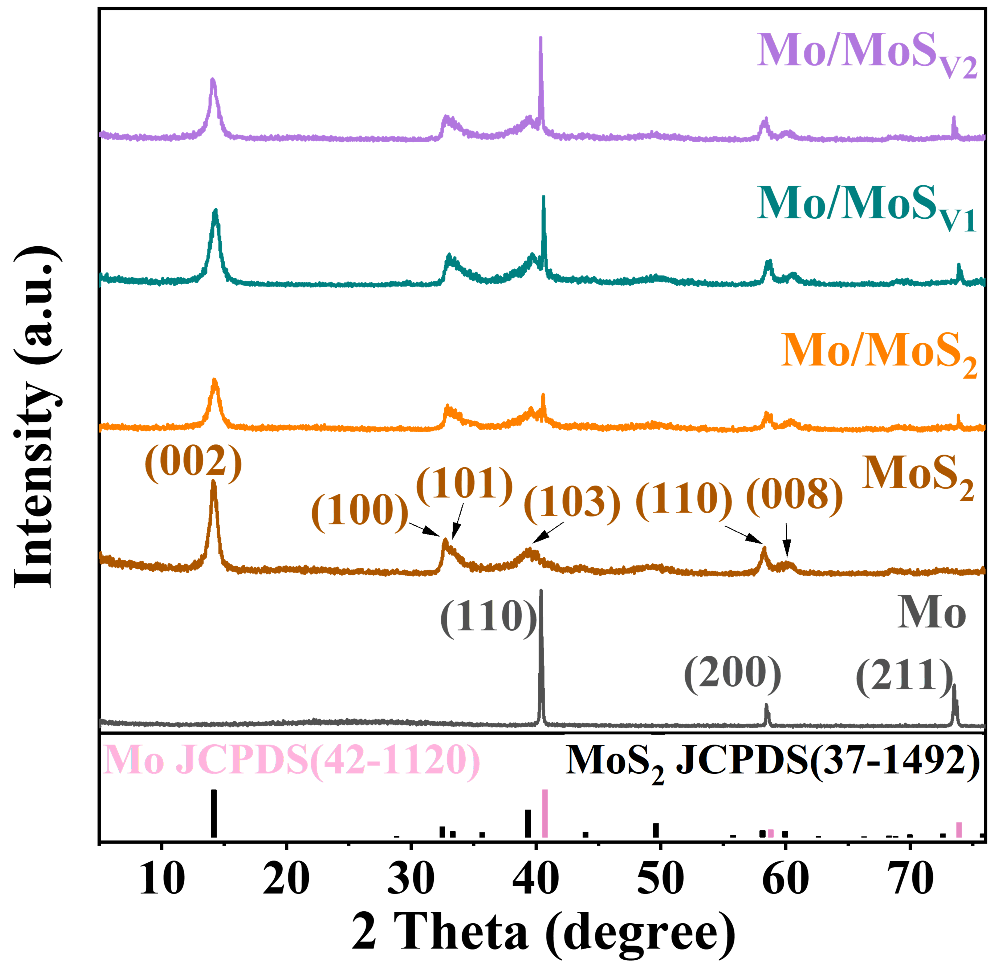


**Figure S9.** The comparisons of XRD patterns for the Mo, MoS_2_, Mo/MoS_2_, Mo/MoS_V1_, and Mo/MoS_V2_.


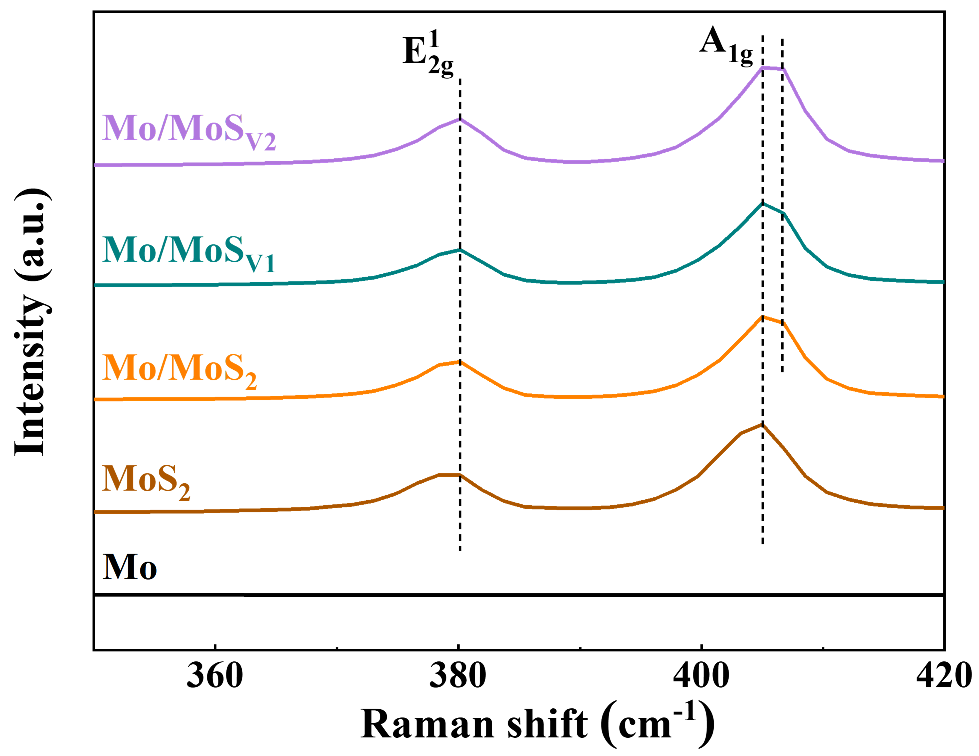


**Figure S10.** The characterizations of Raman spectra for the Mo, MoS_2_, Mo/MoS_2_, Mo/MoS_V1_, and Mo/MoS_V2_.


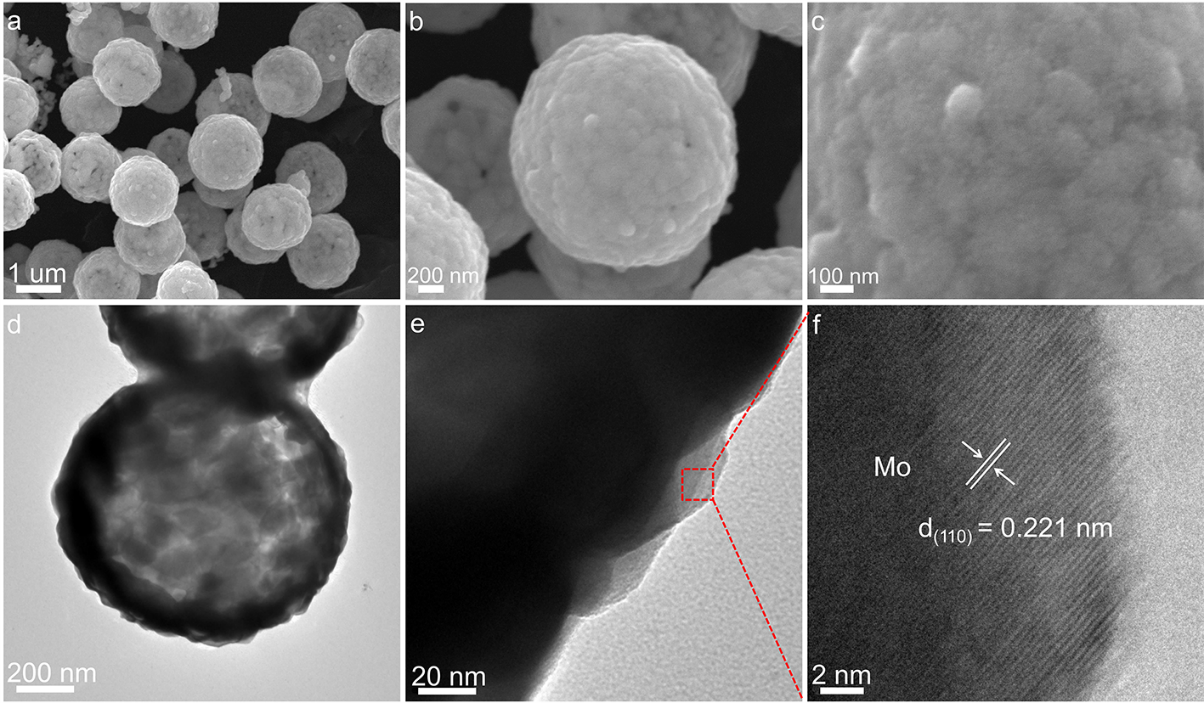


**Figure S11.** a-c) FESEM, d,e) TEM, and f) HRTEM images of the hollow Mo.


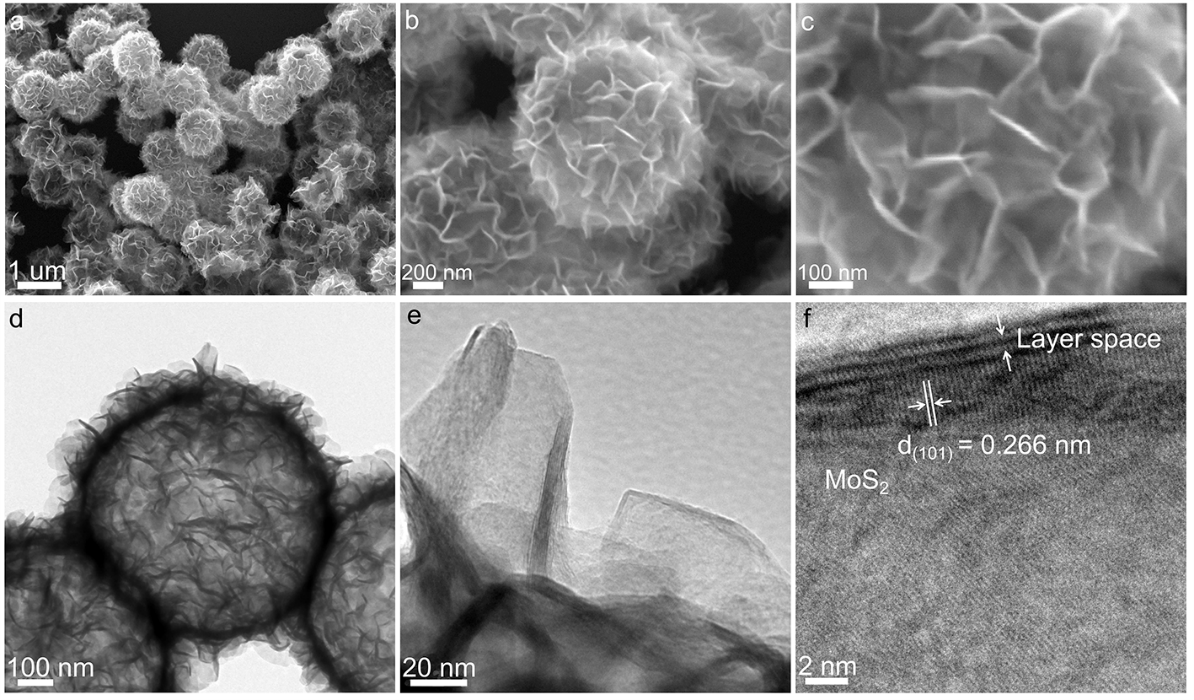


**Figure S12.** a-c) FESEM, d,e) TEM, and f) HRTEM images of the hollow MoS_2_.


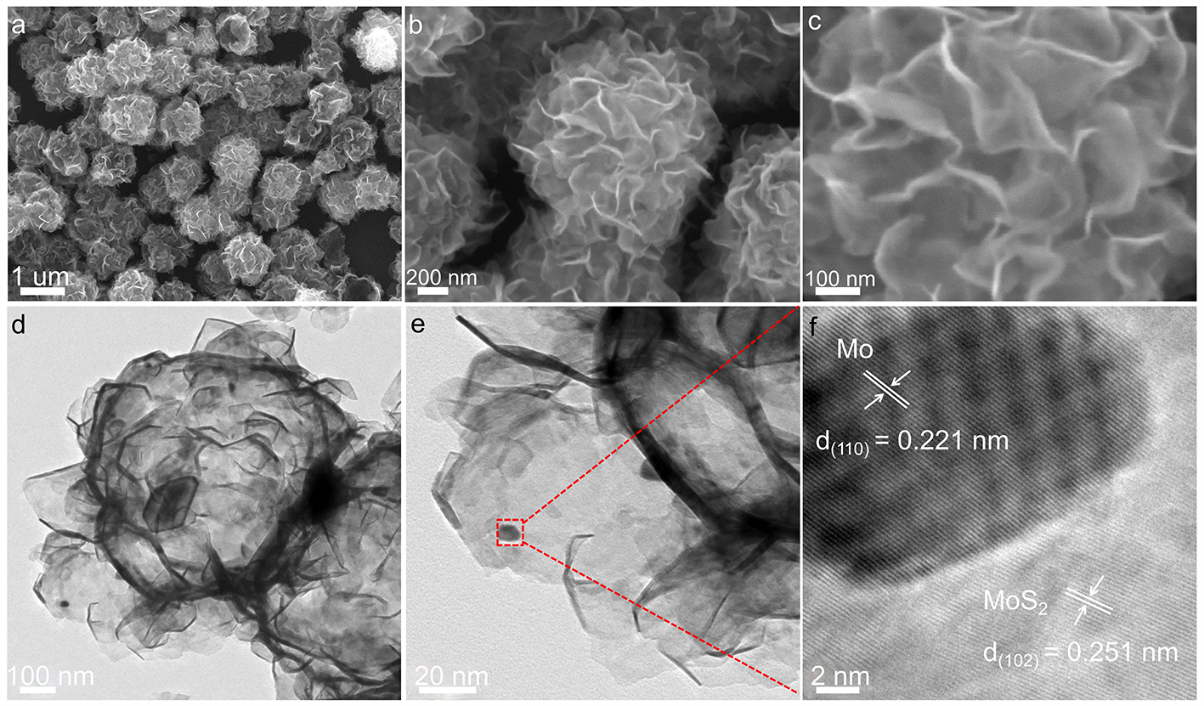


**Figure S13.** a-c) FESEM, d,e) TEM, and f) HRTEM images of the hollow Mo/MoS_2_.


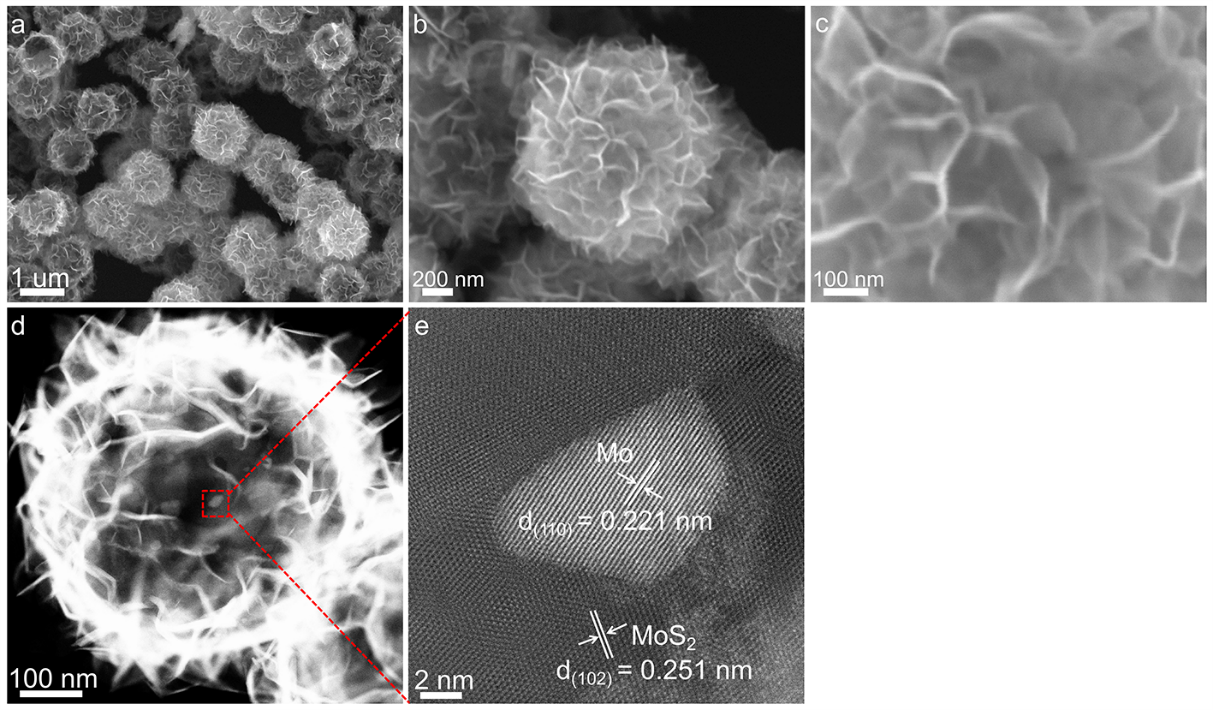


**Figure S14.** a-c) FESEM and d,e) AC-TEM images of the hollow Mo/MoS_V1_.


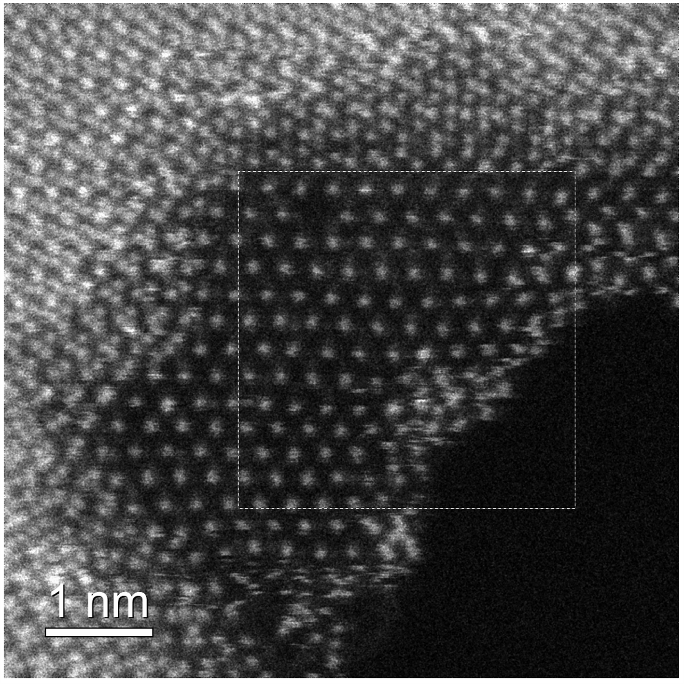


**Figure S15.** AC-TEM image of the hollow Mo/MoS_V1_.


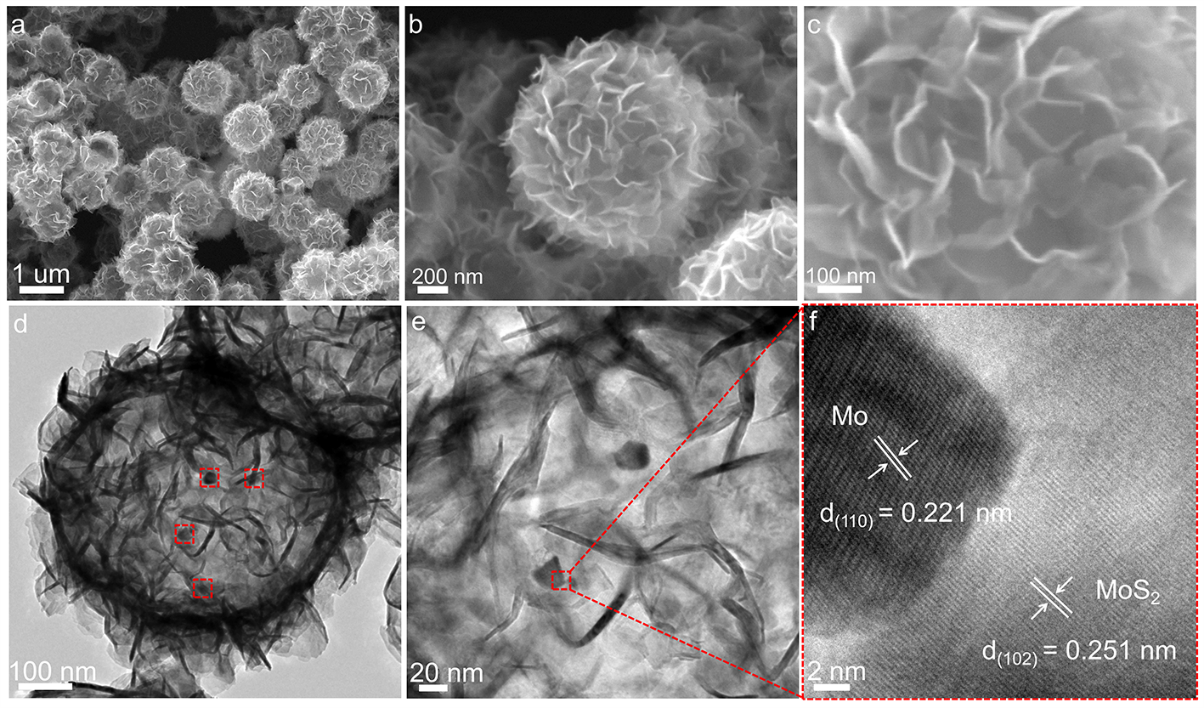


**Figure S16.** a-c) FESEM, d,e) TEM, and f) HRTEM images of the hollow Mo/MoS_V2_.


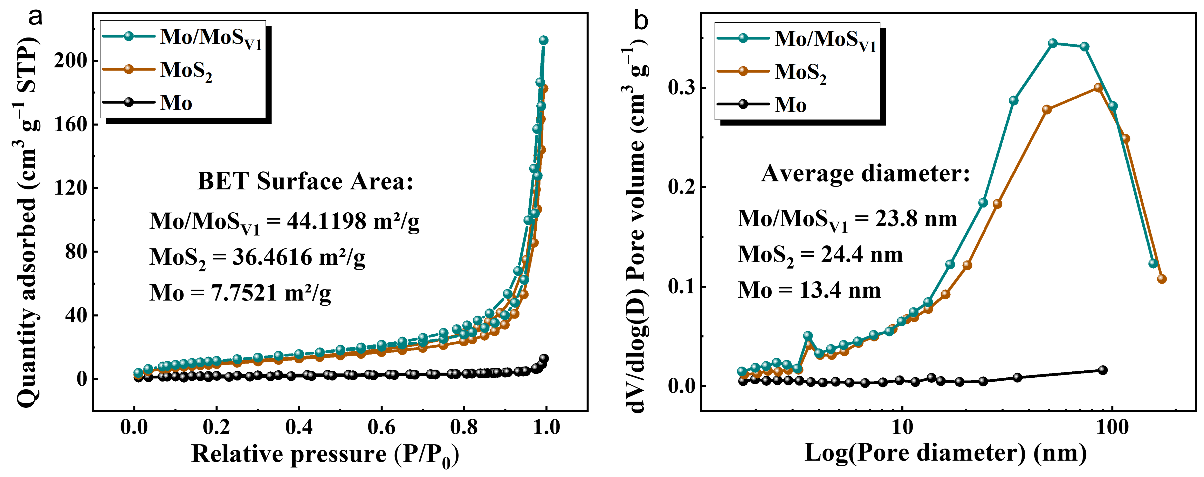


**Figure S17.** a) The N_2_ absorption and desorption curves and b) the corresponding pore size distributions of the Mo/MoS_V1_, MoS_2_, and Mo.


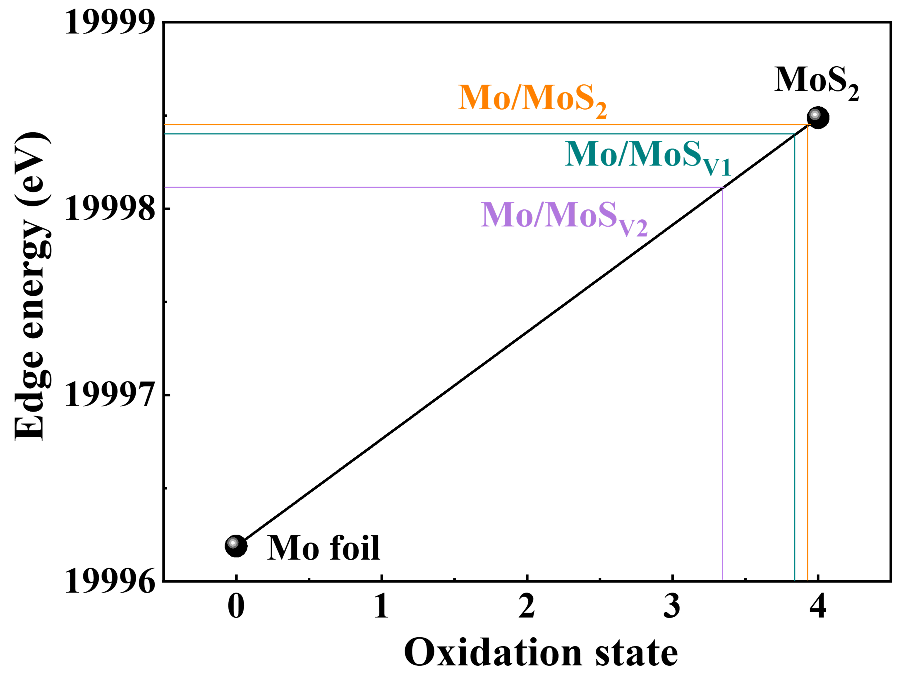


**Figure S18.** The oxidation state of Mo obtained from Mo K-edge XANES for the Mo foil, MoS_2_ reference, Mo/MoS_2_, Mo/MoS_V1_, and Mo/MoS_V2_.


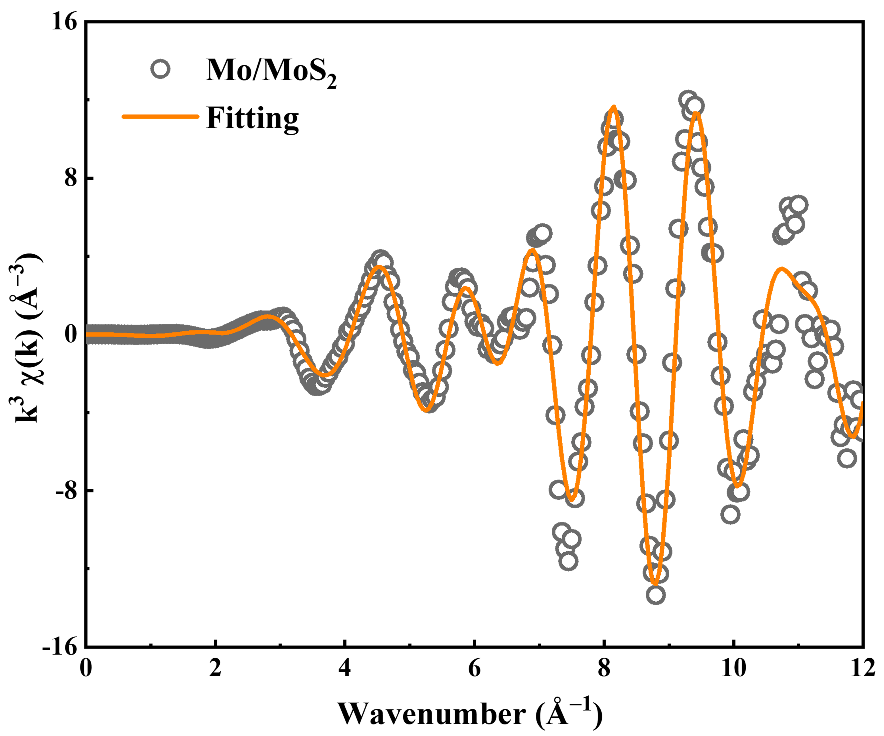


**Figure S19.** The fitting result of the EXAFS spectra for the Mo/MoS_2_ at k space.


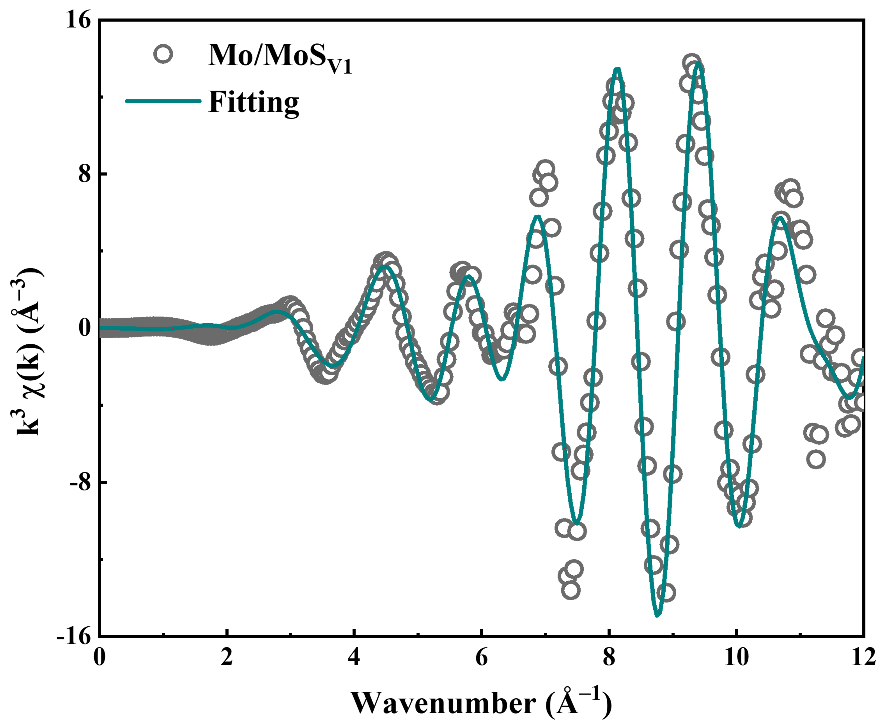


**Figure S20.** The fitting result of the EXAFS spectra for the Mo/MoS_V1_ at k space.


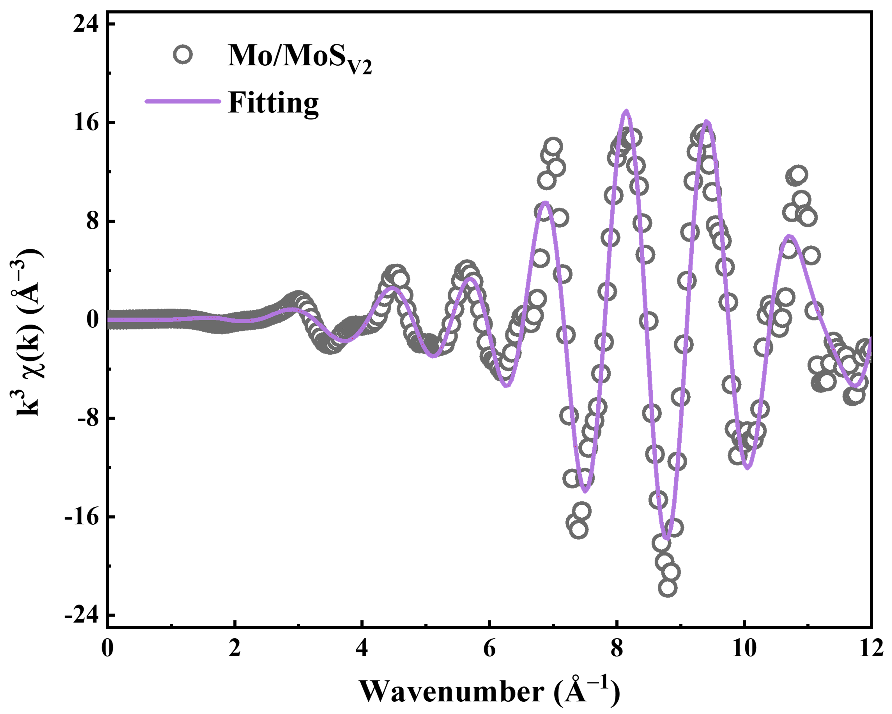


**Figure S21.** The fitting result of the EXAFS spectra for the Mo/MoS_V2_ at k space.


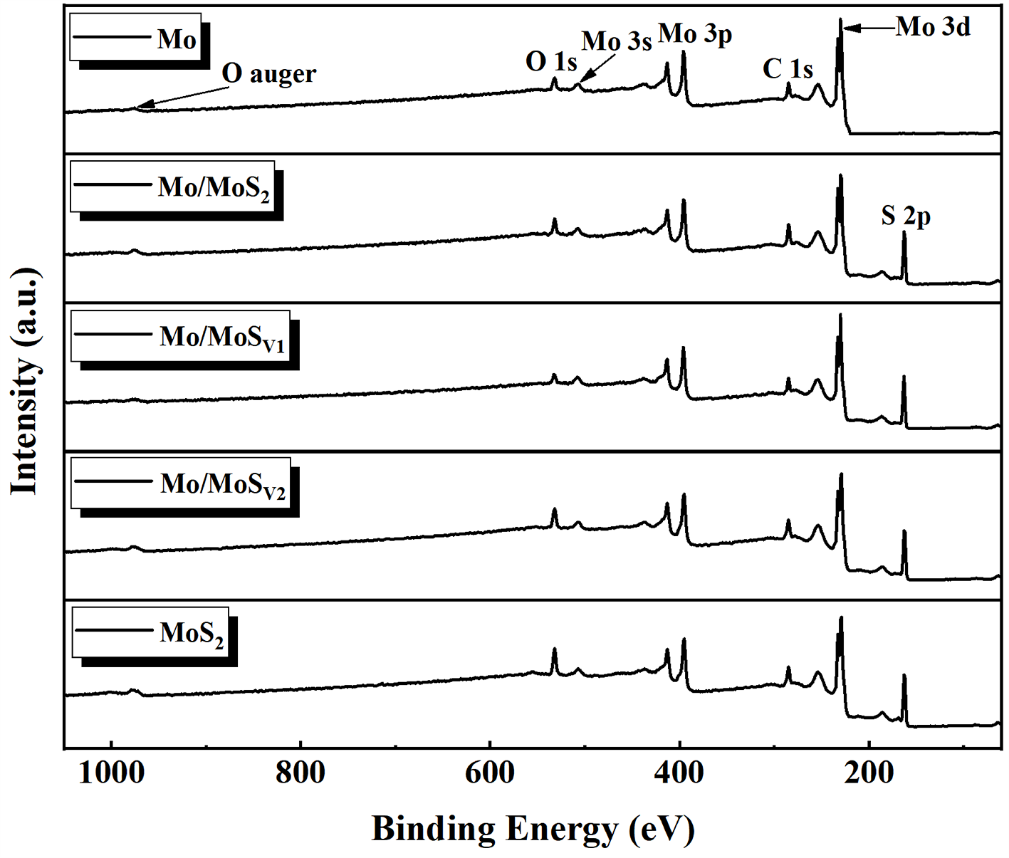


**Figure S22.** The survey XPS spectra for the Mo, MoS_2_, Mo/MoS_2_, Mo/MoS_V1_, and Mo/MoS_V2_.


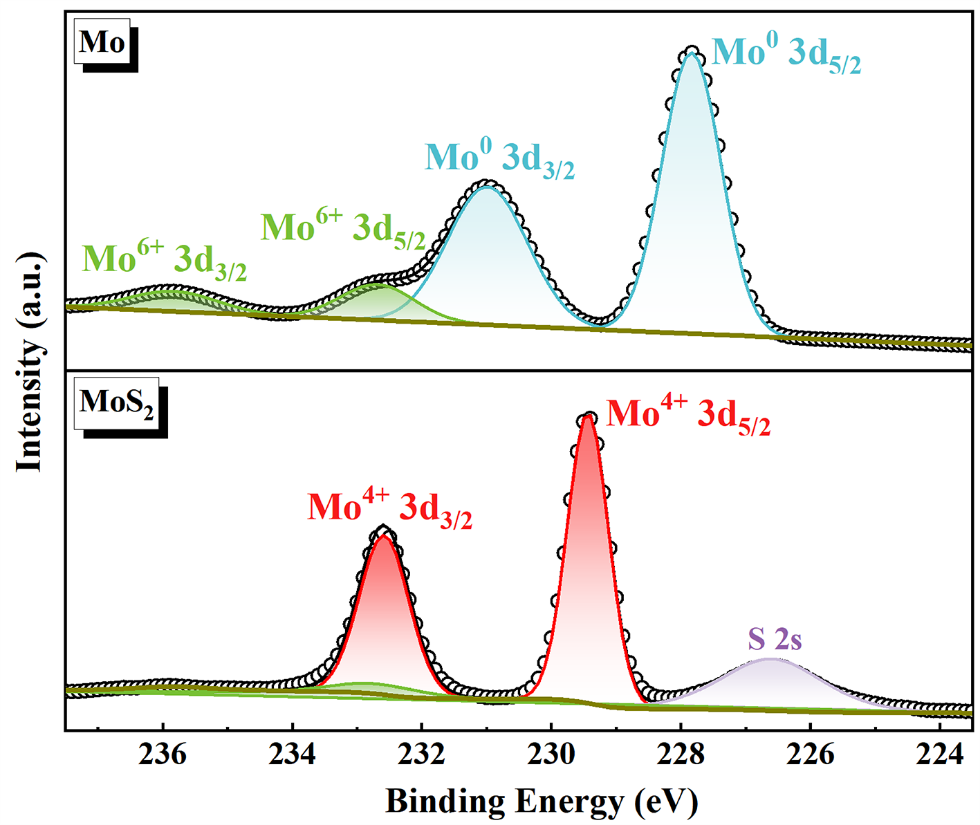


**Figure S23.** The high-resolution XPS spectra of Mo 3d for the Mo and MoS_2_.


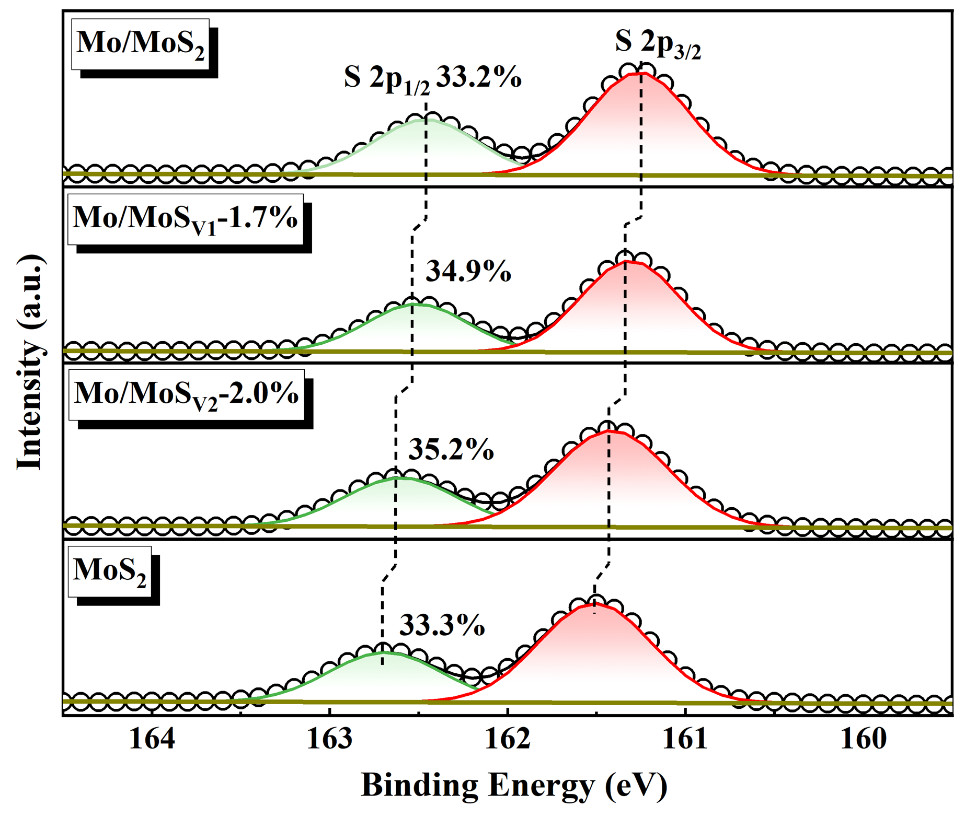


**Figure S24.** The high-resolution XPS spectra of S 2p for the MoS_2_, Mo/MoS_2_, Mo/MoS_V1_, and Mo/MoS_V2_.


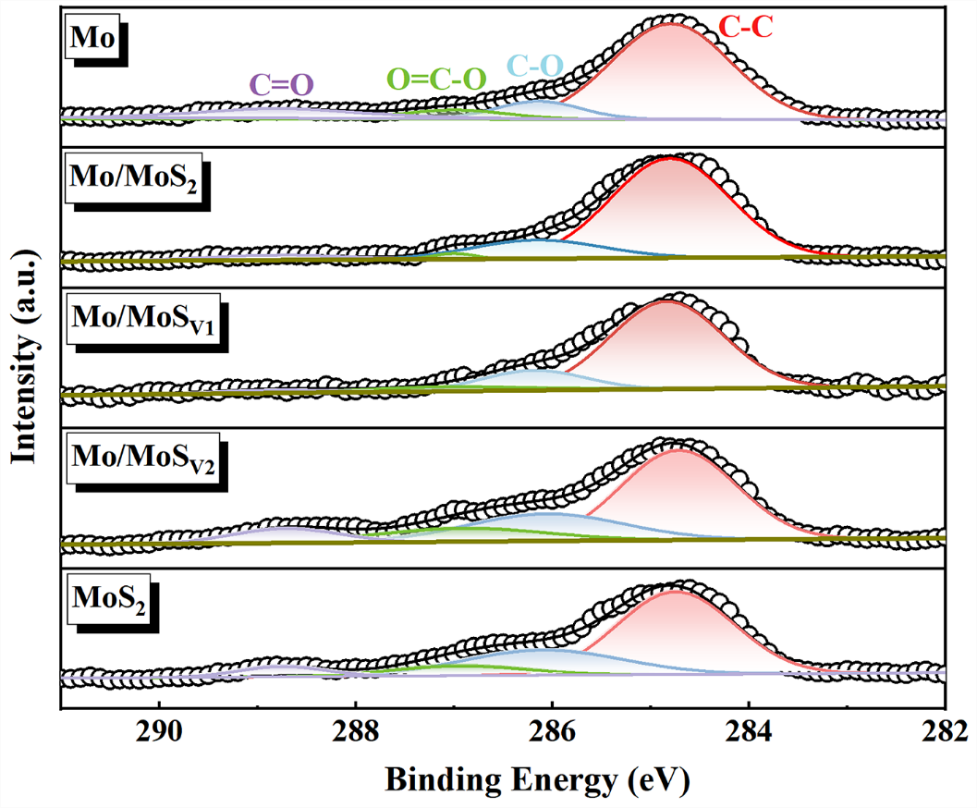


**Figure S25.** The high-resolution XPS spectra of C 1s for the Mo, MoS_2_, Mo/MoS_2_, Mo/MoS_V1_, and Mo/MoS_V2_.


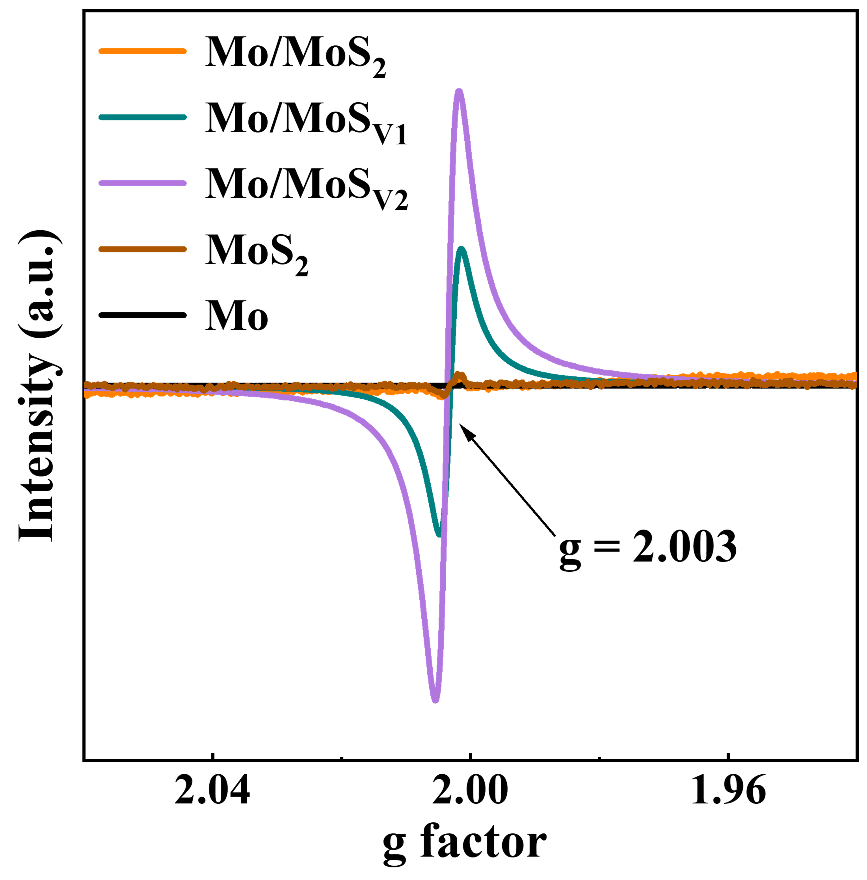


**Figure S26.** EPR spectra for the Mo, MoS_2_, Mo/MoS_2_, Mo/MoS_V1_, and Mo/MoS_V2_.


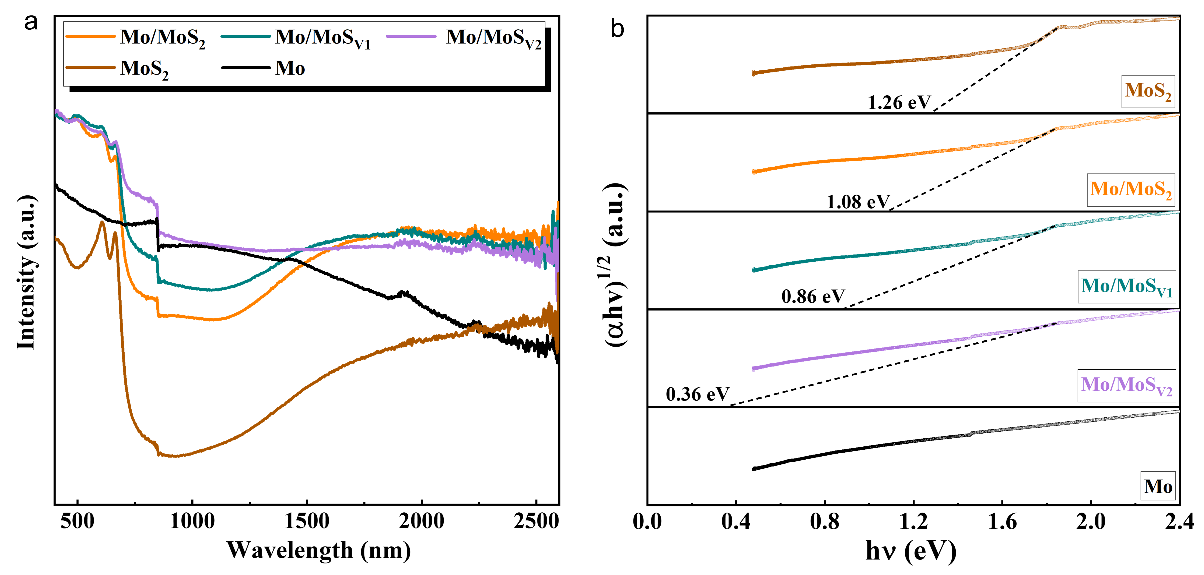


**Figure S27.** a) UV-Vis diffuse reflection spectroscopy and b) the corresponding Tauc plots of the Mo, MoS_2_, Mo/MoS_2_, Mo/MoS_V1_, and Mo/MoS_V2_.


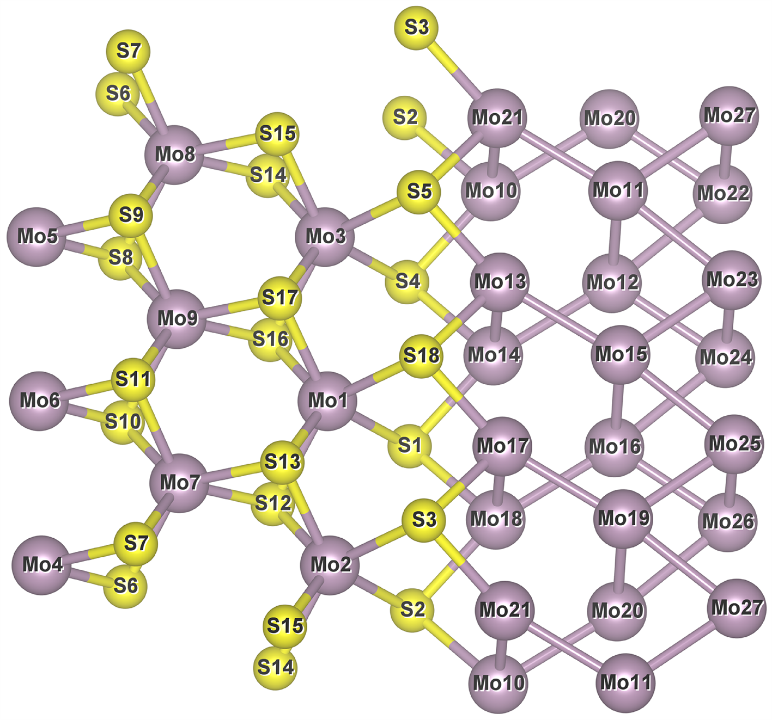


**Figure S28.** The sequence of every atom in the Mo/MoS_2_ model.


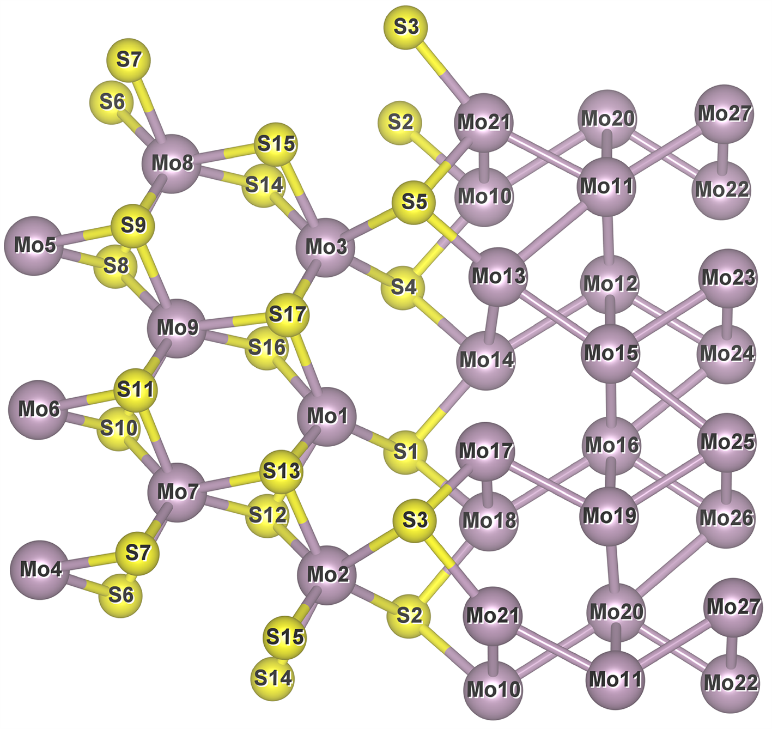


**Figure S29.** The sequence of every atom in the Mo/MoS_V1_ model.


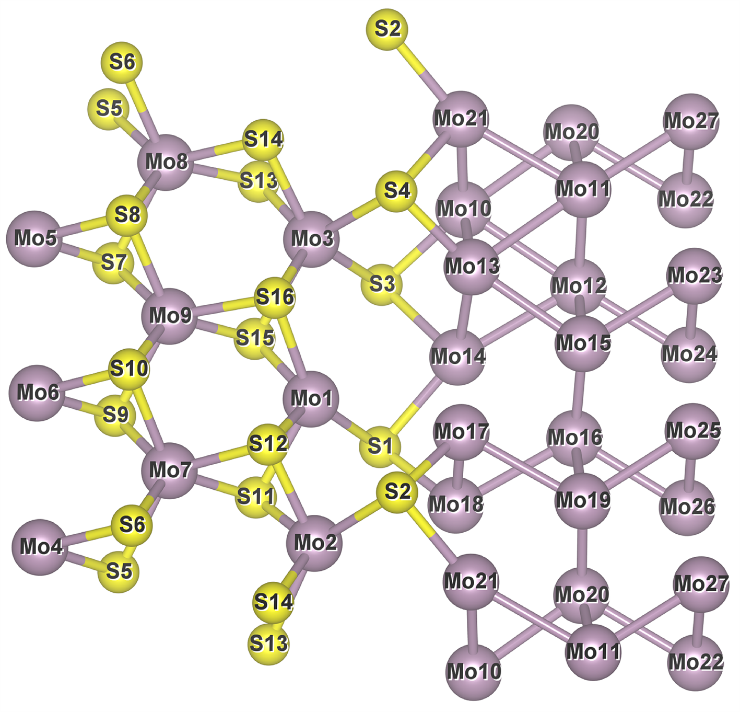


**Figure S30.** The sequence of every atom in the Mo/MoS_V2_ model.


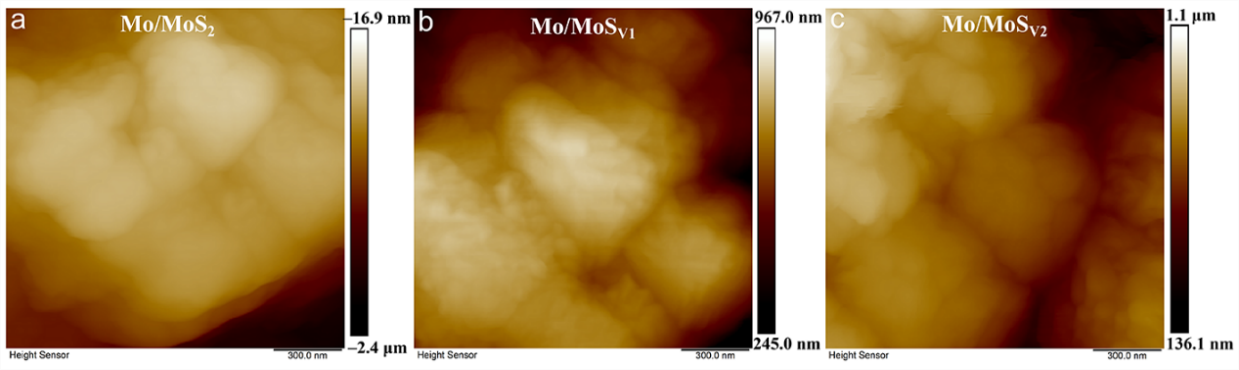


**Figure S31.** AFM height images of a) the Mo/MoS_2_, b) Mo/MoS_V1_, and c) Mo/MoS_V2_.


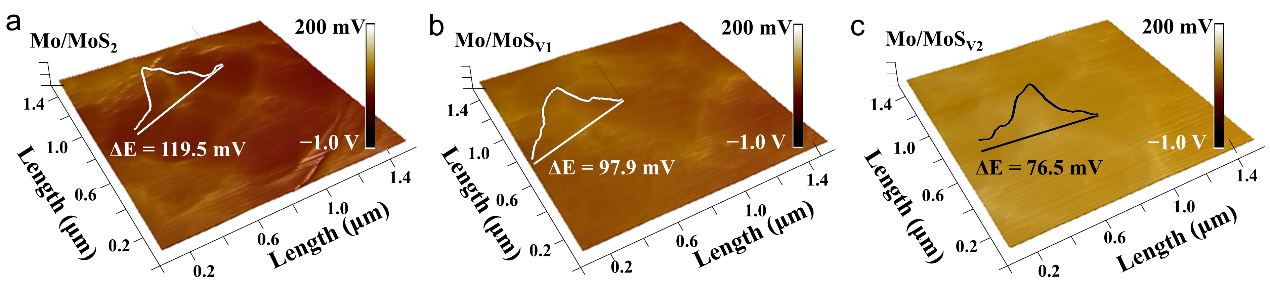


**Figure S32.** The surface potential distributions of a) the Mo/MoS_2_, b) Mo/MoS_V1_, and c) Mo/MoS_V2_ measured by KPFM.


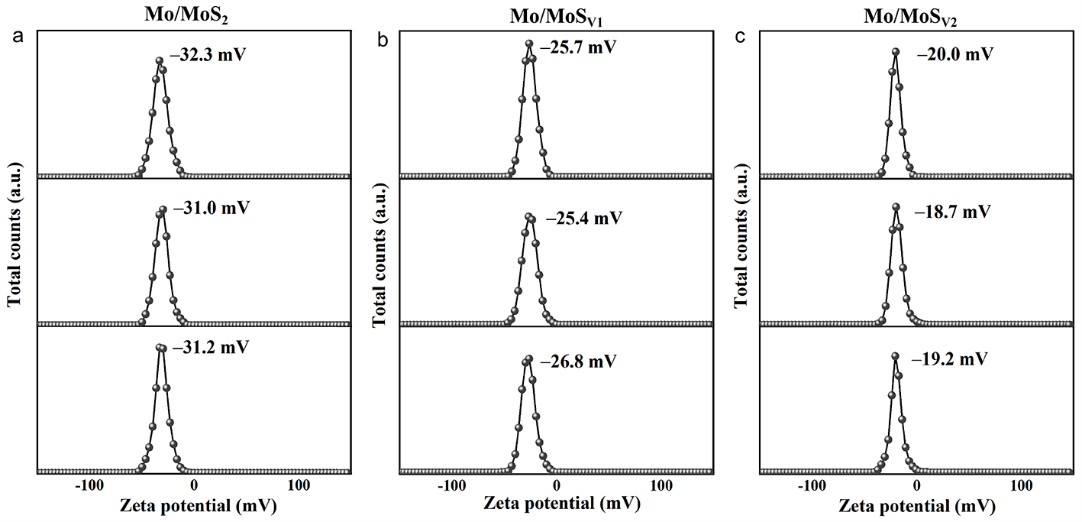


**Figure S33.** The zeta potential tests of a) the Mo/MoS_2_, b) Mo/MoS_V1_, and c) Mo/MoS_V2._


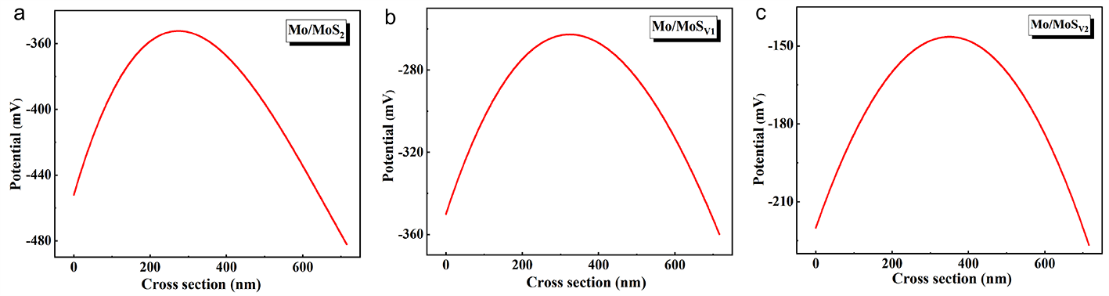


**Figure S34.** The potential distributions of interfacial electric field on a) the Mo/MoS_2_, b) Mo/MoS_V1_, and c) Mo/MoS_V2._


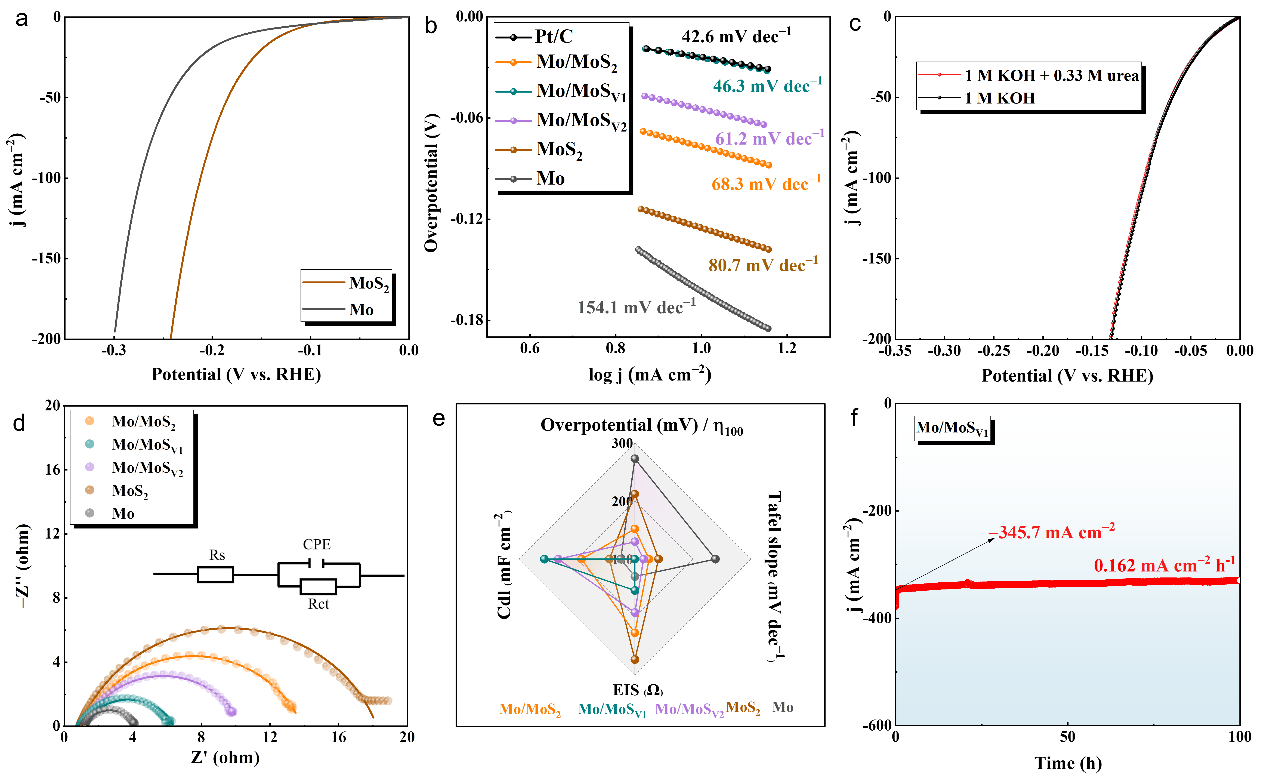


**Figure S35.** Electrochemical HER properties in alkaline electrolyte. a) LSV curves, b) Tafel slope, c) comparison of LSV curves for the Mo/MoS_V1_ in 1 M KOH+0.33 M urea and 1 M KOH, d) EIS Nyquist plots, e) summary of overpotential at 100 mA cm^−2^, C_dl_, EIS, and Tafel slope, and f) the chronoamperometric measurement under 350 mA cm^−2^.


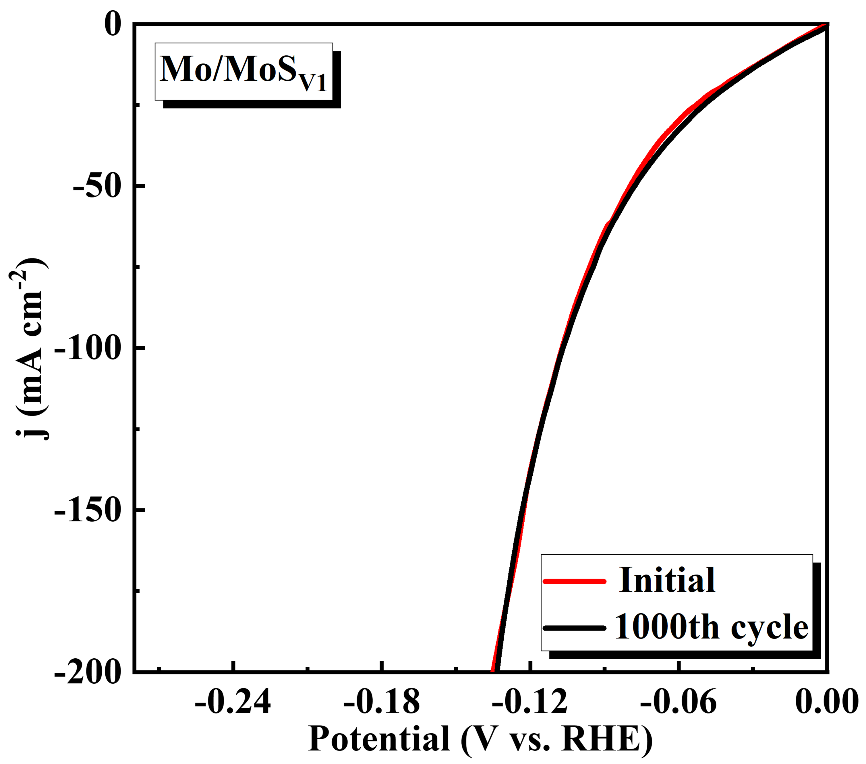


**Figure S36.** The polarization curves of Mo/MoS_V1_ before and after 1000 cycles.


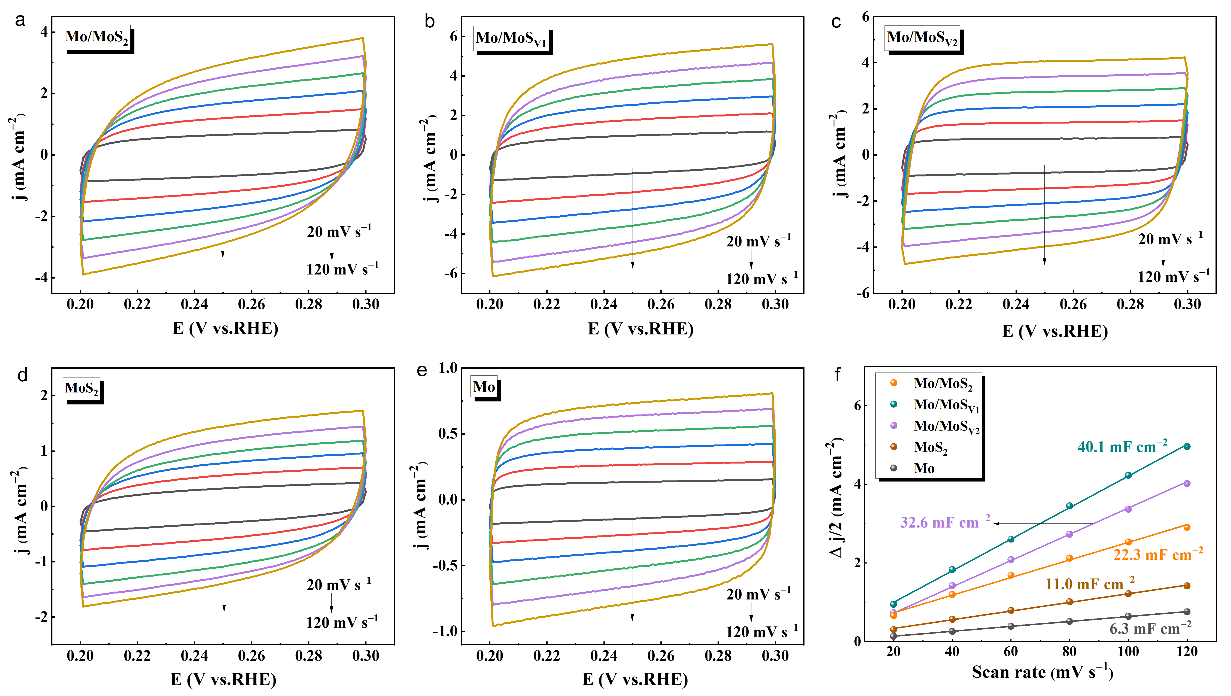


**Figure S37.** a-e) Cyclic voltammetry (CV) test from 20–120 mV s^−1^ and f) C_dl_ results of the as-prepared catalysts for HER.


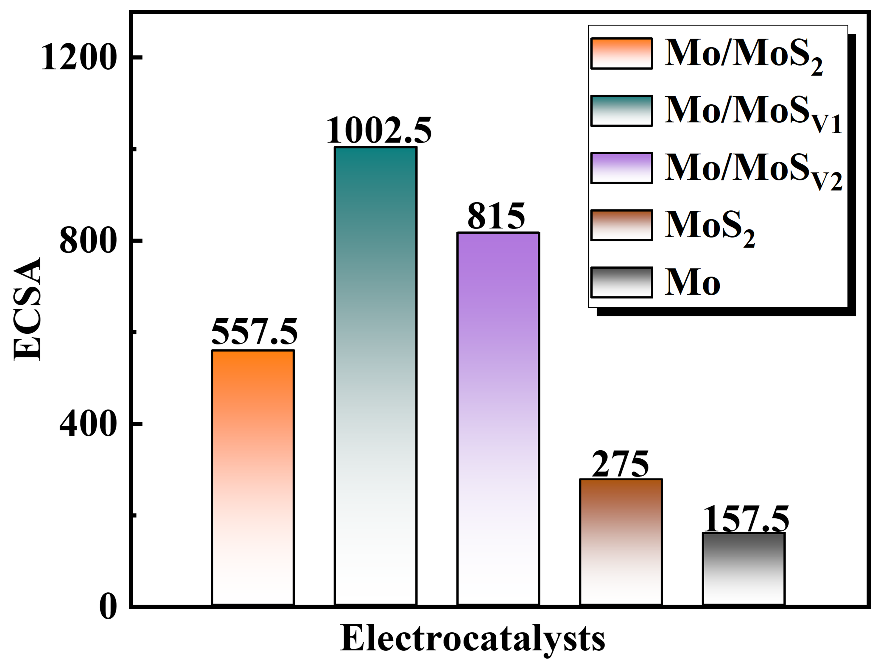


**Figure S38.** The ECSA values of the Mo/MoS_2_, Mo/MoS_V1_, Mo/MoS_V2_, MoS_2_ and Mo electrocatalysts for HER.


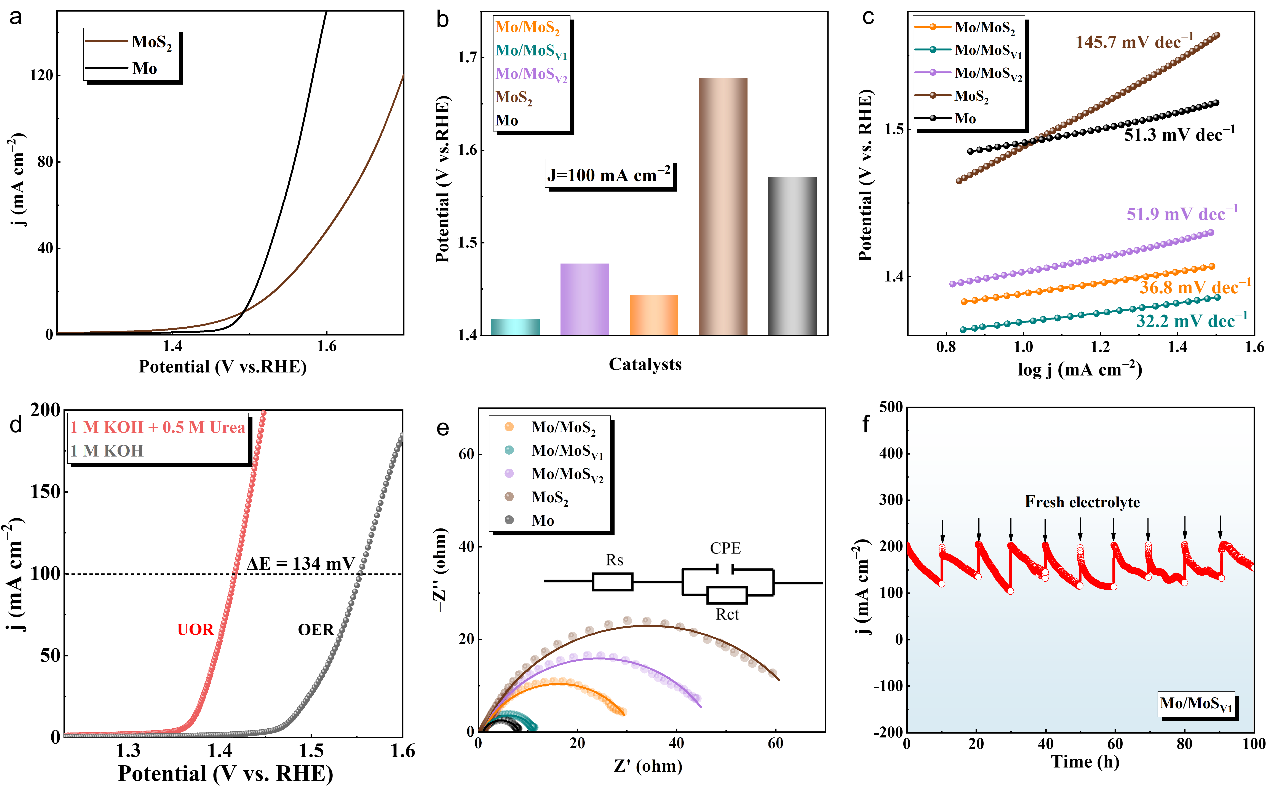


**Figure S39.** Electrochemical properties for UOR. a) LSV curves, b) the comparison of potentials at 100 mA cm^-2^, c) Tafel slopes, d) comparison of LSV curves for the Mo/MoS_V1_ in the OER and UOR, e) EIS Nyquist plots, and f) stability test for 100 h.


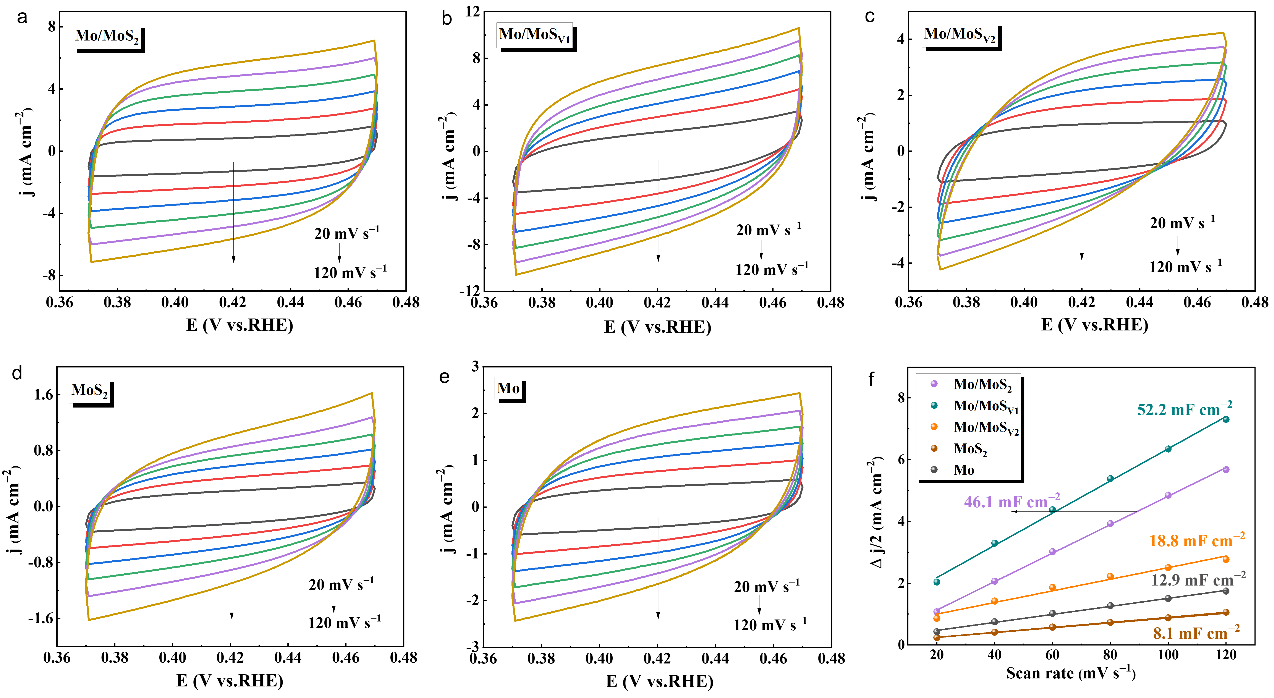


**Figure S40.** a-e) CV measurements and f) C_dl_ results of the as-prepared catalysts for UOR.


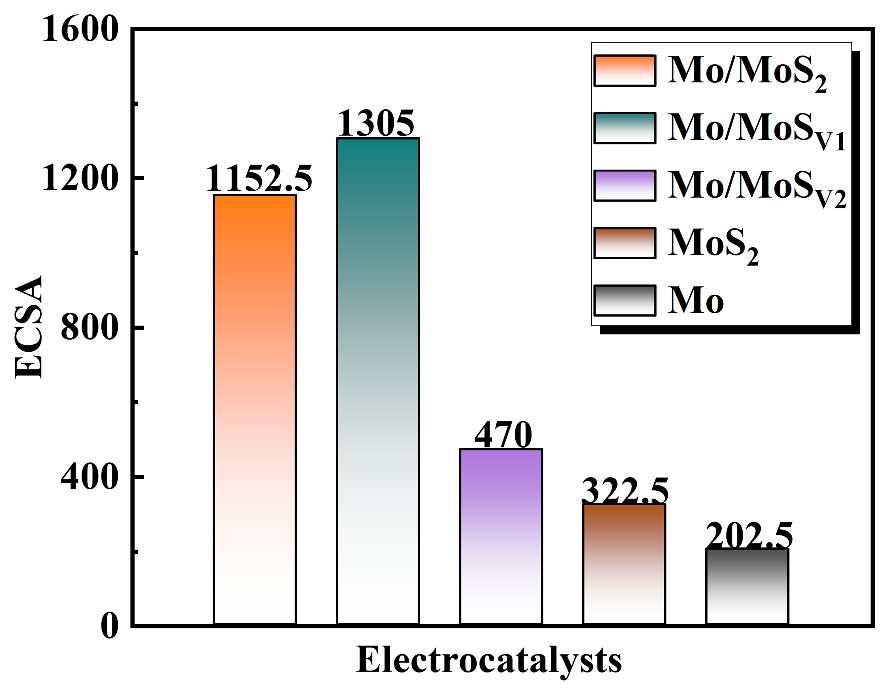


**Figure S41.** The ECSA values of the Mo/MoS_2_, Mo/MoS_V1_, Mo/MoS_V2_, MoS_2_ and Mo electrocatalysts for UOR.


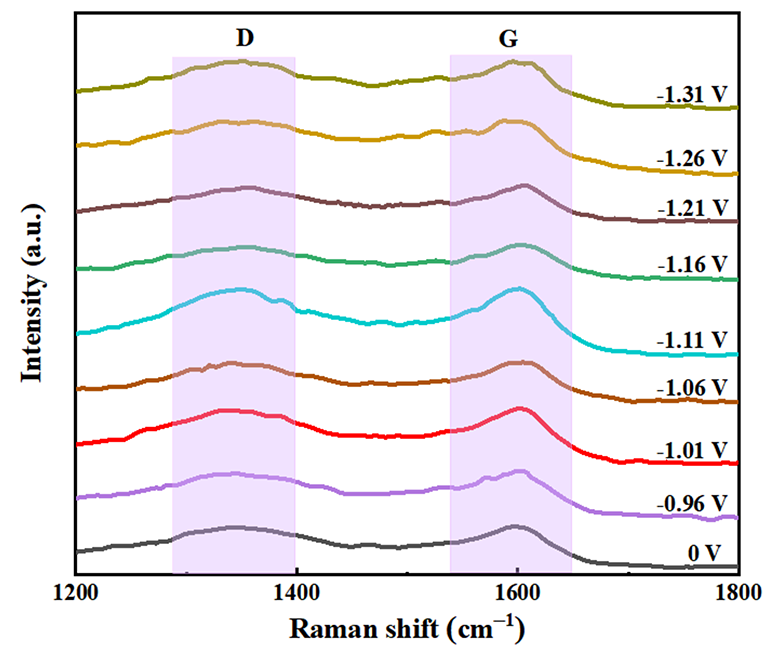


**Figure S42.** The in situ Raman spectra of the Mo/MoS_V1_ for HER.


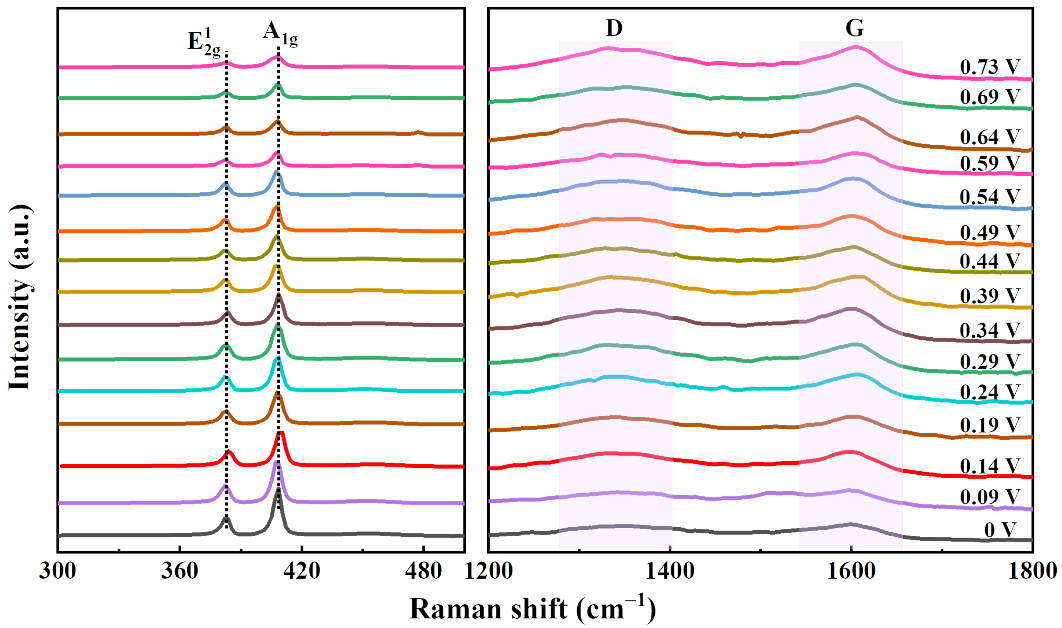


**Figure S43.** The in situ Raman spectra of the Mo/MoS_V1_ for UOR.


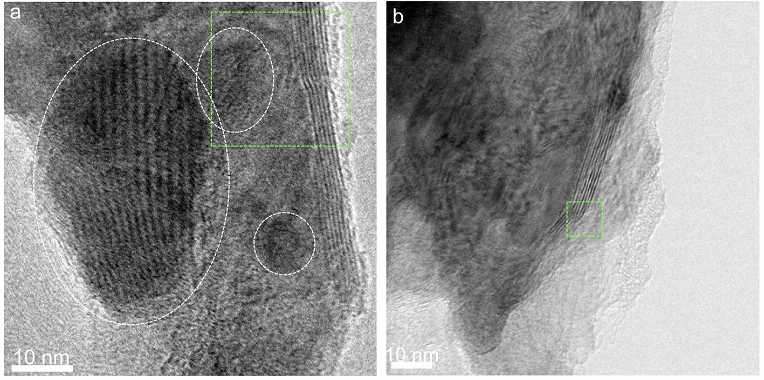


**Figure S44.** HRTEM images of the Mo/MoS_V1_ after a) HER and b) UOR.


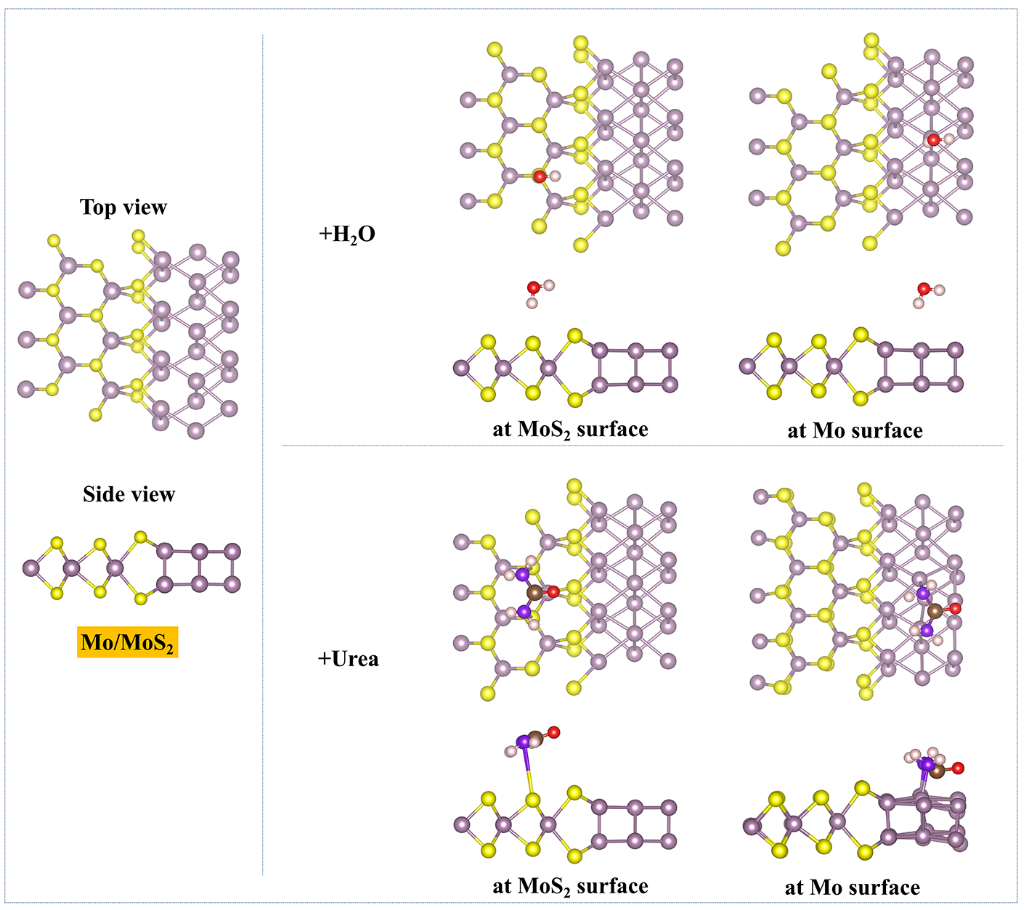


**Figure S45.** The adsorption structure models of H_2_O and urea molecules at MoS_2_ and Mo surfaces.


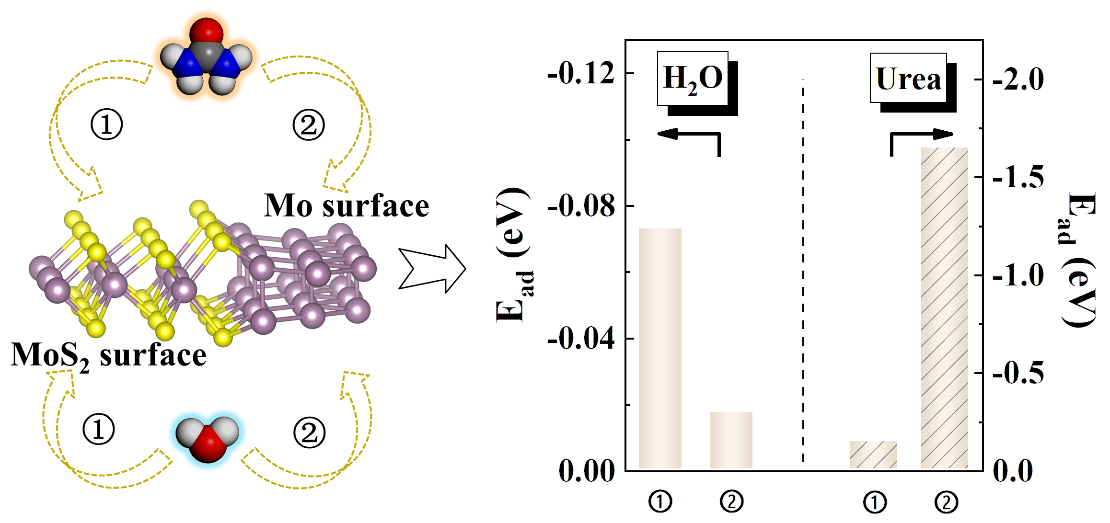


**Figure S46.** The adsorption energies of H_2_O and urea molecule at different sites of the Mo/MoS_2_.


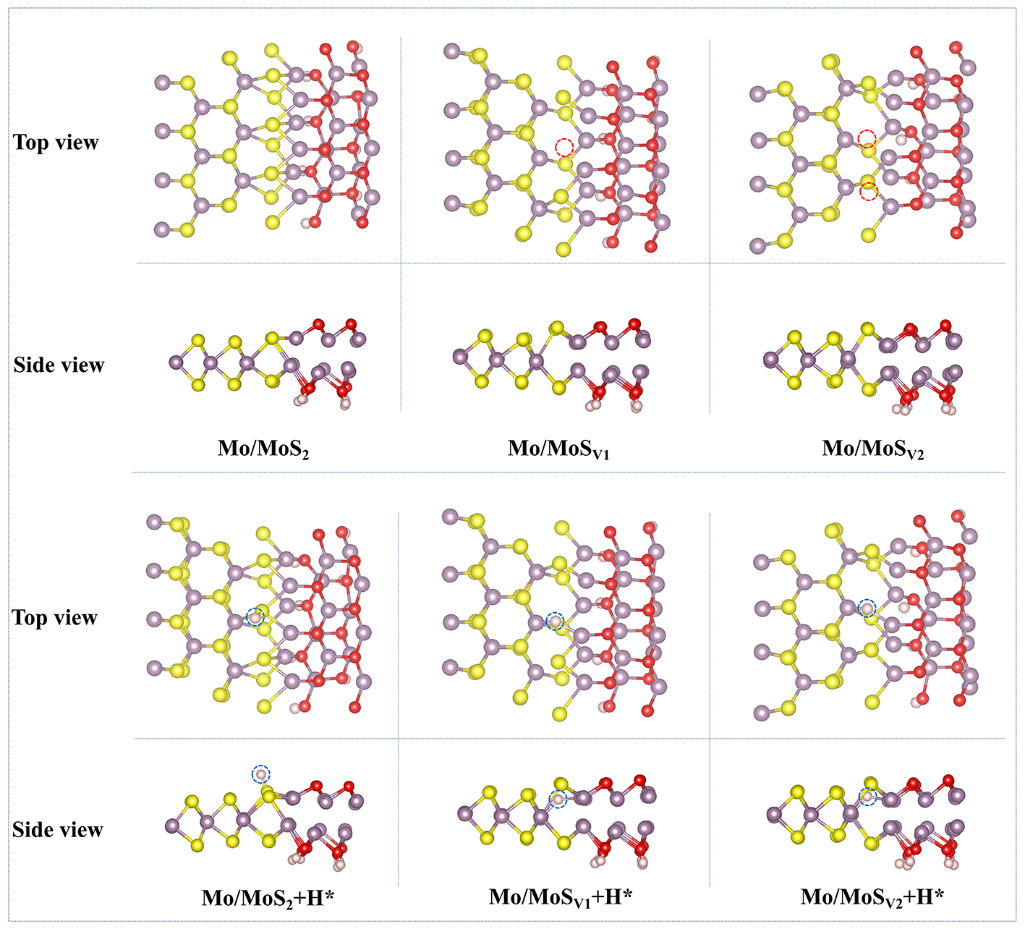


**Figure S47.** The optimal structures of H* adsorption at the Mo/MoS_2_, Mo/MoS_V1_, and Mo/MoS_V2._


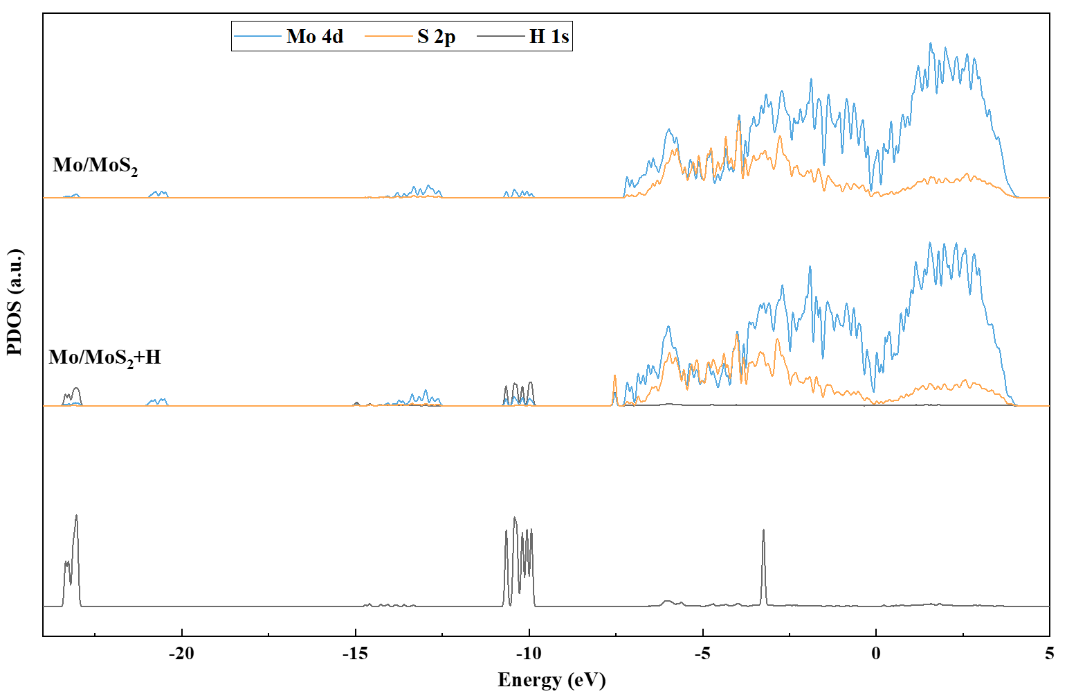


**Figure S48.** The partial density of states (PDOS) for the Mo/MoS_2_ before and after adsorption of H*_._


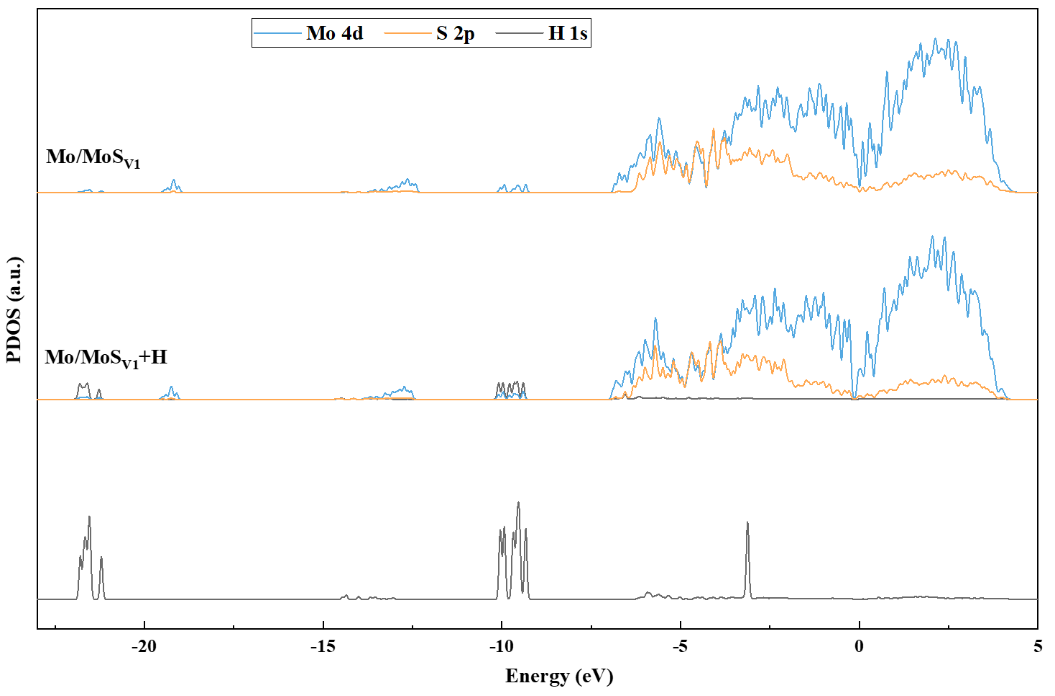


**Figure S49.** The partial density of states (PDOS) for the Mo/MoS_V1_ before and after adsorption of H*_._


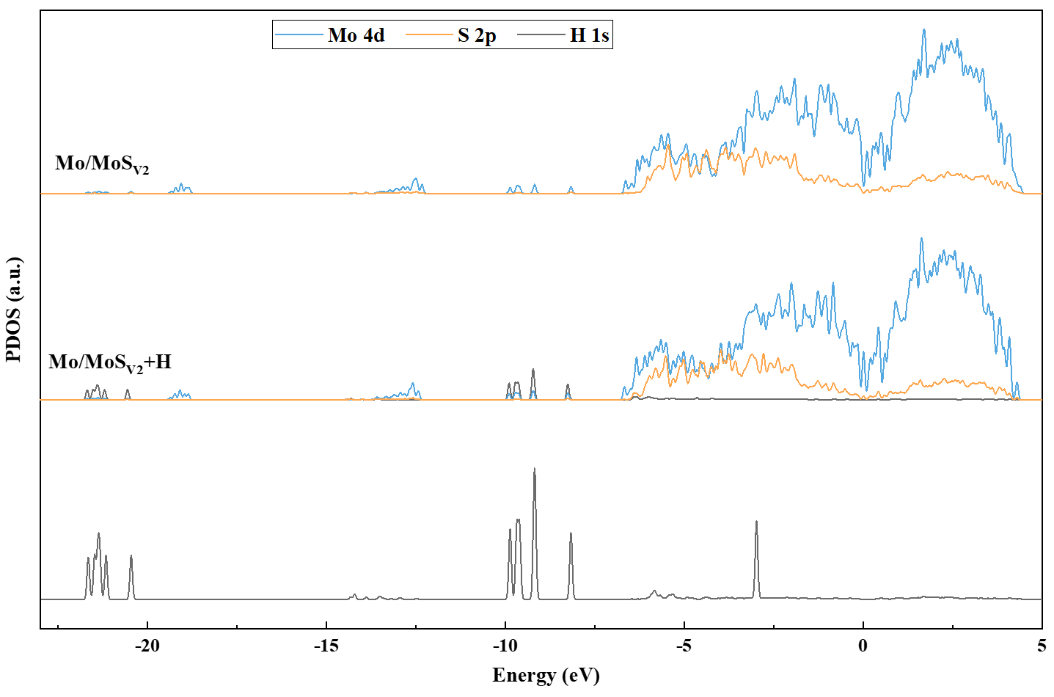


**Figure S50.** The partial density of states (PDOS) for the Mo/MoS_V2_ before and after adsorption of H*_._

_
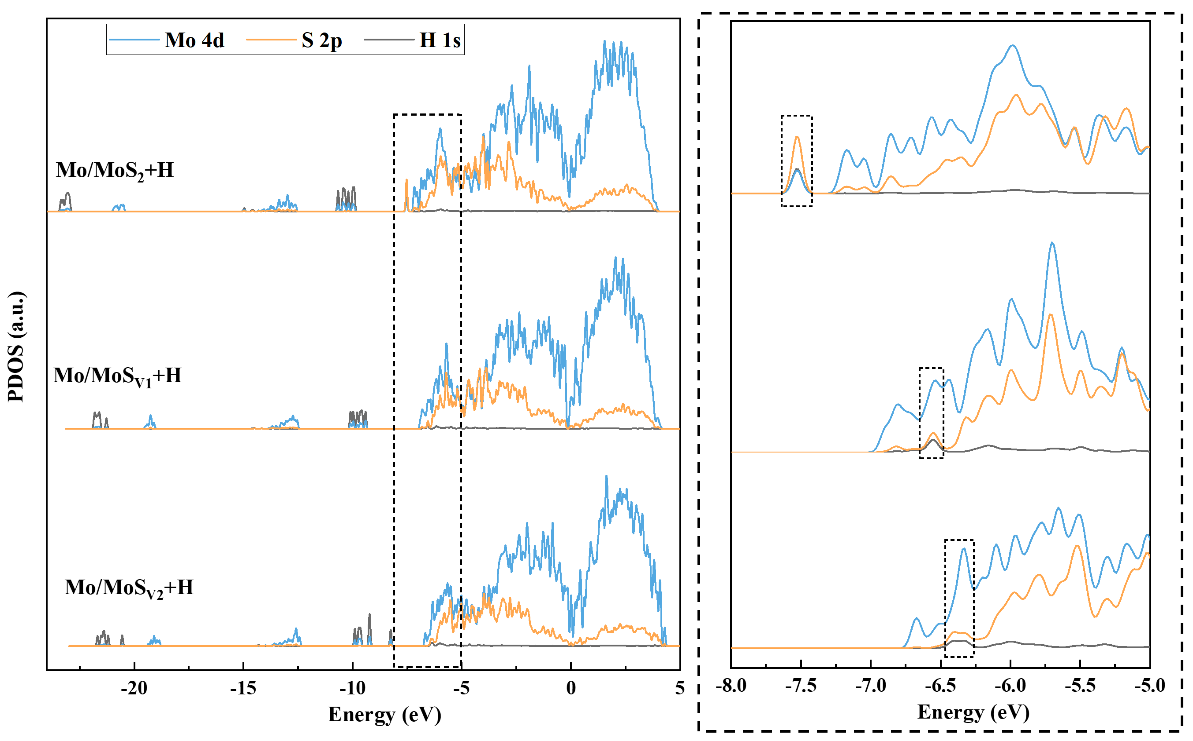
_

**Figure S51.** Comparisons of the PDOS of Mo 4d, S 2p, and H 1s in the Mo/MoS_Vn_ after adsorption of H*.

_
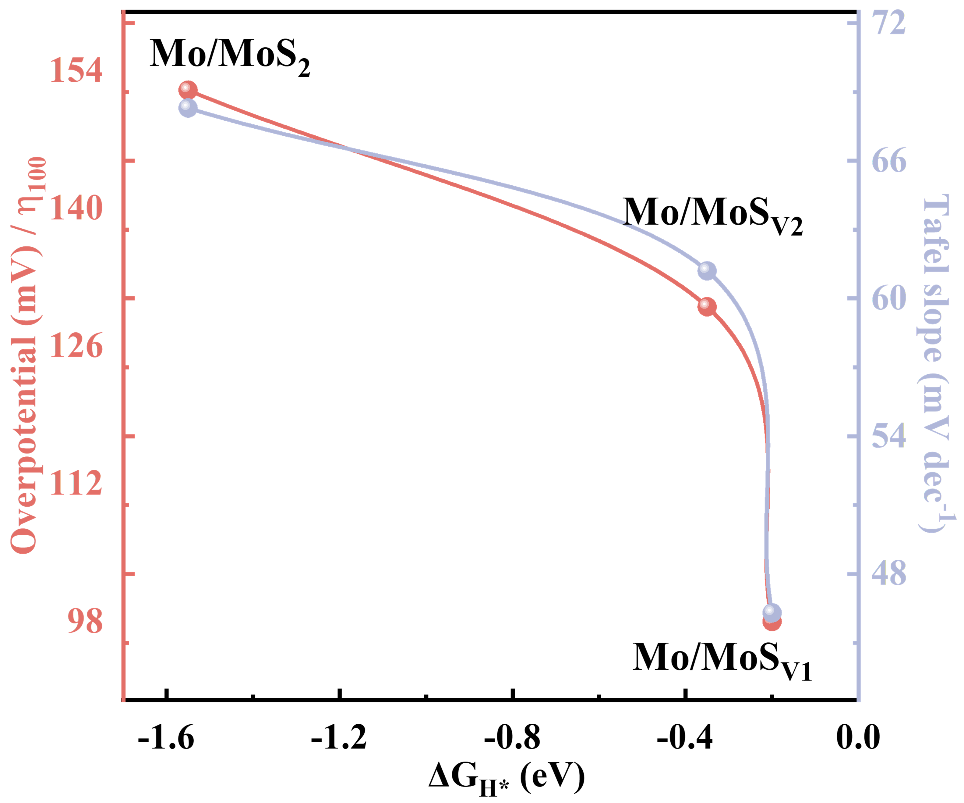
_

**Figure** **S52.** The relationship between ΔG_H*_ and catalytic performance (overpotential, Tafel slope) for HER.


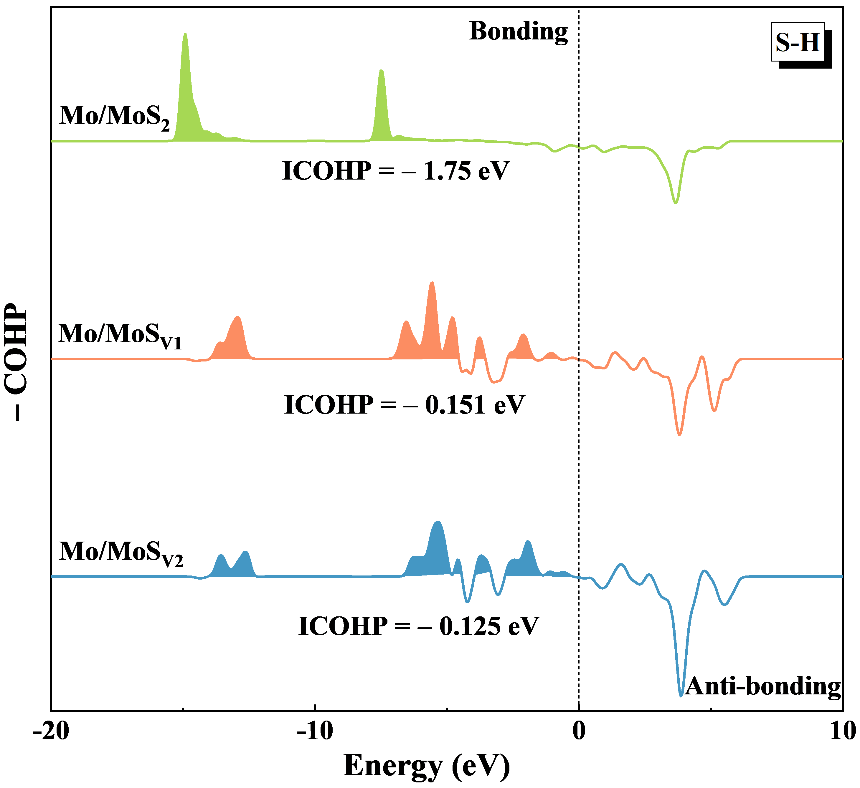


**Figure S53.** The COHP of S-H for the Mo/MoS_Vn_ in the HER process.


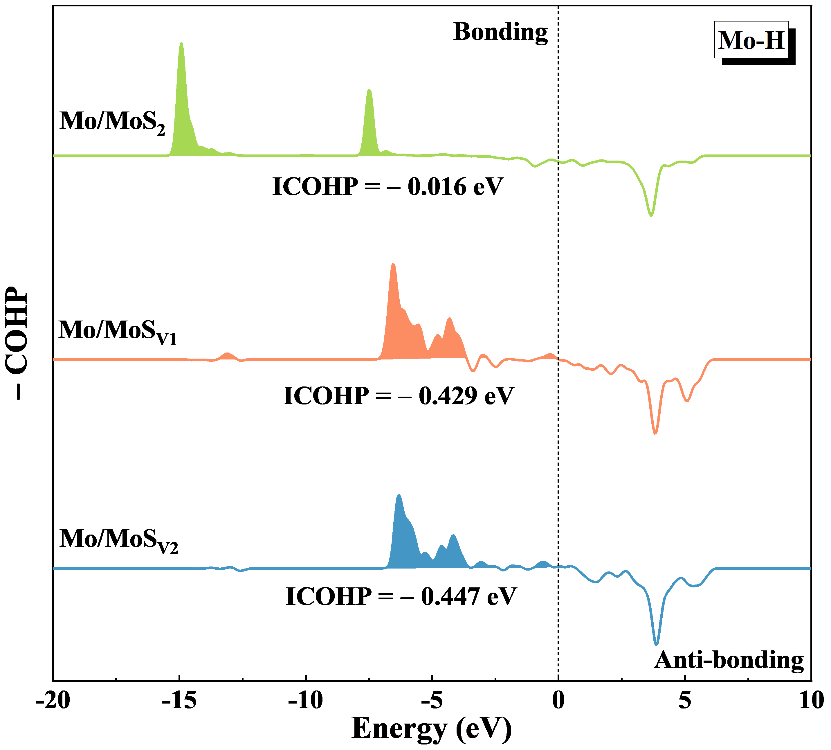


**Figure S54.** The COHP of Mo-H for the Mo/MoS_Vn_ in the HER process.


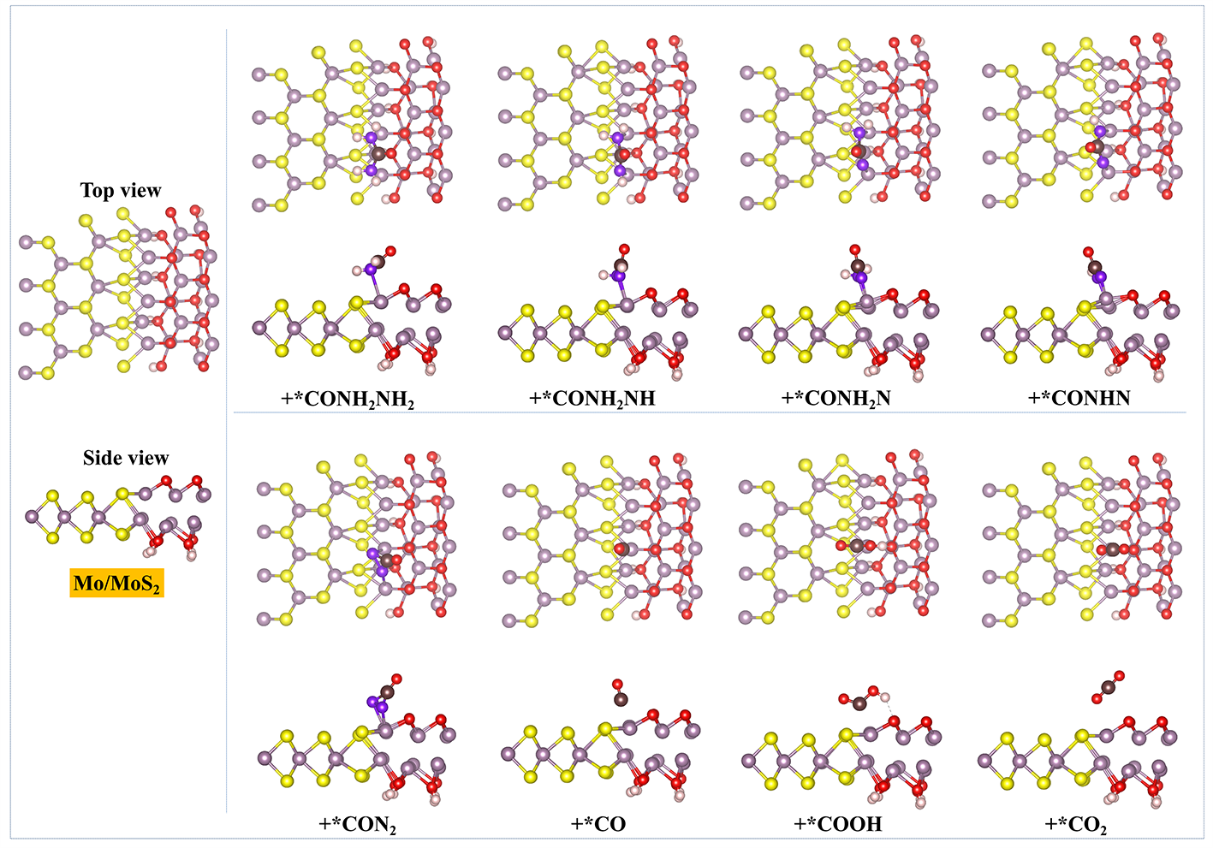


**Figure S55.** The optimal adsorption structures in the UOR process for the Mo/MoS_2_.


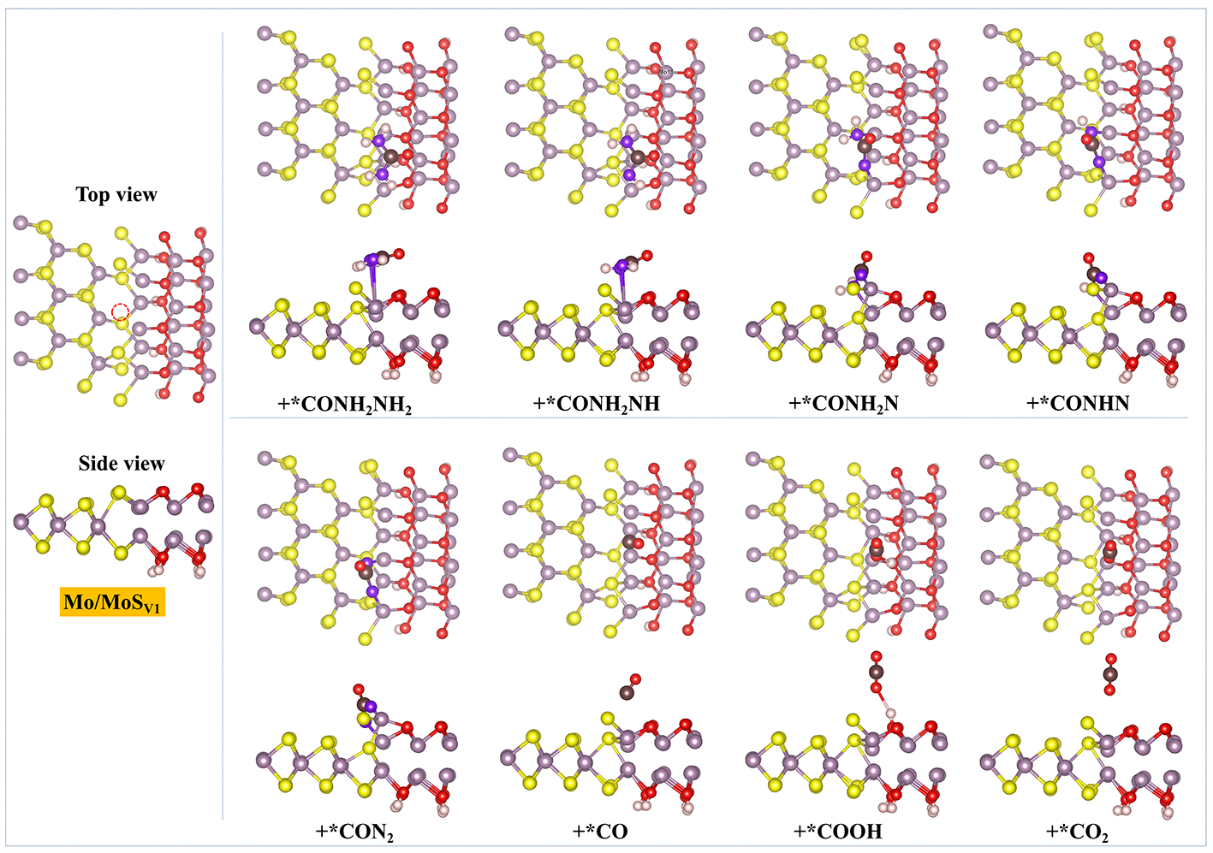


**Figure S56.** The optimal adsorption structures in the UOR process for the Mo/MoS_V1_.


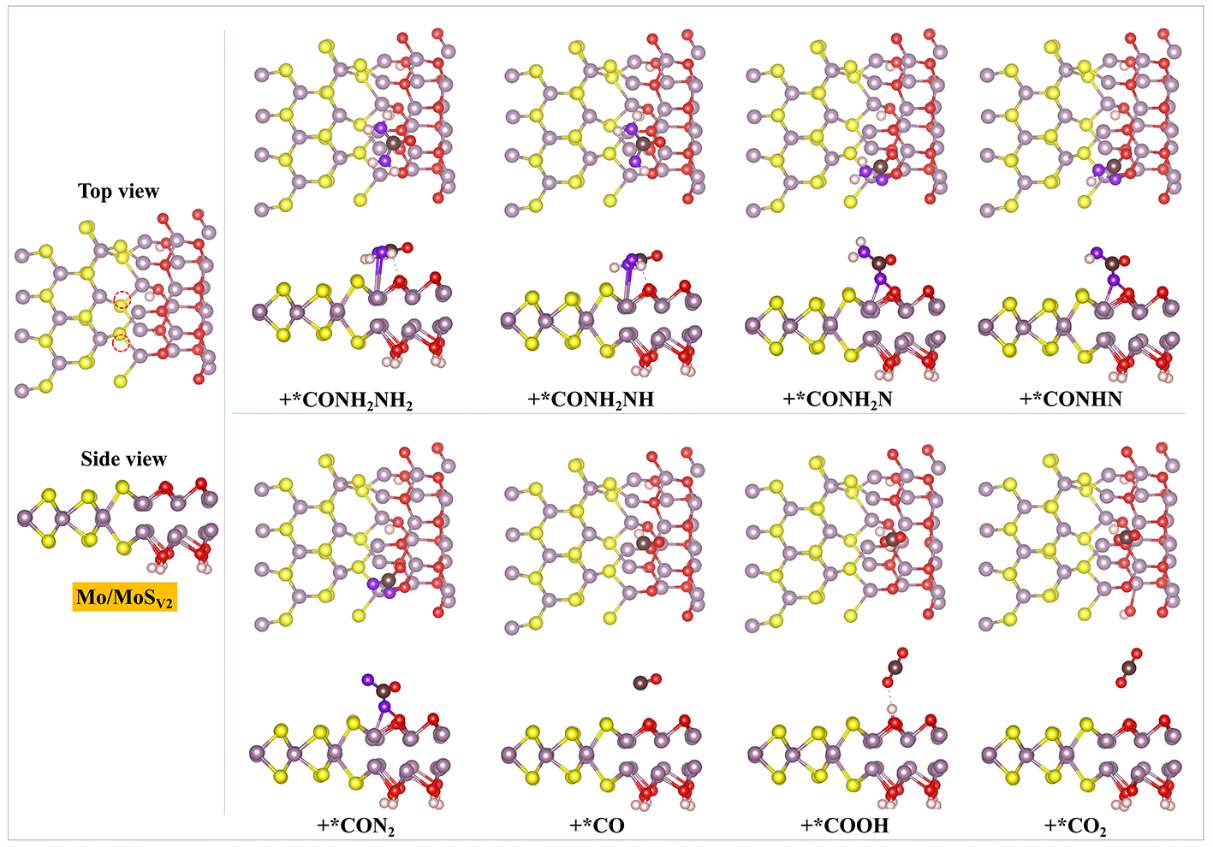


**Figure S57.** The optimal adsorption structures in the UOR process for the Mo/MoS_V2_.


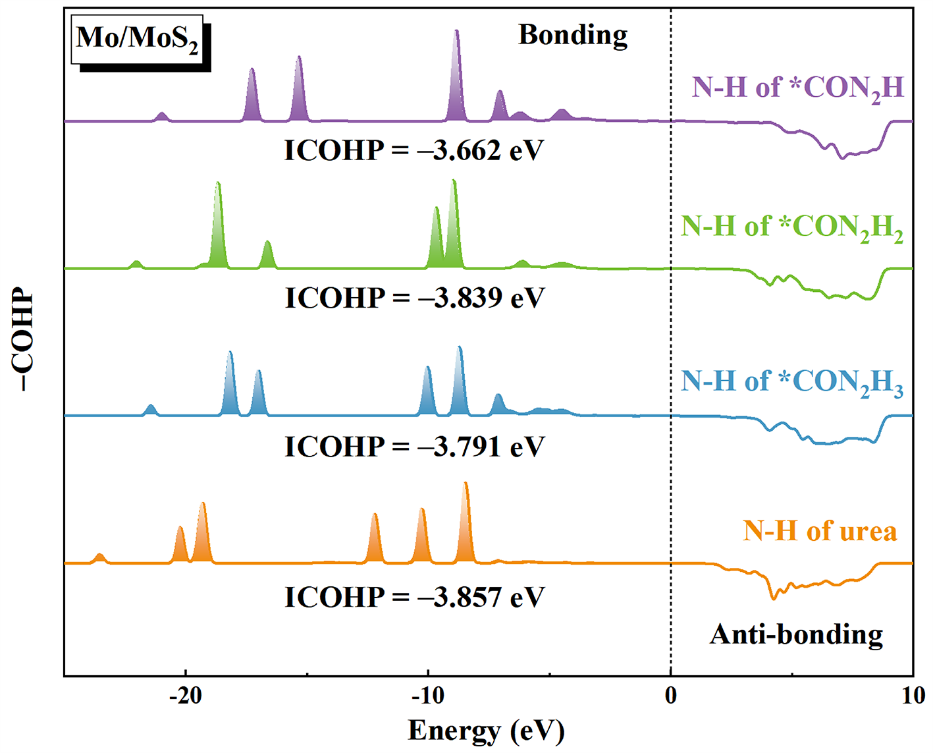


**Figure S58.** The COHP and corresponding ICOHP values of the Mo/MoS_2_ with four deprotonation steps of UOR.


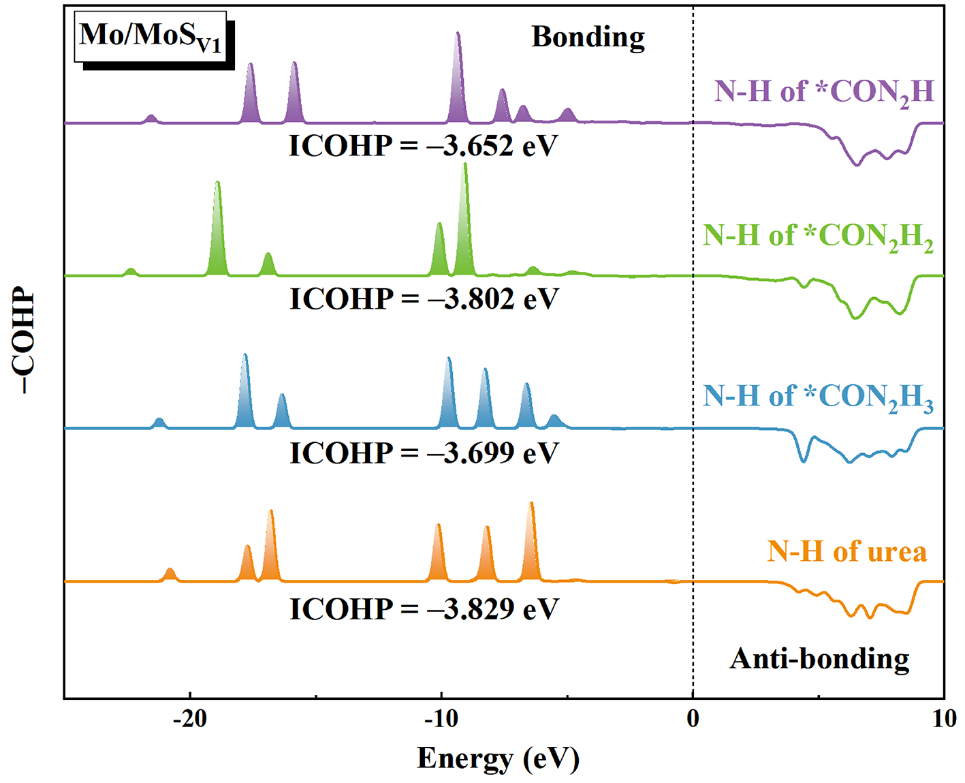


**Figure S59.** The COHP and corresponding ICOHP values of the Mo/MoS_V1_ with four deprotonation steps of UOR.


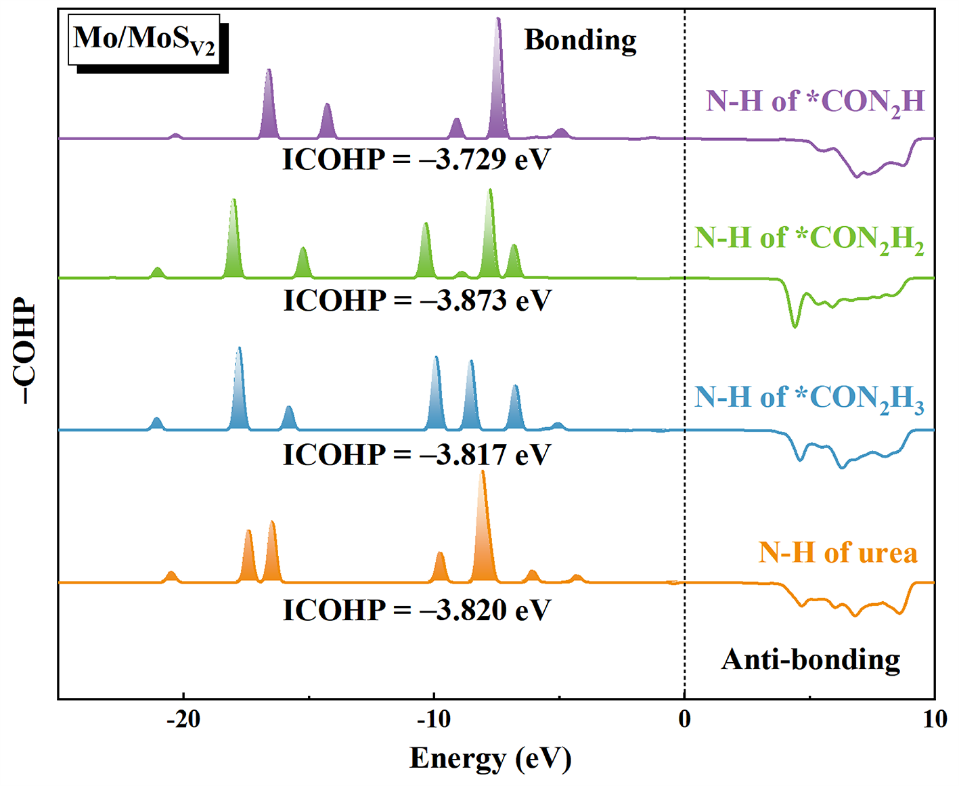


**Figure S60.** The COHP and corresponding ICOHP values of the Mo/MoS_V2_ with four deprotonation steps of UOR.


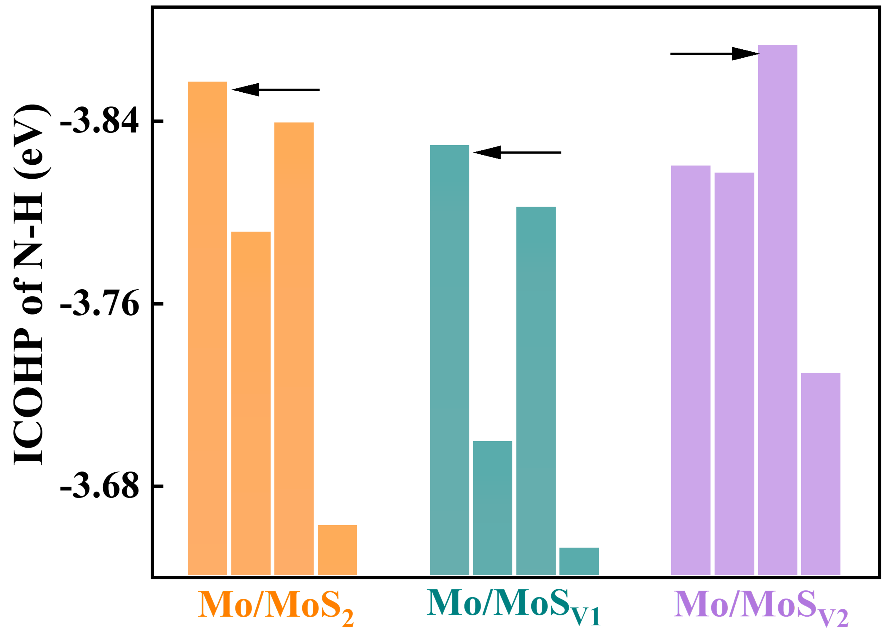


**Figure S61.** Summarization of ICOHP of N-H bond in each deprotonation step of UOR process for the Mo/MoS_2_, Mo/MoS_V1_, and Mo/MoS_V2._

_
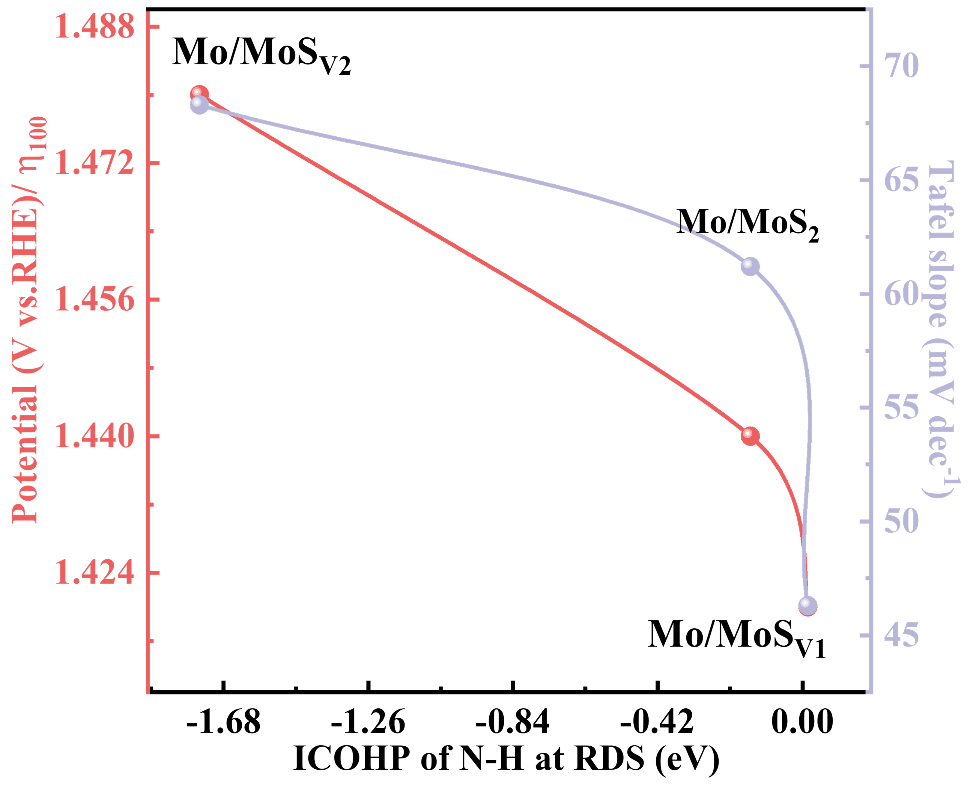
_

**Figure S62.** The relationship between ICOHP and catalytic performance (potential, Tafel slope) for UOR.


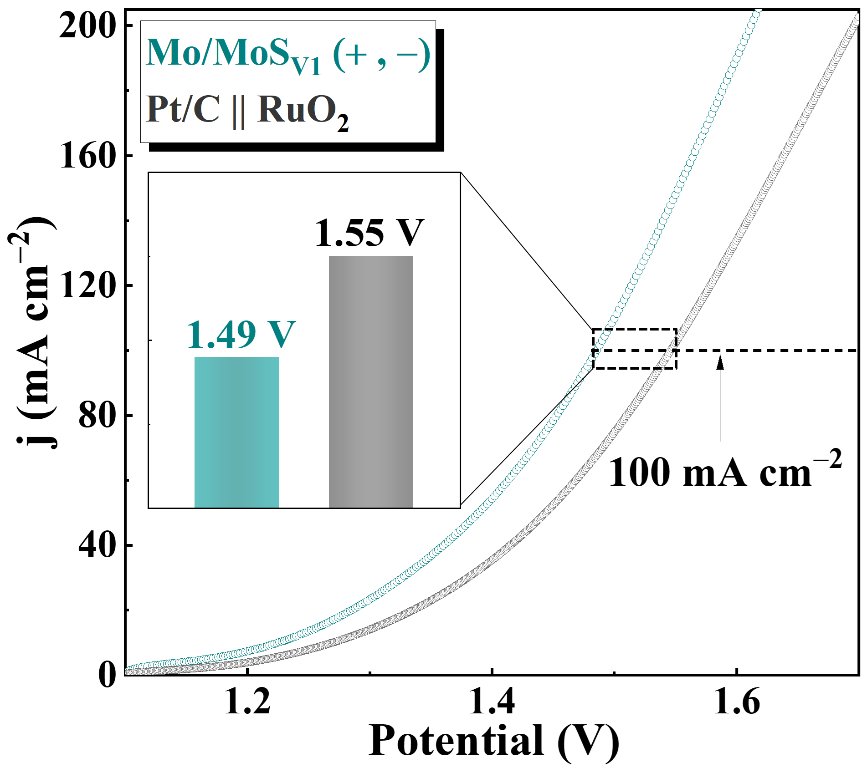


**Figure S63.** The comparisons of LSV curves for the Mo/MoS_V1_ (+,–) and Pt/C (–)||RuO_2_ (+) cells in the HER||UOR system.


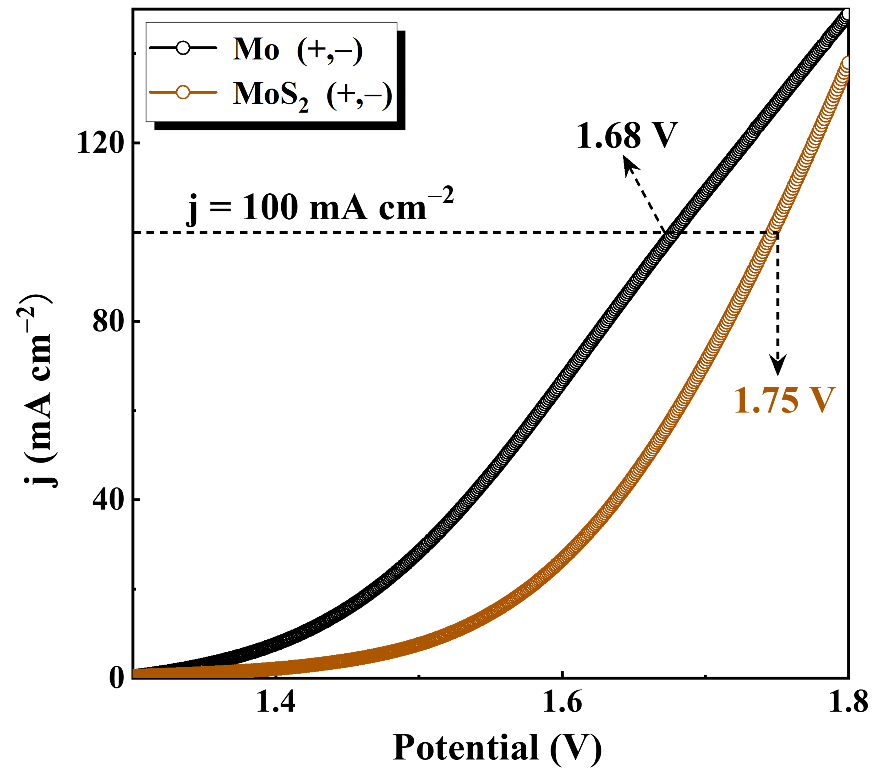


**Figure S64.** The comparisons of LSV curves for the Mo (+,–) and MoS_2_ (+,–) cells in the HER||UOR system.


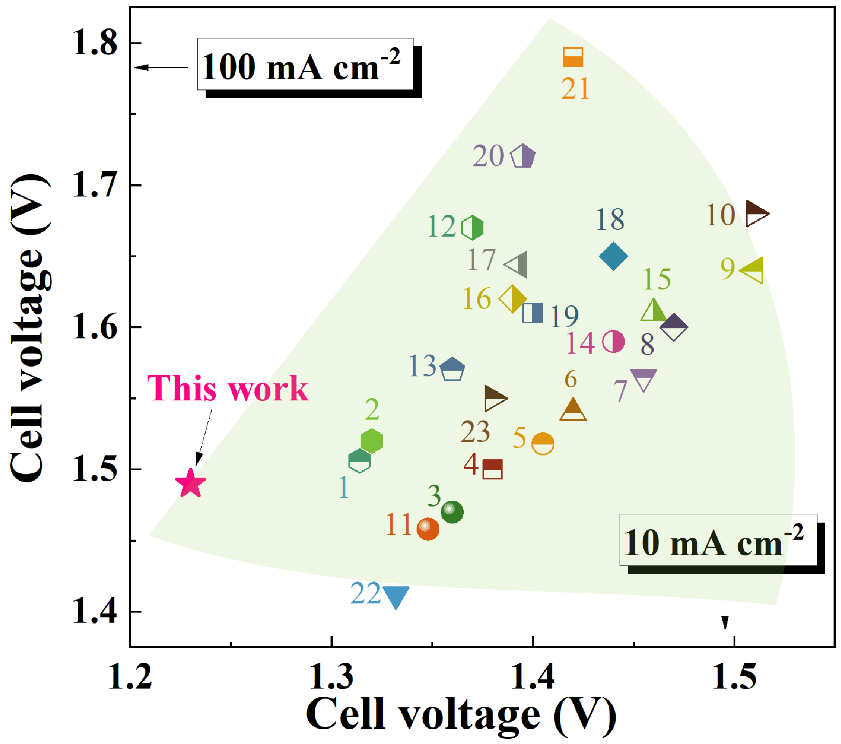


**Figure S65.** The comparisons of cell voltage of the Mo/MoS_V1_ and other bifunctional electrocatalysts reported in the literature at 10 and 100 mA cm^-2^.


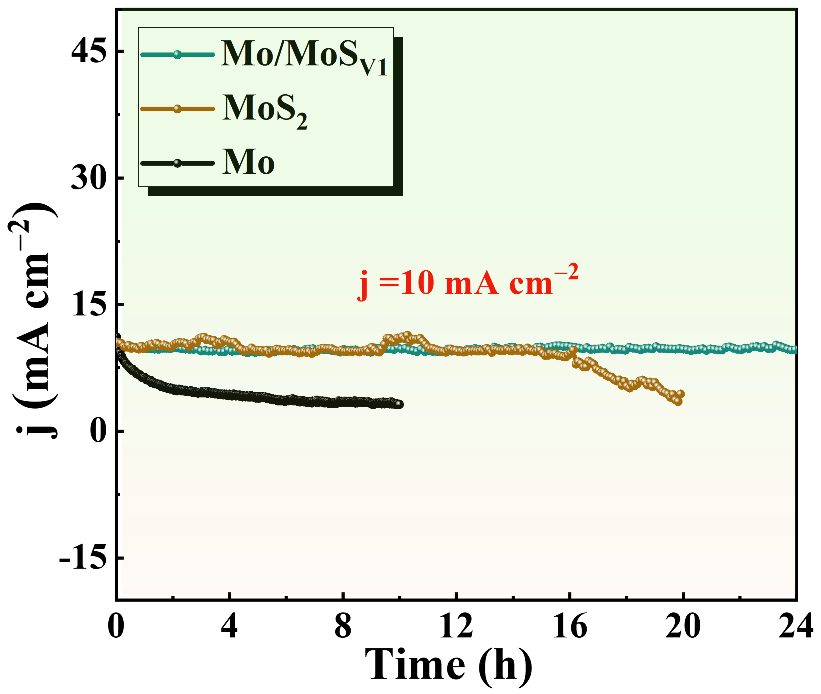


**Figure S66.** Stability tests of the Mo/MoS_V1_ (+,–), MoS_2_ (+,–), and Mo (+,–) cells at 10 mA cm^−2^.


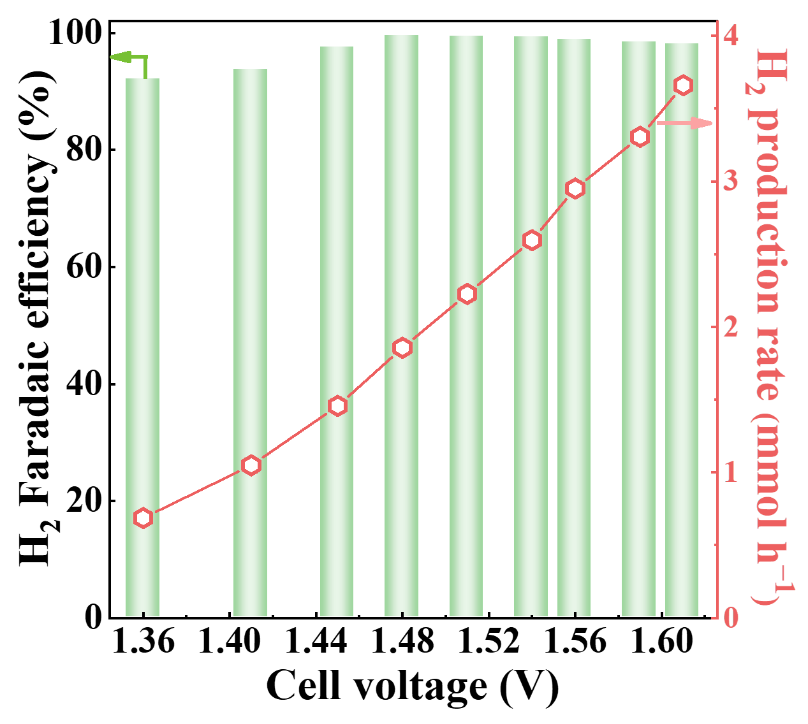


**Figure S67.** Faradaic efficiencies and production rates of H_2_ at different cell voltages in the HER||UOR system.


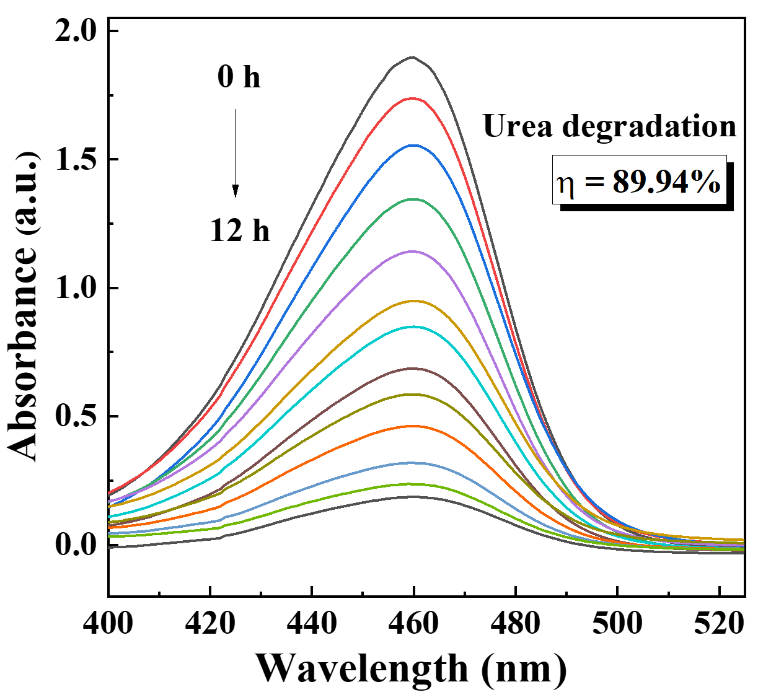


**Figure S68.** Ultraviolet spectroscopy of electrolyte along with the urea degradation from 0 to 12 h.


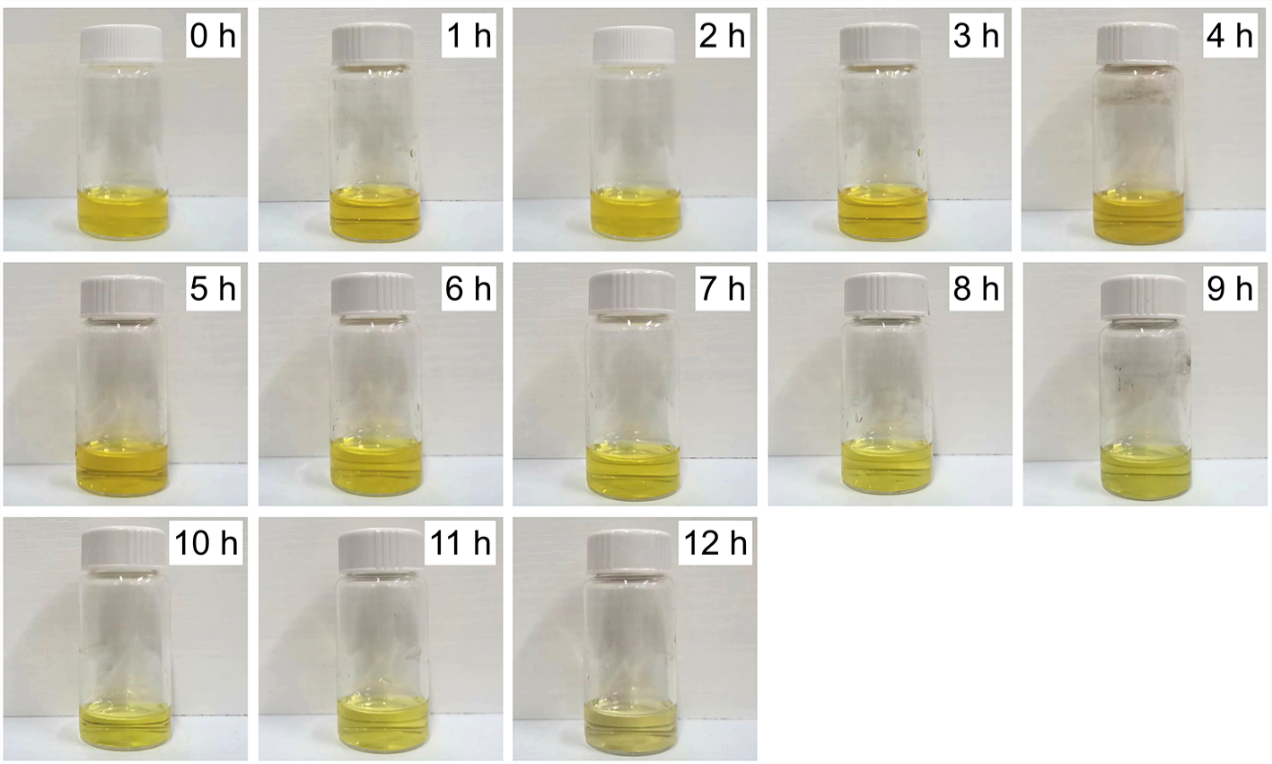
**Figure S69.** Pictures of the urea degradation from 0 to 12 h.


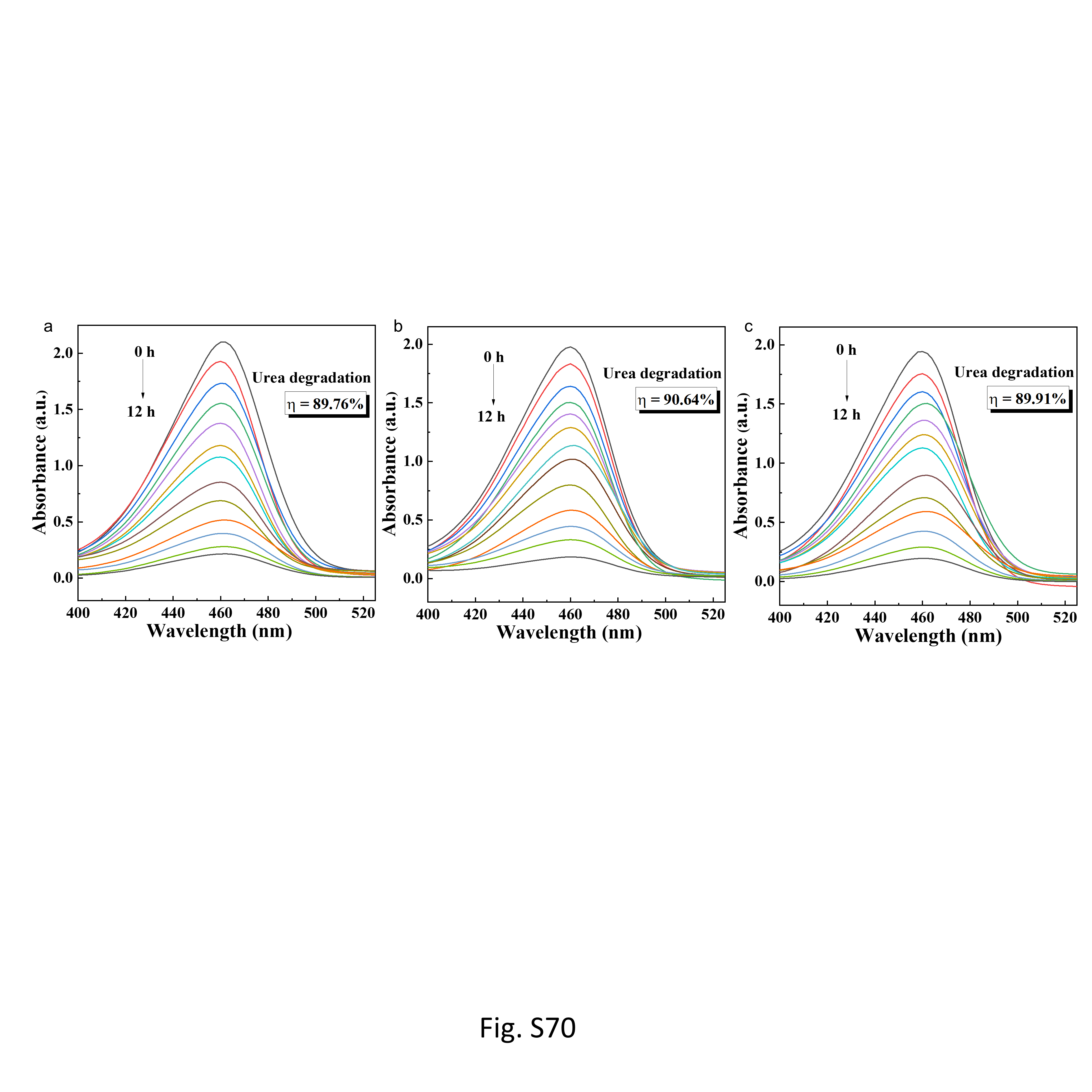


**Figure S70.** a-c) Ultraviolet spectroscopy of electrolyte along with the urea degradation from 0 to 12 h


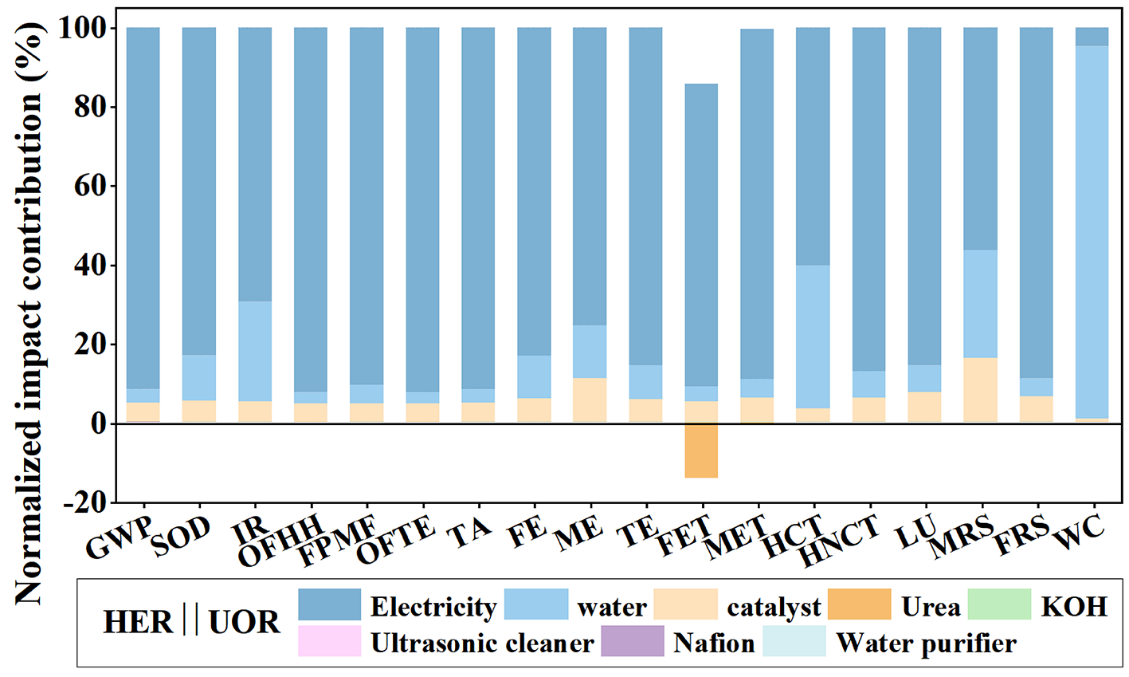


**Figure S71.** Normalized percent impact for all assessed categories when producing 1 kg H_2_ via electrolyzing simulated urea wastewater.


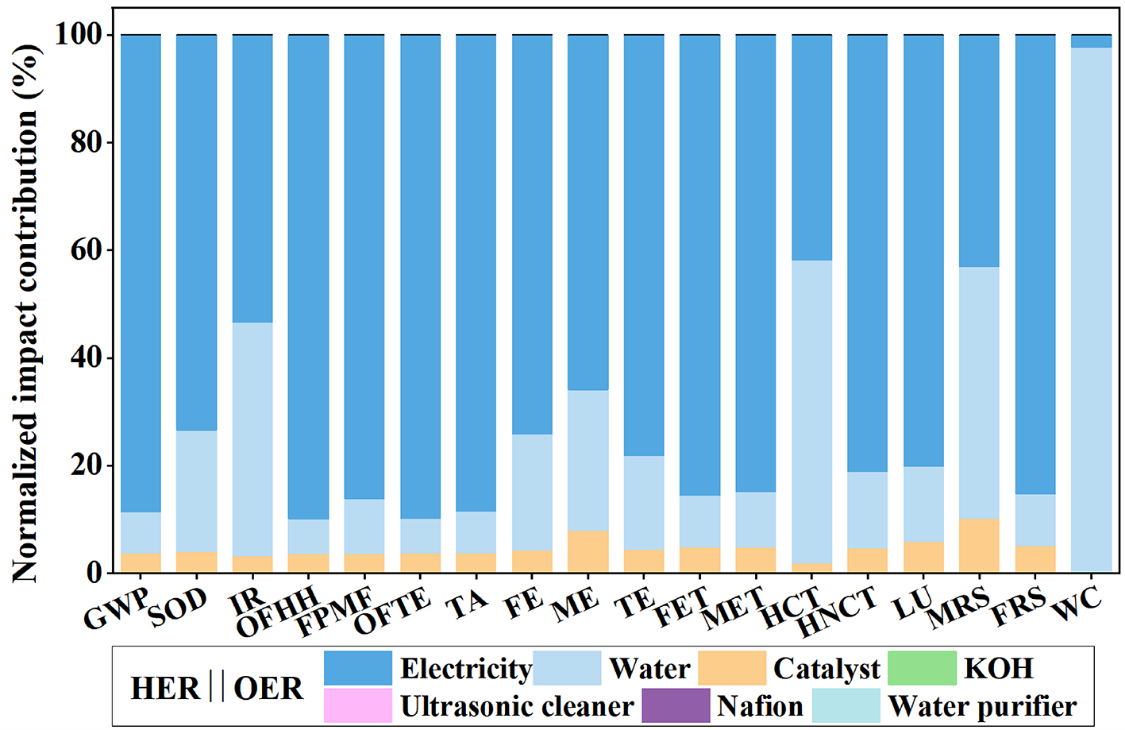


**Figure S72.** Normalized percent impact for all assessed categories when producing 1 kg H_2_ via electrolyzing fresh water.


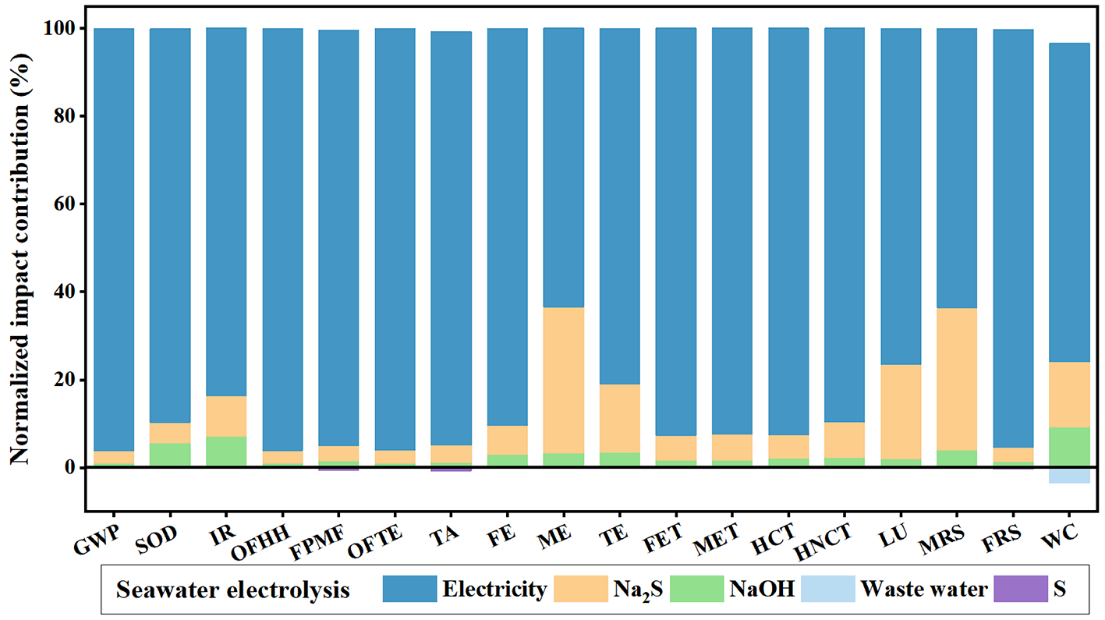


**Figure S73.** Normalized percent impact for all assessed categories when producing 1 kg H_2_ via electrolyzing seawater.

**Supplementary Tables**

**Table S1.** Structure parameters of the Mo/MoS_Vn_ extracted from the EXAFS fitting of Mo K-edge. (S_0_^2^=0.80)

| Sample | Bond type | CN | R (Å) | σ^2^ (Å^2^) | ΔE_0_ (eV) | R factor |
| --- | --- | --- | --- | --- | --- | --- |
| Mo/MoS_2_ | Mo-S | 2.9 | 2.39 | 0.0044 | –1.35 | 0.009 |
| Mo/MoS_V1_ | Mo-S | 2.6 | 2.39 | 0.0045 | –2.75 | 0.009 |
| Mo/MoS_V2_ | Mo-S | 1.4 | 2.38 | 0.0039 | –4.72 | 0.008 |

Note: S_0_^2^ is the amplitude reduction factor; CN is coordination number; R is interatomic distance (the bond length between central atoms and surrounding coordination atoms); σ^2^ is Debye-Waller factor (a measure of thermal and static disorder in absorber-scatterer distances); ΔE_0_ is edge-energy shift (the difference between the zero kinetic energy value of the sample and that of the theoretical model). R factor is used to value the goodness of the fitting.

**Table S2.** The calculated values of charge transfer for different atoms in the Mo/MoS_2_ by Bader charge analysis.

| Mo1 | Mo2 | Mo3 | Mo4 | Mo5 | Mo6 | Mo7 | Mo8 | Mo9 |
| --- | --- | --- | --- | --- | --- | --- | --- | --- |
| -1.06424 | -1.064043 | -1.063807 | -0.457082 | -0.455263 | -0.456905 | -1.004718 | -1.004352 | -1.005706 |
| Mo10 | **Mo11** | **Mo12** | **Mo13** | **Mo14** | **Mo15** | **Mo16** | **Mo17** | **Mo18** |
| -0.451195 | -0.011257 | 0.002991 | -0.443633 | -0.451248 | -0.011359 | 0.002642 | -0.444457 | -0.451195 |
| Mo19 | **Mo20** | **Mo21** | **Mo22** | **Mo23** | **Mo24** | **Mo25** | **Mo26** | **Mo27** |
| -0.01043 | 0.001885 | -0.444067 | -0.007093 | -0.027509 | -0.006698 | -0.027505 | -0.005092 | -0.027203 |
| S1 | **S2** | **S3** | **S4** | **S5** | **S6** | **S7** | **S8** | **S9** |
| 0.650177 | 0.649717 | 0.654743 | 0.648994 | 0.654193 | 0.55983 | 0.560445 | 0.55959 | 0.560353 |
| S10 | **S11** | **S12** | **S13** | **S14** | **S15** | **S16** | **S17** | **S18** |
| 0.560224 | 0.562199 | 0.530919 | 0.507177 | 0.530621 | 0.506996 | 0.531492 | 0.507009 | 0.653868 |

**Table S3.** The calculated values of charge transfer for different atoms in the Mo/MoS_V1_ by Bader charge analysis.

| Mo1 | Mo2 | Mo3 | Mo4 | Mo5 | Mo6 | Mo7 | Mo8 | Mo9 |
| --- | --- | --- | --- | --- | --- | --- | --- | --- |
| -0.9536 | -1.04006 | -1.02503 | -0.44676 | -0.451 | -0.44494 | -0.99357 | -1.03376 | -1.00875 |
| Mo10 | **Mo11** | **Mo12** | **Mo13** | **Mo14** | **Mo15** | **Mo16** | **Mo17** | **Mo18** |
| -0.44188 | -0.00839 | -0.02555 | -0.27169 | -0.37117 | -0.00158 | -0.01375 | -0.25894 | -0.46598 |
| Mo19 | **Mo20** | **Mo21** | **Mo22** | **Mo23** | **Mo24** | **Mo25** | **Mo26** | **Mo27** |
| -0.02608 | 0.010004 | -0.45534 | -0.02228 | -0.02095 | -0.01624 | -0.00925 | -0.02418 | 0.005446 |
| S1 | **S2** | **S3** | **S4** | **S5** | **S6** | **S7** | **S8** | **S9** |
| 0.646681 | 0.656202 | 0.684607 | 0.639668 | 0.671328 | 0.574588 | 0.559645 | 0.564252 | 0.584233 |
| S10 | **S11** | **S12** | **S13** | **S14** | **S15** | **S16** | **S17** | **S18** |
| 0.572984 | 0.569308 | 0.494686 | 0.519477 | 0.515845 | 0.513993 | 0.484484 | 0.563266 | / |

**Table S4.** The calculated values of charge transfer for different atoms in the Mo/MoS_V2_ by Bader charge analysis.

| Mo1 | Mo2 | Mo3 | Mo4 | Mo5 | Mo6 | Mo7 | Mo8 | Mo9 |
| --- | --- | --- | --- | --- | --- | --- | --- | --- |
| -0.95304 | -0.92342 | -1.01263 | -0.44805 | -0.4526 | -0.44417 | -0.99442 | -1.03155 | -1.00901 |
| Mo10 | **Mo11** | **Mo12** | **Mo13** | **Mo14** | **Mo15** | **Mo16** | **Mo17** | **Mo18** |
| -0.24731 | -0.01877 | -0.03613 | -0.29188 | -0.367 | -0.00235 | -0.03019 | -0.27519 | -0.27128 |
| Mo19 | **Mo20** | **Mo21** | **Mo22** | **Mo23** | **Mo24** | **Mo25** | **Mo26** | **Mo27** |
| -0.03604 | 0.013055 | -0.40863 | -0.02205 | -0.01943 | -0.01318 | -0.01434 | -0.02091 | 0.012376 |
| S1 | **S2** | **S3** | **S4** | **S5** | **S6** | **S7** | **S8** | **S9** |
| 0.697438 | 0.690848 | 0.685004 | 0.673916 | 0.574419 | 0.558561 | 0.564213 | 0.583042 | 0.573032 |
| S10 | **S11** | **S12** | **S13** | **S14** | **S15** | **S16** | **S17** | **S18** |
| 0.569697 | 0.521938 | 0.508997 | 0.562009 | 0.505479 | 0.483323 | 0.566198 | / | / |

**Table S5.** Comparison of the cell voltages in the HER||UOR systems between the Mo/MoS_V1_ and recently reported urea electrolysis catalysts.

| Catalysts | V_10_/(V at 10 mA cm^-2^ ) | V_100_/(V at 100 mA cm^-2^ ) | References |
| --- | --- | --- | --- |
| MoS_2_/Ni_3_S_2_ | 1.44 | 1.59 | *J. Colloid Interf. Sci.***2022***,* *628*, 446 |
| NiS/MoS_2_@CC | 1.46 | 1.61 | *Chem. Eng. J.* **2022**, *443*, 136321 |
| Mo-NiS/CFP | 1.51 | 1.64 | *Adv. Funct. Mater.* **2022**, *33*, 2210656 |
| NiCoP | 1.36 | 1.57 | *Small* **2022**, *18*, 2205547 |
| N-Co_9_S_8_/Ni_3_S_2_/NF | 1.40 | 1.61 | *Small* **2023**, *19*, 2207425 |
| Ni_3_N/rGO@NF-350 | 1.405 | 1.518 | *Nanomaterials* **2019**, *9*, 1583 |
| Ni-S-Se/NF | 1.47 | 1.60 | *Nano Energy* **2021**, *81*, 105605 |
| Rh/NiV-LDH | 1.336 | 1.47 | *Sci. Bull.* **2022**, *67*, 1763 |
| V-Ni_3_N | 1.42 | 1.54 | *J. Mater. Chem. A* **2021***, 9*, 4159 |
| Ni_2_Fe(CN)_6_/NF | 1.38 | 1.50 | *Nature Energy* **2021**, *6*, 904 |
| NiFeRh-LDH | 1.455 | 1.565 | *Appl. Catal. B-Environ.* **2021**, *284*, 119740 |
| CoMn/CoMn_2_O_4_ | 1.51 | 1.68 | *Adv. Funct. Mater.* **2020**, *30*, 2000556 |
| NiMoO_4_/NF | 1.38 | 1.55 | *Energ. Environ. Sci.* **2018**, *11*, 1890 |
| CoO−Co_4_N@NiFe-LDH | 1.393 | 1.644 | *ACS Sustainable Chem. Eng.* **2021**, *9*, 14180 |
| Ni_3_N/Ni_0.2_Mo_0.8_N/NF | 1.348 | 1.458 | *Chem. Eng. J.* **2021**, *409*, 128240 |
| Fe-Co_0.85_Se/FeCo LDH | 1.32 | 1.52 | *Adv. Funct. Mater.* **2023**, *33*, 2212811 |
| Pt-NiS@Ni-CNFs | 1.44 | 1.65 | *Small* **2024**, *20*, 2304782 |
| NiMoO_4_ NC_400_/NT | 1.37 | 1.67 | *Chem. Eng. J.* **2023**, *471*, 144657 |
| Ni–Mn–P@Ni–Co | 1.395 | 1.720 | *Int. J. Hydrogen Energ.* **2024**, *51*, 1022 |
| V/meso-Co/NF-5 | 1.39 | 1.62 | *Environ. Res.* **2023**, *236*, 116818 |
| Ni–P/CNTs/NF | 1.42 | 1.79 | *Appl. Surf. Sci.* **2024**, *645*, 158831 |
| ZnS@Co_9_S_8_@Ni_3_S_2_-1/2 | 1.314 | 1.506 | *Green Energy Environ.* **2023**, *8*, 798 |
| Ni(OH)_2_/ACE | 1.332 | 1.412 | *Adv. Mater.* **2024***,* 2409292 |
| Mo/MoS_V1_ | **1.23** | **1.49** | **This work** |

**Table S6.** Comparison of life cycle assessment for the three hydrogen production technologies.

| Category | Unit | HER\|\|UOR | HER\|\|OER | Seawater electrolysis |
| --- | --- | --- | --- | --- |
| global warming potential (GWP) | kg CO_2_ eq | 68.29886982 | 87.73346456 | 100 |
| stratospheric ozone depletion (SOD) | kg CFC11 eq | 70.33546432 | 98.82889838 | 100 |
| ionizing radiation (IR) | kBq Co-60 eq | 61.81192803 | 100 | 78.91970941 |
| ozone formation human health (OFHH) | kg NO_x_ eq | 67.7007672 | 86.38067866 | 100 |
| fine particulate matter formation (FPMF) | kg PM_2.5_ eq | 68.66397193 | 89.57557069 | 100 |
| ozone formation terrestrial ecosystems (OFTE) | kg NO_x_ eq | 67.74195849 | 86.47975644 | 100 |
| terrestrial acidification (TA) | kg SO_2_ eq | 67.97735111 | 87.35293737 | 100 |
| freshwater eutrophication (FE) | kg P eq | 70.79080569 | 98.72797799 | 100 |
| marine eutrophication (ME) | kg N eq | 54.62217617 | 77.65977741 | 100 |
| terrestrial ecotoxicity (TE) | kg 1,4-DCB | 61.61616848 | 83.76631061 | 100 |
| freshwater ecotoxicity (FET) | kg 1,4-DCB | 56.36040097 | 87.57455033 | 100 |
| marine ecotoxicity (MET) | kg 1,4-DCB | 67.35970475 | 87.95295307 | 100 |
| human carcinogenic toxicity (HCT) | kg 1,4-DCB | 55.90675374 | 100 | 55.96460246 |
| human non-carcinogenic toxicity (HNCT) | kg 1,4-DCB | 67.0132225 | 89.27780347 | 100 |
| land use (LU) | m^2^a crop eq | 58.18678282 | 77.21049515 | 100 |
| mineral resource scarcity (MRS) | kg Cu eq | 61.50641668 | 100 | 83.77671928 |
| fossil resource scarcity (FRS) | kg oil eq | 70.02935423 | 90.78438282 | 100 |
| water consumption (WC) | m^3^ | 37.24154638 | 100 | 3.53764202 |

**Table S7.** The contribution ratio of the various contributing factors of HER||UOR.

| Category | Unit | Total | KOH | Urea | Nafion | Catalyst | Water | Ultrasonic cleaner | Electricity | Water purifier |
| --- | --- | --- | --- | --- | --- | --- | --- | --- | --- | --- |
| GWP | kg CO_2_ eq | 52.639069 | 0.008622196 | 0 | 0.00041409 | 2.5311031 | 1.853286 | 2.92E-05 | 48.23612 | 0.009498 |
| SOD | kg CFC11 eq | 1.22E-05 | 3.35E-09 | 0 | 9.66E-10 | 6.68E-07 | 1.40E-06 | 6.16E-12 | 1.02E-05 | 2.00E-09 |
| IR | kBq Co-60 eq | 1.0981025 | 0.000507071 | 0 | 3.55E-05 | 0.056356916 | 0.277811 | 4.62E-07 | 0.763242 | 0.00015 |
| OFHH | kg NO_x_ eq | 0.14483063 | 2.23E-05 | 0 | 4.72E-07 | 0.006708259 | 0.00425 | 8.11E-08 | 0.133823 | 2.63E-05 |
| FPMF | kg PM_2.5_ eq | 0.08238784 | 1.77E-05 | 0 | 5.33E-07 | 0.003863871 | 0.003963 | 4.51E-08 | 0.074528 | 1.47E-05 |
| OFTE | kg NO_x_ eq | 0.1453119 | 2.26E-05 | 0 | 4.81E-07 | 0.006808744 | 0.004341 | 8.12E-08 | 0.134113 | 2.64E-05 |
| TA | kg SO_2_ eq | 0.18259399 | 3.31E-05 | 0 | 1.27E-06 | 0.008886966 | 0.006502 | 1.01E-07 | 0.167137 | 3.29E-05 |
| FE | kg P eq | 0.011197999 | 3.78E-06 | 0 | 1.21E-07 | 0.000664185 | 0.001217 | 5.64E-09 | 0.009311 | 1.83E-06 |
| ME | kg N eq | 0.000768792 | 2.37E-07 | 0 | 8.86E-09 | 8.54E-05 | 0.000103 | 3.51E-10 | 0.00058 | 1.14E-07 |
| TE | kg 1,4-DCB | 66.617405 | 0.04219317 | -0.00013158 | 0.00158553 | 3.870273 | 5.696082 | 3.45E-05 | 56.99615 | 0.011222 |
| FET | kg 1,4-DCB | 1.6399444 | 0.000482128 | -0.32538 | 1.73E-05 | 0.12088174 | 0.088399 | 1.06E-06 | 1.755197 | 0.000346 |
| MET | kg 1,4-DCB | 2.4778132 | 0.000635586 | -7.75E-03 | 2.28E-05 | 0.15305339 | 0.120044 | 1.34E-06 | 2.211372 | 0.000435 |
| HCT | kg 1,4-DCB | 2.7450484 | 0.00056877 | 0 | 1.55E-05 | 0.094952732 | 0.994253 | 1.00E-06 | 1.654931 | 0.000326 |
| HNCT | kg 1,4-DCB | 30.17494 | 0.01024916 | 0 | 0.00037561 | 1.8503115 | 2.05832 | 1.59E-05 | 26.2505 | 0.005169 |
| LU | m^2^a crop eq | 0.68587902 | 0.000264253 | 0 | 5.61E-06 | 0.052364285 | 0.04591 | 3.56E-07 | 0.587219 | 0.000116 |
| MRS | kg Cu eq | 0.054668947 | 3.59E-05 | 0 | 1.14E-06 | 0.008826193 | 0.014983 | 1.87E-08 | 0.030816 | 6.07E-06 |
| FRS | kg oil eq | 10.537006 | 0.002285647 | 0 | 5.84E-05 | 0.68856469 | 0.478499 | 5.67E-06 | 9.365748 | 0.001844 |
| WC | m^3^ | 2.5458895 | 7.84E-05 | 0 | 7.01E-06 | 0.021788414 | 2.401612 | 7.41E-08 | 0.12238 | 2.41E-05 |

**Table S8.** The contribution ratio of the various contributing factors of HER||OER.

| **Category** | **Unit** | **Total** | **KOH** | **Nafion** | **Catalyst** | **Water** | **Ultrasonic cleaner** | **Electricity** | **Water purifier** |
| --- | --- | --- | --- | --- | --- | --- | --- | --- | --- |
| GWP | kg CO_2_ eq | 67.617634 | 0.008622196 | 0.004231798 | 2.586691 | 5.140824 | 2.92E-05 | 59.86774 | 0.009498 |
| SOD | kg CFC11 eq | 1.72E-05 | 3.35E-09 | 9.87E-09 | 6.83E-07 | 3.88E-06 | 6.16E-12 | 1.26E-05 | 2.00E-09 |
| IR | kBq Co-60 eq | 1.776522 | 0.000507071 | 0.000362361 | 0.05759462 | 0.77061785 | 4.62E-07 | 0.947289 | 0.00015 |
| OFHH | kg NO_x_ eq | 0.18479213 | 2.23E-05 | 4.82E-06 | 0.00685559 | 0.011790402 | 8.11E-08 | 0.166093 | 2.63E-05 |
| FPMF | kg PM_2.5_ eq | 0.10747904 | 1.77E-05 | 5.45E-06 | 0.00394873 | 0.010992672 | 4.51E-08 | 0.0925 | 1.47E-05 |
| OFTE | kg NO_x_ eq | 0.18550597 | 2.26E-05 | 4.92E-06 | 0.00695828 | 0.012041123 | 8.12E-08 | 0.166453 | 2.64E-05 |
| TA | kg SO_2_ eq | 0.23463876 | 3.31E-05 | 1.30E-05 | 0.00908214 | 0.01803695 | 1.01E-07 | 0.207441 | 3.29E-05 |
| FE | kg P eq | 0.015617223 | 3.78E-06 | 1.24E-06 | 0.00067877 | 0.003374789 | 5.64E-09 | 0.011557 | 1.83E-06 |
| ME | kg N eq | 0.00109304 | 2.37E-07 | 9.05E-08 | 8.73E-05 | 0.00028518 | 3.51E-10 | 0.00072 | 1.14E-07 |
| TE | kg 1,4-DCB | 90.565421 | 0.04219317 | 0.016203475 | 3.9552717 | 15.800342 | 3.45E-05 | 70.74015 | 0.011222 |
| FET | kg 1,4-DCB | 2.5481968 | 0.000482128 | 0.000176423 | 0.12353653 | 0.24521101 | 1.06E-06 | 2.178444 | 0.000346 |
| MET | kg 1,4-DCB | 3.2353317 | 0.000635586 | 0.000232919 | 0.15641474 | 0.33299086 | 1.34E-06 | 2.744621 | 0.000435 |
| HCT | kg 1,4-DCB | 4.910048 | 0.00056877 | 0.000158367 | 0.09703808 | 2.7579557 | 1.00E-06 | 2.054 | 0.000326 |
| HNCT | kg 1,4-DCB | 40.200312 | 0.01024916 | 0.003838636 | 1.8909479 | 5.7095666 | 1.59E-05 | 32.58053 | 0.005169 |
| LU | m^2^a crop eq | 0.9101218 | 0.000264253 | 5.74E-05 | 0.05351431 | 0.12734939 | 3.56E-07 | 0.728821 | 0.000116 |
| MRS | kg Cu eq | 0.088883323 | 3.59E-05 | 1.17E-05 | 0.00902003 | 0.041562375 | 1.87E-08 | 0.038247 | 6.07E-06 |
| FRS | kg oil eq | 13.659923 | 0.002285647 | 0.00059692 | 0.7036869 | 1.327306 | 5.67E-06 | 11.6242 | 0.001844 |
| WC | m^3^ | 6.8361541 | 7.84E-05 | 7.16E-05 | 0.02226693 | 6.6618225 | 7.41E-08 | 0.15189 | 2.41E-05 |

**Table S9.** The contribution ratio of the various contributing factors of seawater electrolysis.

| **Category** | **Unit** | **Total** | **NaOH** | **Na_2_S** | **Electricity** | **S** | **Wastewater** | |
| --- | --- | --- | --- | --- | --- | --- | --- | --- |
| GWP | kg CO_2_ eq | 77.071657 | 0.9016447 | 2.1078884 | 74.093311 | -0.03118669 | | 0 |
| SOD | kg CFC11 eq | 1.74E-05 | 9.80E-07 | 8.19E-07 | 1.56E-05 | -1.17E-08 | | 0 |
| IR | kBq Co-60 eq | 1.402026 | 0.10116692 | 0.12881014 | 1.172381 | -0.00033208 | | 0 |
| OFHH | kg NO_x_ eq | 0.21392763 | 0.002363899 | 0.006083175 | 0.20555898 | -7.84E-05 | | 0 |
| FPMF | kg PM_2.5_ eq | 0.119987 | 0.001992735 | 0.004137978 | 0.11447922 | -0.00062293 | | 0 |
| OFTE | kg NO_x_ eq | 0.21450797 | 0.002387827 | 0.00619935 | 0.2060045 | -8.37E-05 | | 0 |
| TA | kg SO_2_ eq | 0.26861004 | 0.00347546 | 0.010529217 | 0.25673193 | -0.00212656 | | 0 |
| FE | kg P eq | 0.015818437 | 0.000475505 | 0.001044578 | 0.01430289 | -4.54E-06 | | 0 |
| ME | kg N eq | 0.001407472 | 4.68E-05 | 0.000469616 | 0.00089128 | -2.54E-07 | | 0 |
| TE | kg 1,4-DCB | 108.11676 | 3.8700618 | 16.775586 | 87.549194 | -0.07808521 | | 0 |
| FET | kg 1,4-DCB | 2.9097458 | 0.048299799 | 0.16566307 | 2.6960787 | -0.00029572 | | 0 |
| MET | kg 1,4-DCB | 3.6784799 | 0.063776982 | 0.21835671 | 3.3967886 | -0.00044233 | | 0 |
| HCT | kg 1,4-DCB | 2.7478889 | 0.060690698 | 0.14574114 | 2.5420649 | -0.00060787 | | 0 |
| HNCT | kg 1,4-DCB | 45.028339 | 1.1032036 | 3.6131524 | 40.322201 | -0.01021761 | | 0 |
| LU | m^2^a crop eq | 1.178754 | 0.024324507 | 0.25301424 | 0.90200041 | -0.00058514 | | 0 |
| MRS | kg Cu eq | 0.074463533 | 0.003065522 | 0.024094322 | 0.04733537 | -3.17E-05 | | 0 |
| FRS | kg oil eq | 15.046556 | 0.22595864 | 0.4754349 | 14.386301 | -0.04113871 | | 0 |
| WC | m^3^ | 0.24183866 | 0.024235789 | 0.038542043 | 0.18798213 | -8.58E-05 | | -0.008836 |

**Table S10.** Comparisons of the energy consumption for the three hydrogen production technologies.

| **Impact category** | **Unit** | **HER\|\|UOR** | **HER\|\|OER** | **Seawater electrolysis** |
| --- | --- | --- | --- | --- |
| Non-renewable, fossil | MJ | 489.2341 | 633.8047 | 699.093 |
| Non-renewable, nuclear energy | MJ | 20.48641 | 33.18625 | 25.7825 |
| Non-renewable, biomass | MJ | 0.019095 | 0.024446 | 0.005953 |
| Renewable, biomass | MJ | 6.835417 | 9.168026 | 10.28456 |
| Renewable, wind, solar, geothermy | MJ | 8.044836 | 10.98941 | 11.39944 |
| Renewable, water | MJ | 34.38682 | 44.9643 | 49.78389 |

**Table S11.** The simulation results for HER||UOR system using Monte Carlo method for 1000 times.

| **Category** | **Unit** | **Average** | **Median** | **SD** | **CV** | **2.5% (U)** | **97.5% (V)** | **SEM** |
| --- | --- | --- | --- | --- | --- | --- | --- | --- |
| FPMF | kg PM_2.5_ eq | 8.17E-02 | 7.94E-02 | 1.41E-02 | 1.72E+01 | 6.11E-02 | 1.15E-01 | 4.44E-04 |
| FRS | kg oil eq | 1.05E+01 | 1.03E+01 | 1.54E+00 | 1.46E+01 | 8.05E+00 | 1.40E+01 | 4.87E-02 |
| FET | kg 1,4-DCB | 1.63E+00 | 1.52E+00 | 6.02E-01 | 3.68E+01 | 7.47E-01 | 3.05E+00 | 1.90E-02 |
| FE | kg P eq | 1.14E-02 | 9.28E-03 | 7.74E-03 | 6.81E+01 | 4.00E-03 | 2.99E-02 | 2.45E-04 |
| GWP | kg CO_2_ eq | 5.24E+01 | 5.15E+01 | 7.75E+00 | 1.48E+01 | 3.89E+01 | 6.97E+01 | 2.45E-01 |
| HCT | kg 1,4-DCB | 2.76E+00 | 2.33E+00 | 1.70E+00 | 6.15E+01 | 1.36E+00 | 6.77E+00 | 5.37E-02 |
| HNCT | kg 1,4-DCB | 3.00E+01 | 2.54E+01 | 1.85E+01 | 6.15E+01 | 1.21E+01 | 7.72E+01 | 5.84E-01 |
| IR | kBq Co-60 eq | 1.07E+00 | 6.14E-01 | 1.38E+00 | 1.29E+02 | 1.50E-01 | 5.58E+00 | 4.37E-02 |
| LU | m^2^a crop eq | 6.73E-01 | 6.11E-01 | 2.81E-01 | 4.18E+01 | 3.70E-01 | 1.36E+00 | 8.88E-03 |
| MET | kg 1,4-DCB | 2.47E+00 | 2.33E+00 | 7.63E-01 | 3.09E+01 | 1.37E+00 | 4.27E+00 | 2.41E-02 |
| ME | kg N eq | 7.66E-04 | 7.50E-04 | 1.10E-04 | 1.43E+01 | 5.84E-04 | 1.02E-03 | 3.47E-06 |
| MRS | kg Cu eq | 5.45E-02 | 5.37E-02 | 7.34E-03 | 1.35E+01 | 4.22E-02 | 7.09E-02 | 2.32E-04 |
| OFHH | kg NO_x_ eq | 1.44E-01 | 1.43E-01 | 1.96E-02 | 1.36E+01 | 1.12E-01 | 1.89E-01 | 6.19E-04 |
| OFTE | kg NO_x_ eq | 1.45E-01 | 1.43E-01 | 1.96E-02 | 1.36E+01 | 1.12E-01 | 1.89E-01 | 6.21E-04 |
| SOD | kg CFC11 eq | 1.22E-05 | 1.19E-05 | 2.30E-06 | 1.88E+01 | 8.91E-06 | 1.73E-05 | 7.28E-08 |
| TA | kg SO_2_ eq | 1.82E-01 | 1.80E-01 | 2.43E-02 | 1.33E+01 | 1.42E-01 | 2.40E-01 | 7.68E-04 |
| TE | kg 1,4-DCB | 6.61E+01 | 6.20E+01 | 1.86E+01 | 2.81E+01 | 4.23E+01 | 1.14E+02 | 5.87E-01 |
| WC | m^3^ | 2.14E+00 | 2.30E+00 | 2.98E+01 | 1.40E+03 | -6.16E+01 | 5.69E+01 | 9.44E-01 |

**Table S12.** The simulation results for HER||OER system using Monte Carlo method for 1000 times.

| **Category** | **Unit** | **Average** | **Median** | **SD** | **CV** | **2.5% (U)** | **97.5% (V)** | **SEM** |
| --- | --- | --- | --- | --- | --- | --- | --- | --- |
| FPMF | kg PM_2.5_ eq | 1.08E-01 | 1.05E-01 | 1.86E-02 | 1.73E+01 | 7.97E-02 | 1.54E-01 | 5.88E-04 |
| FRS | kg oil eq | 1.37E+01 | 1.35E+01 | 1.84E+00 | 1.34E+01 | 1.04E+01 | 1.76E+01 | 5.81E-02 |
| FET | kg 1,4-DCB | 2.52E+00 | 2.43E+00 | 7.22E-01 | 2.86E+01 | 1.42E+00 | 4.26E+00 | 2.28E-02 |
| FE | kg P eq | 1.57E-02 | 1.31E-02 | 8.68E-03 | 5.53E+01 | 6.30E-03 | 3.87E-02 | 2.75E-04 |
| GWP | kg CO_2_ eq | 6.77E+01 | 6.72E+01 | 9.63E+00 | 1.42E+01 | 5.10E+01 | 8.84E+01 | 3.05E-01 |
| HCT | kg 1,4-DCB | 5.03E+00 | 4.41E+00 | 4.91E+00 | 9.75E+01 | 2.40E+00 | 1.02E+01 | 1.55E-01 |
| HNCT | kg 1,4-DCB | 3.88E+01 | 3.28E+01 | 2.89E+01 | 7.43E+01 | 1.71E+01 | 9.94E+01 | 9.13E-01 |
| IR | kBq Co-60 eq | 1.78E+00 | 1.07E+00 | 2.40E+00 | 1.35E+02 | 2.34E-01 | 8.26E+00 | 7.58E-02 |
| LU | m^2^a crop eq | 8.94E-01 | 8.17E-01 | 3.59E-01 | 4.01E+01 | 5.16E-01 | 1.73E+00 | 1.13E-02 |
| MET | kg 1,4-DCB | 3.20E+00 | 3.09E+00 | 9.12E-01 | 2.85E+01 | 1.83E+00 | 5.39E+00 | 2.88E-02 |
| ME | kg N eq | 1.09E-03 | 1.08E-03 | 1.28E-04 | 1.18E+01 | 8.64E-04 | 1.37E-03 | 4.05E-06 |
| MRS | kg Cu eq | 8.86E-02 | 8.76E-02 | 1.16E-02 | 1.31E+01 | 6.98E-02 | 1.17E-01 | 3.68E-04 |
| OFHH | kg NO_x_ eq | 1.85E-01 | 1.84E-01 | 2.34E-02 | 1.26E+01 | 1.43E-01 | 2.35E-01 | 7.39E-04 |
| OFTE | kg NO_x_ eq | 1.86E-01 | 1.85E-01 | 2.34E-02 | 1.26E+01 | 1.44E-01 | 2.36E-01 | 7.41E-04 |
| SOD | kg CFC11 eq | 1.72E-05 | 1.68E-05 | 2.83E-06 | 1.64E+01 | 1.32E-05 | 2.44E-05 | 8.94E-08 |
| TA | kg SO_2_ eq | 2.35E-01 | 2.33E-01 | 2.85E-02 | 1.21E+01 | 1.84E-01 | 2.97E-01 | 9.01E-04 |
| TE | kg 1,4-DCB | 9.06E+01 | 8.48E+01 | 2.56E+01 | 2.82E+01 | 5.89E+01 | 1.60E+02 | 8.09E-01 |
| WC | m^3^ | 7.83E+00 | 1.02E+01 | 3.92E+01 | 5.01E+02 | -7.59E+01 | 8.05E+01 | 1.24E+00 |

**Table S13.** The simulation results for seawater electrolysis using Monte Carlo method for 1000 times.

| **Category** | **Unit** | **Average** | **Median** | **SD** | **CV** | **2.5% (U)** | **97.5% (V)** | **SEM** |
| --- | --- | --- | --- | --- | --- | --- | --- | --- |
| FPMF | kg PM_2.5_ eq | 1.20E-01 | 1.17E-01 | 2.15E-02 | 1.80E+01 | 8.67E-02 | 1.70E-01 | 6.81E-04 |
| FRS | kg oil eq | 1.51E+01 | 1.50E+01 | 2.28E+00 | 1.51E+01 | 1.11E+01 | 1.99E+01 | 7.20E-02 |
| FET | kg 1,4-DCB | 2.89E+00 | 2.70E+00 | 9.49E-01 | 3.28E+01 | 1.55E+00 | 5.13E+00 | 3.00E-02 |
| FE | kg P eq | 1.62E-02 | 1.34E-02 | 1.07E-02 | 6.59E+01 | 5.73E-03 | 4.45E-02 | 3.38E-04 |
| GWP | kg CO_2_ eq | 7.74E+01 | 7.66E+01 | 1.17E+01 | 1.51E+01 | 5.63E+01 | 1.03E+02 | 3.69E-01 |
| HCT | kg 1,4-DCB | 2.79E+00 | 2.17E+00 | 3.07E+00 | 1.10E+02 | 1.13E+00 | 7.68E+00 | 9.70E-02 |
| HNCT | kg 1,4-DCB | 5.10E+01 | 3.70E+01 | 2.05E+02 | 4.02E+02 | 1.87E+01 | 1.11E+02 | 6.48E+00 |
| IR | kBq Co-60 eq | 1.34E+00 | 7.81E-01 | 1.75E+00 | 1.30E+02 | 1.72E-01 | 5.74E+00 | 5.52E-02 |
| LU | m^2^a crop eq | 1.19E+00 | 1.07E+00 | 4.62E-01 | 3.89E+01 | 6.87E-01 | 2.53E+00 | 1.46E-02 |
| MET | kg 1,4-DCB | 3.65E+00 | 3.41E+00 | 1.21E+00 | 3.30E+01 | 1.97E+00 | 6.40E+00 | 3.81E-02 |
| ME | kg N eq | 1.41E-03 | 1.40E-03 | 1.55E-04 | 1.11E+01 | 1.13E-03 | 1.76E-03 | 4.91E-06 |
| MRS | kg Cu eq | 7.45E-02 | 7.36E-02 | 9.56E-03 | 1.28E+01 | 5.75E-02 | 9.47E-02 | 3.02E-04 |
| OFHH | kg NO_x_ eq | 2.14E-01 | 2.13E-01 | 2.84E-02 | 1.33E+01 | 1.61E-01 | 2.72E-01 | 9.00E-04 |
| OFTE | kg NO_x_ eq | 2.15E-01 | 2.13E-01 | 2.85E-02 | 1.33E+01 | 1.62E-01 | 2.73E-01 | 9.02E-04 |
| SOD | kg CFC11 eq | 1.74E-05 | 1.70E-05 | 3.22E-06 | 1.85E+01 | 1.25E-05 | 2.54E-05 | 1.02E-07 |
| TA | kg SO_2_ eq | 2.69E-01 | 2.67E-01 | 3.58E-02 | 1.33E+01 | 2.05E-01 | 3.48E-01 | 1.13E-03 |
| TE | kg 1,4-DCB | 1.07E+02 | 1.00E+02 | 3.22E+01 | 3.02E+01 | 7.01E+01 | 1.84E+02 | 1.02E+00 |
| WC | m^3^ | 1.02E+00 | 3.04E+00 | 4.38E+01 | 4.29E+03 | -9.12E+01 | 8.42E+01 | 1.38E+00 |

**Supplementary References**

[1] Y. F. Ren, C. T. Wang, W. Duan, L. H. Zhou, X. Pang, D. J. Wang, Y. Z. Zhen, C. M. Yang, Z. W. Gao, *J. Colloid Interface Sci.* **2022**, *628*, 446.

[2] C. J. Gu, G. Y. Zhou, J. Yang, H. Pang, M. Y. Zhang, Q. Zhao, X. F. Gu, S. Tian, J. B. Zhang, L. Xu, Y. W. Tang, *Chem. Eng. J.* **2022**, *443*, 136321.

[3] Y. Zhou, Y. M. Wang, D. Q. Kong, Q. Zhao, L. Zhao, J. L. Zhang, X. X. Chen, Y. N. Li, Y. Xu, C. Meng, *Adv. Funct. Mater.* **2023**, *33*, 2210656.

[4] X. D. Ding, L. S. Pei, Y. X. Huang, D. Y. Chen, Z. L. Xie, *Small* **2022**, *18*, 2205547.

[5] H. Xie, Y. F. Feng, X. Y. He, Y. Zhu, Z. Y. Li, H. Liu, S. Y. Zeng, Q. Z. Qian, G. Q. Zhang, *Small* **2023**, *19*, 2207425.

[6] F. Wang, D. S. Zhao, L. B. Zhang, L. M. Fan, X. T. Zhang, S. N. Hu, *Nanomaterials* **2019**, *9*, 1583.

[7] N. Chen, Y. X. Du, G. Zhang, W. T. Lu, F. Cao, *Nano Energy* **2021**, *81*, 105605.

[8] H. C. Sun, L. F. Li, H. C. Chen, D. L. Duan, M. Humayun, Y. Qiu, X. Zhang, X. Ao, Y. Wu, Y. J. Pang, K. F. Huo, C. D. Wang, Y. J. Xiong, *Sci. Bull.* **2022**, *67*, 1763.

[9] R. Q. Li, Q. Liu, Y. N. Zhou, M. J. Lu, J. L. Hou, K. G. Qu, Y. C. Zhu, O. Fontaine, *J. Mater. Chem. A* **2021**, *9*, 4159.

[10] S. K. Geng, J. Hu, H. B. Shu, P. Chen, Y. Zheng, S. Z. Qiao, S. Q. Li, H. Su, X. Zhao, Q. H. Liu, M. Jaroniec, *Nat. Energy* **2021**, *6*, 904.

[11] H. C. Sun, W. Zhang, J. G. Li, Z. S. Li, X. Ao, K. H. Xue, K. Ostrikov, J. Tang, C. D. Wang, *Appl. Catal-B Environ*. **2021**, *284*, 119740.

[12] C. Wang, H. L. Lu, Z. Y. Mao, C. L. Yan, G. Z. Shen, X. F. Wang, *Adv. Funct. Mater.* **2020**, *30*, 2000556.

[13] Z. Y. Yu, C. Lang, M. R. Gao, Y. Chen, Q. Fu, Y. Duan, S. H. Yu, *Energ. Environ. Sci.* **2018**, *11*, 1890.

[14] A. J. Chen, M. Humayun, Y. D. Li, H. M. Zhang, H. C. Sun, Y. Wu, C. D. Wang, *ACS Sustainable Chem. Eng.* **2021**, *9*, 14180.

[15] R. Q. Li, X. Y. Wan, B. L. Chen, R. Y. Cao, Q. H. Ji, J. Deng, K. G. Qu, X. B. Wang, Y. C. Zhu, *Chem. Eng. J.* **2021**, *409*, 128240.

[16] H. Z. Yu, S. Q. Zhu, Y. X. Hao, Yu. M. Chang, L. Li, J. Ma, H. Y. Chen, M. H. Shao, S. J. Peng, *Adv. Funct. Mater.* **2023**, *33*, 2212811.

[17] M. X. Zhong, J. Y. Yang, M. J. Xu, S. Y. Ren, X. J. Chen, C. Wang, M. B. Gao, X. F. Lu, *Small* **2024**, *20*, 2304782.

[18] J. R. Zeng, W. H. Chen, G. W. Zhang, S. H. Yang, L. Yu, X. Cao, H. Chen, Y. Liu, L. J. Song, Y. J. Qiu, *Chem. Eng. J.* **2023**, *471*, 144657.

[19] B. J. Zhou, Y. K. Shao, Z. G. Li, W. S. Yang, X. N. Ren, Y. D. Hao, *Inter. J. Hydrogen Energy* **2024**, *51*, 1022.

[20] M. S. Amer, P. Arunachalam, A. M Al-Mayouf, A. A AlSaleh, Z. A Almutairi, *Environ. Research* **2023**, *236*, 116818.

[21] T. H. Wu, Z. T. Qiu, C. N. Hsieh, *Appl. Surface Sci.* **2024**, *645*, 158831.

[22] X. Q. Du, Y. Ding, X. S. Zhang, *Green Energy Environ.* **2023**, *8*, 798.

[23] Z. C. Gong, P. Z. Chen, H. S. Gong, K. Huang, G. L. Ye, H. L. Fei, *Adv. Mater.* **2024**, 2409292.
